# The European guideline on management of major bleeding and coagulopathy following trauma: fourth edition

**DOI:** 10.1186/s13054-016-1265-x

**Published:** 2016-04-12

**Authors:** Rolf Rossaint, Bertil Bouillon, Vladimir Cerny, Timothy J. Coats, Jacques Duranteau, Enrique Fernández-Mondéjar, Daniela Filipescu, Beverley J. Hunt, Radko Komadina, Giuseppe Nardi, Edmund A. M. Neugebauer, Yves Ozier, Louis Riddez, Arthur Schultz, Jean-Louis Vincent, Donat R. Spahn

**Affiliations:** Department of Anaesthesiology, University Hospital Aachen, RWTH Aachen University, Pauwelsstrasse 30, 52074 Aachen, Germany; Department of Trauma and Orthopaedic Surgery, Witten/Herdecke University, Cologne-Merheim Medical Centre, Ostmerheimer Strasse 200, 51109 Cologne, Germany; Department of Anaesthesiology, Perioperative Medicine and Intensive Care, J.E. Purkinje University, Masaryk Hospital, Usti nad Labem, Socialni pece 3316/12A, 40113 Usti nad Labem, Czech Republic; Department of Research and Development, Charles University in Prague, Faculty of Medicine in Hradec Kralove, Sokolska 581, 50005 Hradec Kralove, Czech Republic; Department of Anaesthesiology and Intensive Care, Charles University in Prague, Faculty of Medicine in Hradec Kralove, Sokolska 581, 50005 Hradec Kralove, Czech Republic; Department of Anaesthesia, Pain Management and Perioperative Medicine, Dalhousie University, Halifax, QE II Health Sciences Centre, 10 West Victoria, 1276 South Park St., Halifax, NS B3H 2Y9 Canada; Emergency Medicine Academic Group, University of Leicester, University Road, Leicester, LE1 7RH UK; Department of Anaesthesia and Intensive Care, Hôpitaux Universitaires Paris Sud, University of Paris XI, Faculté de Médecine Paris-Sud, 78 rue du Général Leclerc, 94275 Le Kremlin-Bicêtre, Cedex France; Servicio de Medicina Intensiva, Complejo Hospitalario Universitario de Granada, ctra de Jaén s/n, 18013 Granada, Spain; Department of Cardiac Anaesthesia and Intensive Care, C. C. Iliescu Emergency Institute of Cardiovascular Diseases, Sos Fundeni 256-258, 022328 Bucharest, Romania; King’s College, Departments of Haematology, Pathology and Lupus, Guy’s and St Thomas’ NHS Foundation Trust, Westminster Bridge Road, London, SE1 7EH UK; Department of Traumatology, General and Teaching Hospital Celje, Oblakova 5, 3000 Celje, Slovenia; Shock and Trauma Centre, S. Camillo Hospital, Viale Gianicolense 87, 00152 Rome, Italy; Faculty of Health - School of Medicine, Witten/Herdecke University, Ostmerheimer Strasse 200, Building 38, 51109 Cologne, Germany; Division of Anaesthesia, Intensive Care and Emergency Medicine, Brest University Hospital, Boulevard Tanguy Prigent, 29200 Brest, France; Department of Surgery and Trauma, Karolinska University Hospital, 171 76 Solna, Sweden; Ludwig Boltzmann Institute for Experimental and Clinical Traumatology, Lorenz Boehler Trauma Centre, Donaueschingenstrasse 13, 1200 Vienna, Austria; Department of Intensive Care, Erasme University Hospital, Université Libre de Bruxelles, Route de Lennik 808, 1070 Brussels, Belgium; Institute of Anaesthesiology, University of Zurich and University Hospital Zurich, Raemistrasse 100, 8091 Zurich, Switzerland

## Abstract

**Background:**

Severe trauma continues to represent a global public health issue and mortality and morbidity in trauma patients remains substantial. A number of initiatives have aimed to provide guidance on the management of trauma patients. This document focuses on the management of major bleeding and coagulopathy following trauma and encourages adaptation of the guiding principles to each local situation and implementation within each institution.

**Methods:**

The pan-European, multidisciplinary Task Force for Advanced Bleeding Care in Trauma was founded in 2004 and included representatives of six relevant European professional societies. The group used a structured, evidence-based consensus approach to address scientific queries that served as the basis for each recommendation and supporting rationale. Expert opinion and current clinical practice were also considered, particularly in areas in which randomised clinical trials have not or cannot be performed. Existing recommendations were reconsidered and revised based on new scientific evidence and observed shifts in clinical practice; new recommendations were formulated to reflect current clinical concerns and areas in which new research data have been generated. This guideline represents the fourth edition of a document first published in 2007 and updated in 2010 and 2013.

**Results:**

The guideline now recommends that patients be transferred directly to an appropriate trauma treatment centre and encourages use of a restricted volume replacement strategy during initial resuscitation. Best-practice use of blood products during further resuscitation continues to evolve and should be guided by a goal-directed strategy. The identification and management of patients pre-treated with anticoagulant agents continues to pose a real challenge, despite accumulating experience and awareness. The present guideline should be viewed as an educational aid to improve and standardise the care of the bleeding trauma patients across Europe and beyond. This document may also serve as a basis for local implementation. Furthermore, local quality and safety management systems need to be established to specifically assess key measures of bleeding control and outcome.

**Conclusions:**

A multidisciplinary approach and adherence to evidence-based guidance are key to improving patient outcomes. The implementation of locally adapted treatment algorithms should strive to achieve measureable improvements in patient outcome.

**Electronic supplementary material:**

The online version of this article (doi:10.1186/s13054-016-1265-x) contains supplementary material, which is available to authorized users.

## Background

Severe trauma is a major global public health issue. Traumatic injury contributes to about one in ten mortalities, resulting in the annual worldwide death of more than 5.8 million people [1, 2], a number that is predicted to increase to >8 million by 2020 [3]. According to the World Health Organization (WHO), road traffic accidents, suicides and homicides are the three leading causes of injury and violence-related deaths [4]. As a consequence, there have been numerous national and international initiatives that aim to prevent violence and traumatic injuries and to provide guidance on the treatment of trauma victims. Uncontrolled post-traumatic bleeding is the leading cause of potentially preventable death among injured patients [5, 6] and the bleeding trauma patient represents a significant financial burden for societies [7], therefore improvements in the management of the massively bleeding trauma patient via educational measures and state-of-the-art clinical practice guidelines should improve outcomes by assisting in the timely identification of bleeding sources, followed by prompt measures to minimise blood loss, restore tissue perfusion and achieve haemodynamic stability.

**Fig. 1:**
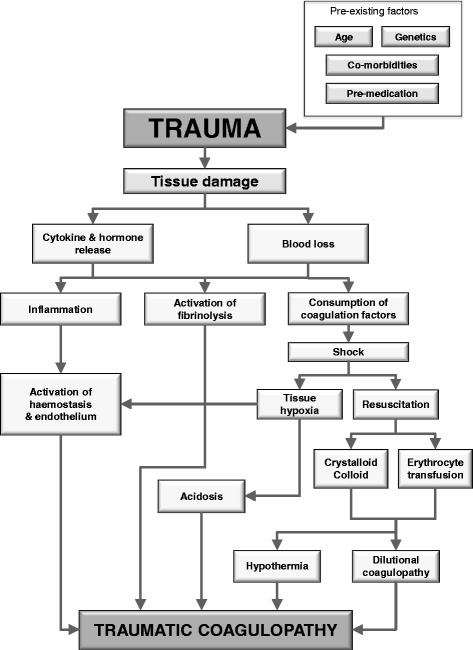
Schematic drawing of the factors, both pre-existing and trauma-related, that contribute to traumatic coagulopathy. Adapted from [18, 19, 34]

Over the past decade the specific pathophysiology associated with bleeding following traumatic injury has been increasingly recognised and management strategies are evolving. Upon hospital admission about one-third of all bleeding trauma patients already show signs of coagulopathy [8–15] and a significant increase in the occurrence of multiple organ failure and death compared to patients with similar injury patterns in the absence of a coagulopathy [8, 9, 11, 16, 17]. The early acute coagulopathy associated with traumatic injury has recently been recognised as a multifactorial primary condition that results from a combination of bleeding-induced shock, tissue injury-related thrombin-thrombomodulin-complex generation and the activation of anticoagulant and fibrinolytic pathways (Fig. 1) [9–11, 14, 18–23]. The severity of the coagulation disorder is influenced by environmental and therapeutic factors that result in, or at least contribute to, acidaemia, hypothermia, dilution, hypoperfusion and coagulation factor consumption [9, 10, 18, 24–26]. Moreover, the coagulopathy is modified by trauma-related factors such as brain injury and individual patient-related factors that include age, genetic background, co-morbidities, inflammation and pre-medication, especially oral anticoagulants, and pre-hospital fluid administration [26–28].

A number of terms have been proposed to describe the specific trauma-associated coagulopathic physiology, including Acute Traumatic Coagulopathy [10, 29], Early Coagulopathy of Trauma [11], Acute Coagulopathy of Trauma-Shock [18], Trauma-Induced Coagulopathy [30] and Trauma-Associated Coagulopathy [31].

This European clinical practice guideline, originally published in 2007 [32] and updated in 2010 [33] and 2013 [34], represents the fourth edition of the guideline and is part of the European “*STOP the Bleeding Campaign*”, an international initiative launched in 2013 to reduce morbidity and mortality associated with bleeding following traumatic injury [35]. With this guideline we aim to achieve a broader awareness of the pathophysiology of the severely bleeding trauma patient and to provide guidance for the clinician by including not only management recommendations but also an overview of the most relevant scientific publications, highlighting areas in which further research is urgently required. We recognise the divergence in international clinical practice in the initial management of patients following traumatic injury, depending on the availability of rapid point-of-care coagulation testing to facilitate goal-directed therapy. Trauma systems without rapid point-of-care testing tend to use fixed ratio protocols during the phase of rapid bleeding, as central laboratory coagulation results are available too late to guide therapy.

Although this set of recommendations outlines corridors for diagnosis and treatment, the author group believes that the greatest outcome improvement can be achieved through education and process adaptation by local clinical management guidelines or algorithms, the use of checklists and management bundles and participation in quality management programmes that contribute to national or international trauma databases. Therefore, this guideline attempts to suggest clinically relevant pathways for diagnosis and therapy in order to facilitate adaptation of the guiding principles to each local situation and implementation within each institution. We believe that adherence to local management guidelines or algorithms should be assessed on a regular basis and will lead, if communicated adequately, to greater adherence. If incorporated into local practice, these clinical guidelines have the potential to ensure a uniform standard of care across Europe and beyond, and better outcomes for the severely bleeding trauma patient.

## Methods

The recommendations made in this guideline are graded according to the Grading of Recommendations Assessment, Development and Evaluation (GRADE) system [36], summarised in Table 1. According to the GRADE scheme, the number associated with each recommendation reflects the strength of the recommendation by the author group, with “we recommend” (Grade 1) being stronger and “we suggest” (Grade 2) being weaker, while the letter reflects the quality of the scientific evidence. Comprehensive, structured, computer-based literature searches were performed using the indexed online database MEDLINE/PubMed, supplemented by screening of reference lists within relevant publications. The aim of each search strategy was to identify randomised controlled trials (RCTs), non-RCTs and systematic reviews that addressed specific scientific queries. In the absence of high-quality scientific support, case reports, observational studies and case control studies were also considered and the literature support for each recommendation graded accordingly.

**Table 1:** Grading of recommendations after [36]. Reprinted with permission

Grade of recommendation	Clarity of risk/benefit	Quality of supporting evidence	Implications
1A			
Strong recommendation, high-quality evidence	Benefits clearly outweigh risk and burdens, or vice versa	RCTs without important limitations or overwhelming evidence from observational studies	Strong recommendation, can apply to most patients in most circumstances without reservation
1B			
Strong recommendation, moderate-quality evidence	Benefits clearly outweigh risk and burdens, or vice versa	RCTs with important limitations (inconsistent results, methodological flaws, indirect or imprecise) or exceptionally strong evidence from observational studies	Strong recommendation, can apply to most patients in most circumstances without reservation
1C			
Strong recommendation, low-quality or very low-quality evidence	Benefits clearly outweigh risk and burdens, or vice versa	Observational studies or case series	Strong recommendation but may change when higher quality evidence becomes available
2A			
Weak recommendation, high-quality evidence	Benefits closely balanced with risks and burden	RCTs without important limitations or overwhelming evidence from observational studies	Weak recommendation, best action may differ depending on circumstances or patients’ or societal values
2B			
Weak recommendation, moderate-quality evidence	Benefits closely balanced with risks and burden	RCTs with important limitations (inconsistent results, methodological flaws, indirect or imprecise) or exceptionally strong evidence from observational studies	Weak recommendation, best action may differ depending on circumstances or patients’ or societal values
2C			
Weak recommendation, low-quality or very low-quality evidence	Uncertainty in the estimates of benefits, risks, and burden; benefits, risk and burden may be closely balanced	Observational studies or case series	Very weak recommendation; other alternatives may be equally reasonable

Boolean operators and medical subject headings (MeSH) were applied to structure each literature search. Appropriate MeSH terms were identified and adjusted if needed to address the scientific queries formulated by the authors. Limitations to the search results included “humans” and “English language”. The time period was limited to 3 years if the query was previously considered in the 2013 guideline. For new queries, the time period was not restricted or limited to 3 or 10 years depending on the number of abstracts identified by each search. The questions addressed the corresponding MeSH terms and the limitations applied to each search are listed in Additional file 1. Abstracts identified by each search strategy were screened by a subset of authors and if considered relevant, full publications were evaluated.

Selection of the scientific queries addressed screening and evaluation of the literature, formulation of the recommendations and the supporting rationales was performed by members of the Task Force for Advanced Bleeding Care in Trauma, which was founded in 2004. The Task Force comprises a multidisciplinary team of pan-European experts representing the fields of emergency medicine, surgery, anaesthesiology, haematology and intensive care medicine. Among the authors are representatives of the European Society for Trauma and Emergency Surgery (ESTES), the European Society of Anaesthesiology (ESA), the European Shock Society (ESS), the European Society for Emergency Medicine (EuSEM), the Network for the Advancement of Patient Blood Management, Haemostasis and Thrombosis (NATA) and the European Society of Intensive Care Medicine (ESICM).

The guideline update process involved several remote (telephone or internet-based) meetings, extensive electronic communication and one face-to-face consensus conference. In January 2015 the authors participated in a web conference during which the queries to be addressed in the updated guideline were defined. Screening and evaluation of abstracts and full publications identified by the structured searches and formulation of draft recommendations and rationales was performed by working subgroups. Each chapter was reviewed by a separate working subgroup and then the entire author group. The wording of each recommendation was finalised during a face-to-face consensus conference that took place in April 2015. After revisions and approval by the author group, the manuscript was approved by the endorsing societies between August 2015 and January 2016. An update of this manuscript is anticipated in due time.

## Results

### I. Initial resuscitation and prevention of further bleeding

#### Minimal elapsed time

##### *Recommendation 1*

**We recommend that severely injured patients be transported directly to an appropriate trauma facility. (Grade 1B)**

**We recommend that the time elapsed between injury and bleeding control be minimised. (Grade 1A)**

#### Rationale

Because relatively few hospitals provide all of the services required to treat patients with multiple injuries, many healthcare systems have developed trauma networks or systems. The underlying aims of trauma care organisation is to move patients to a multi-specialist care as early as possible, yet still provide immediate critical interventions. These aims can come into conflict, and there are a number of different means with which to resolve these issues, resulting in large variations in trauma care systems both between and within countries and a consequent significant heterogeneity in the literature. The evidence is weak, but there is a general consensus that the organisation of a group of hospitals into a “trauma system” leads to about a 15 % reduction in trauma death, with about a 50 % reduction in “preventable death” [37–39]. Inter-hospital transfer of patients does not seem to change overall mortality [40], and the evidence neither supports nor refutes direct transport from the accident scene to a major trauma centre [41]. However, there is some evidence that a lower threshold for trauma centre care should be used in patients aged >65 years [42]. No definitive conclusion can be drawn about the relationship between a hospital’s trauma patient volume and outcomes [43]. Despite a lack of evidence there is a consensus that “systemised” trauma care that matches each patient to the most appropriate treatment facility is advantageous, whereby the definition of “appropriate” will depend on the patient profile, the nature of the injuries and the hospital facilities available.

Trauma patients in need of emergency surgery for ongoing haemorrhage have increased survival if the elapsed time between the traumatic injury and admission to the operating theatre is minimised. More than 50 % of all trauma patients with a fatal outcome die within 24 h of injury [6]. Despite a lack of evidence from prospective RCTs, well-designed retrospective studies provide evidence for early surgical intervention in patients with traumatic haemorrhagic shock [44–46]. In addition, studies that analyse trauma systems indirectly emphasise the importance of minimising the time between admission and surgical bleeding control in patients with traumatic haemorrhagic shock [47, 48]. Minimisation of time to surgery is an accepted principle of trauma care and is unlikely to ever be tested in a clinical trial due to lack of equipoise.

#### Tourniquet use

##### *Recommendation 2*

**We recommend adjunct tourniquet use to stop life-threatening bleeding from open extremity injuries in the pre-surgical setting. (Grade 1B)**

#### Rationale

When uncontrolled arterial bleeding occurs from mangled extremity injuries, including penetrating or blast injuries or traumatic amputations, a tourniquet is a simple and efficient method with which to acutely control haemorrhage [49–53]. Tourniquet application has become standard of care for the control of severe external haemorrhage following military combat injuries, and several publications report the effectiveness of tourniquets in this specific setting in adults [49–52, 54] and children [55]. A study of volunteers showed that any tourniquet device presently on the market works efficiently [53]. The study also showed that “pressure point control” was ineffective because collateral circulation was observed within seconds. Tourniquet-induced pain was not often reported by patients. No evidence or opinion supports the use of tourniquets in the context of closed injuries.

Tourniquets should be left in place until surgical control of bleeding is achieved [50, 52]; however, this time span should be kept as short as possible. Improper or prolonged placement of a tourniquet can lead to complications such as nerve paralysis and limb ischaemia [56], however these effects are rare [54]. Some publications suggest a maximum application time of 2 h [56]. Reports from military settings describe cases in which tourniquets have remained in place for up to 6 h with survival of the extremity [50].

Much discussion has been generated recently about the translation of this evidence to civilian practice, as there is little published evidence. Bleeding from most civilian wounds can be controlled by local pressure, however uncontrolled external bleeding from either blunt [57] or penetrating [58] limb injury should be controlled with a tourniquet.

#### Ventilation

##### *Recommendation 3*

**We recommend the avoidance of hypoxaemia. (Grade 1A)**

**We recommend normoventilation of trauma patients. (Grade 1B)**

**We suggest hyperventilation in the presence of signs of imminent cerebral herniation. (Grade 2C)**

#### Rationale

Tracheal intubation of severely injured patients is a delicate decision that involves risks and requires proper skill and training of the operator. The fundamental objective of intubation is to ensure adequate ventilation, adequate oxygenation and to guarantee the patency of the airway. There are well-defined situations in which intubation is mandatory, for example airway obstruction, altered consciousness [Glasgow Coma Score (GCS) ≤8], haemorrhagic shock, hypoventilation or hypoxaemia [59]; however, other aspects should also be considered. For example, the introduction of positive pressure can induce potentially life-threatening hypotension in hypovolaemic patients [60], and some authors have reported increased mortality associated with pre-hospital intubation [61].

Several factors influence the success of intubation and therefore a patient’s prognosis. Rapid sequence induction appears to be the best method [62], however several aspects remain to be clarified, such as who is best suited to make the decision to intubate, which drugs to use, which rescue device and the ideal infrastructure of emergency services. Most of the available data come from retrospective studies, which are open to bias, therefore controversy remains about the appropriate use of tracheal intubation in patients following traumatic injury [63].

The negative effects of hypoxaemia are well known, particularly in patients with traumatic brain injury (TBI) [64, 65], therefore, high oxygen concentrations are generally used to ensure oxygen delivery to ischaemic areas in the initial management of these patients. Some studies, however, have suggested that the achievement extreme hyperoxia is associated with increased mortality [66]. The reason for this is unclear, but may be related to increased production of free radicals or enhancement of hyperoxic vasoconstriction, hence, avoidance may be prudent. The level of hyperoxia that can become harmful in trauma patients has not been defined, but most studies consider a PaO_2_ above 200–300 mmHg (27–40 kPa) to be too high [67, 68].

Adequate ventilation can affect the outcome of severe trauma patients. There is a tendency for rescue personnel to hyperventilate patients during initial resuscitation [69, 70], and hyperventilated trauma patients appear to have increased mortality when compared with non-hyperventilated patients [66]. Target PaCO_2_ should be 5.0–5.5 kPa (35–40 mmHg).

The effect of hyperventilation on bleeding and outcome in patients with severe trauma without TBI is not known. There are several potential mechanisms by which the adverse effects of hyperventilation and hypocapnia could be mediated, including increased vasoconstriction with decreased cerebral blood flow and impaired tissue perfusion. Cerebral tissue lactic acidosis has been shown to occur almost immediately after induction of hypocapnia in children and adults with TBI and haemorrhagic shock [71]. In addition, an even modest level of hypocapnia [<27 mmHg (3.6 kPa)] may result in neuronal depolarisation with glutamate release and extension of the primary injury via apoptosis [72]. In the setting of absolute or relative hypovolaemia, an excessive rate of positive-pressure ventilation may further compromise venous return and produce hypotension and even cardiovascular collapse [73, 74].

The only situation in which hyperventilation-induced hypocapnia may play a potential role is imminent cerebral herniation. The decrease in cerebral blood flow produced by acute hypocapnia during hyperventilation causes a decrease in intracranial pressure that can be used for short periods of time and in selected cases such as imminent brain herniation. The presence of signs such as unilateral or bilateral pupillary dilation or decerebrate posturing are indicators for an extreme risk of imminent death or irreversible brain damage. Hyperventilation may be used under these circumstances to try to gain time until other measures are effective [75, 76]. There are no clinical studies that evaluate this practice, however, there is a clear physiological rationale. Given the extreme risk of death if no measures are undertaken, the risk–benefit balance seems favourable, however it is important to normalise PaCO_2_ as soon as feasible.

Ventilation with low tidal volume (6 ml/kg) is recommended in patients with or at risk of acute respiratory distress syndrome (ARDS) [77]. In patients with normal lung function, the data is more controversial, but there is increasing evidence to support the idea that the injurious effect of high tidal volume may be initiated very early. Randomised studies demonstrate that short-term ventilation (<5 h) with high tidal volume (12 ml/kg) without positive end-expiratory pressure (PEEP) may promote pulmonary inflammation and alveolar coagulation in patients with normal lung function [78]. Although more studies are needed, the early use of protective ventilation with low tidal volume and moderate PEEP is recommended, particularly in bleeding trauma patients, who are all at risk of ARDS.

### II. Diagnosis and monitoring of bleeding

#### Initial assessment

##### *Recommendation 4*

**We recommend that the physician clinically assess the extent of traumatic haemorrhage using a combination of patient physiology, anatomical injury pattern, mechanism of injury and the patient’s response to initial resuscitation. (Grade 1C)**

#### Rationale

While blood loss may sometimes be obvious, neither visual estimation nor physiological parameters are good guides to the degree of bleeding [79]. The mechanism of injury represents an important screening tool with which to identify patients at risk of significant haemorrhage. For example, the American College of Surgeons defined a threshold of 6 m (20 ft) as a “critical falling height” associated with major injuries [80]. Further critical mechanisms include high-energy deceleration impact, low-velocity versus high-velocity gunshot injuries, etc. The mechanism of injury in conjunction with injury severity and the patient’s physiological presentation and response to resuscitation should further guide the decision to initiate early surgical bleeding control as outlined in the Advanced Trauma Life Support (ATLS) protocol [81–84]. Table 2 summarises estimated blood loss based on initial presentation according to the ATLS classification system. The ATLS classification has been demonstrated to be a useful guide that allows the quantification of blood loss with acceptable accuracy in haemorrhagic shock [85]. However, several groups have highlighted discrepancies associated with the weight assigned each parameter when assessing blood loss that makes it difficult to classify patients using this system. Mutschler et al. analysed the adequacy of this classification and found that more than 90 % of all trauma patients could not be categorised according to the ATLS classification of hypovolaemic shock [86]. The same group analysed the validity of the ATLS classification and concluded that this system may underestimate mental disability in the presence of hypovolaemic shock and overestimate the degree of tachycardia associated with hypotension [87]. A retrospective analysis of the validity of the ATLS classification showed that increasing blood loss produces an increase in heart rate and decrease in blood pressure, but to a lesser degree than suggested by the ATLS classification. In addition, there are no significant changes in respiratory rate or in level of consciousness with bleeding [88]. Table 3 characterises the three types of response to initial fluid resuscitation, whereby the transient responders and the non-responders are candidates for immediate surgical bleeding control.

**Table 2:** American College of Surgeons Advanced Trauma Life Support (ATLS) classification of blood loss^*^ based on initial patient presentation. Table reprinted with permission from the American College of Surgeons [84]

	Class I	Class II	Class III	Class IV
Blood loss (ml)	Up to 750	750–1500	1500–2000	>2000
Blood loss (% blood volume)	Up to 15 %	15–30 %	30–40 %	>40 %
Pulse rate (bpm)	<100	100–120	120–140	>140
Systolic blood pressure	Normal	Normal	Decreased	Decreased
Pulse pressure (mmHg)	Normal or increased	Decreased	Decreased	Decreased
Respiratory rate	14–20	20–30	30–40	>35
Urine output (ml/h)	>30	20–30	5–15	Negligible
CNS/mental status	Slightly anxious	Mildly anxious	Anxious, confused	Confused, lethargic
Initial fluid replacement	Crystalloid	Crystalloid	Crystalloid and blood	Crystalloid and blood

^*^For a 70 kg man

**Table 3:** American College of Surgeons Advanced Trauma Life Support (ATLS) responses to initial fluid resuscitation^*^. Table reprinted with permission from the American College of Surgeons [84]

	Rapid response	Transient response	Minimal or no response
Vital signs	Return to normal	Transient improvement, recurrence of decreased blood pressure and increased heart rate	Remain abnormal
Estimated blood loss	Minimal (10–20 %)	Moderate and ongoing (20–40 %)	Severe (>40 %)
Need for more crystalloid	Low	Low to moderate	Moderate as a bridge to transfusion
Need for blood	Low	Moderate to high	Immediate
Blood preparation	Type and crossmatch	Type-specific	Emergency blood release
Need for operative intervention	Possibly	Likely	Highly likely
Early presence of surgeon	Yes	Yes	Yes

^*^Isotonic crystalloid solution, 2000 ml in adults; 20 ml/kg in children

Specific scores to predict the risk of haemorrhagic shock may be useful to provide prompt and appropriate treatment. The shock index (heart rate divided by systolic blood pressure) may be useful in predicting critical bleeding [89] and can help to identify trauma patients that will require intervention to achieve haemostasis [90]. Paladino et al. [91] analysed the usefulness of the shock index and found that this index may be useful to draw attention to abnormal values, but that it is too insensitive to rule out disease and should not lower the suspicion of major injury. The Trauma-Associated Severe Hemorrhage (TASH) score uses seven parameters [systolic blood pressure, haemoglobin (Hb), intra-abdominal fluid, complex long bone and/or pelvic fractures, heart rate, base excess and gender] to predict the probability of mass transfusion. Maegele et al. [92] retrospectively analysed a dataset of severely multiply injured patients from the German Trauma Registry to confirm the validity of the TASH score to predict the individual probability of massive transfusion and therefore ongoing life-threatening haemorrhage. The TASH score was re-validated with 5834 patients from the same registry [93].

#### Immediate intervention

##### *Recommendation 5*

**We recommend that patients presenting with haemorrhagic shock and an identified source of bleeding undergo an immediate bleeding control procedure unless initial resuscitation measures are successful. (Grade 1B)**

#### Rationale

The source of bleeding may be immediately obvious, and penetrating injuries are more likely to require surgical bleeding control. In a retrospective study of 106 abdominal vascular injuries, all 41 patients arriving in shock following gunshot wounds were candidates for rapid transfer to the operating theatre for surgical bleeding control [94]. A similar observation in a study of 271 patients undergoing immediate laparotomy for gunshot wounds indicates that these wounds combined with signs of severe hypovolaemic shock specifically require early surgical bleeding control. This observation is true to a lesser extent for abdominal stab wounds [95]. Data on injuries caused by penetrating metal fragments from explosives or gunshot wounds in the Vietnam War confirm the need for early surgical control when patients present in shock [96]. In blunt trauma, the mechanism of injury can to a certain extent determine whether the patient in haemorrhagic shock will be a candidate for surgical bleeding control. Only a few studies address the relationship between the mechanism of injury and the risk of bleeding, however, and none of these publications describes a randomised prospective trial with high-level evidence [97]. We have found no objective data describing the relationship between the risk of bleeding and the mechanism of injury resulting in skeletal fractures in general or of long-bone fractures in particular.

Traffic accidents are the leading cause of pelvic injury. Motor vehicle crashes cause approximately 60 % of pelvic fractures followed by falls from great height (23 %). Most of the remainder result from motorbike collisions and vehicle-pedestrian accidents [98, 99]. There is a correlation between “unstable” pelvic fractures and intra-abdominal injuries [98, 100]. An association between major pelvic fractures and severe head injuries, concomitant thoracic, abdominal, urological and skeletal injuries is also well described [98]. High-energy injuries produce greater damage to both the pelvis and organs. Patients with high-energy injuries require more transfusion units, and more than 75 % have associated head, thorax, abdominal or genitourinary injuries [101]. It is well documented that ‘unstable’ pelvic fractures are associated with massive haemorrhage [100, 102], and haemorrhage is the leading cause of death in patients with major pelvic fractures. Vertical shear pelvic ring fractures with caudal displacement of the hemi-pelvis may disrupt the pelvic floor and pelvic vasculature far more than standard vertical shear injuries. Inferior displacement of the hemi-pelvis using X-ray imaging should therefore alert the surgeon to the possible presence of severe arterial injuries [103].

In blunt chest trauma haemothoraces >500 ml should trigger chest tube insertion. Thoracotomy is indicated for ongoing bleeding and chest tube output >1500 ml within 24 h or >200 ml for 3 consecutive hours. Acute damage control thoracotomy should be performed for refractive haemorrhagic shock due to persistent chest bleeding enhanced by initial chest tube output >1500 ml [104, 105].

#### Further investigation

##### *Recommendation 6*

**We recommend that patients presenting with haemorrhagic shock and an unidentified source of bleeding undergo immediate further investigation. (Grade 1B)**

#### Rationale

A patient in haemorrhagic shock with an unidentified source of bleeding should undergo immediate further assessment of chest, abdominal cavity and pelvic ring, which represent the major sources of acute blood loss in trauma. Aside from a clinical examination, X-rays of chest and pelvis in conjunction with ultrasonography [106] are recommended diagnostic modalities during the primary survey [84, 107, 108].

In selected centres, readily available computed tomography (CT) scanners [109] may replace conventional radiographic imaging techniques during the primary survey. Huber-Wagner et al. analysed the effect of the distance between the trauma room and the CT scanner on the outcome in a multicentre study involving 8004 adult major blunt trauma patients at 312 hospitals and showed that close proximity of the CT scanner to the trauma room has a significant positive effect on the survival of severely injured patients. The authors suggest that emergency department planning place the CT scanner in the trauma room or within 50 meters [110]. In their systematic literature review, Jorgensen and colleagues found no evidence that pre-hospital ultrasound of the abdomen or chest improves the treatment of trauma patients [111].

#### Imaging

##### *Recommendation 7*

**We recommend early imaging (ultrasonography or contrast-enhanced CT) for the detection of free fluid in patients with suspected torso trauma. (Grade 1B)**

#### Intervention

##### *Recommendation 8*

**We recommend that patients with significant intra-thoracic, intra-abdominal or retroperitoneal bleeding and haemodynamic instability undergo urgent intervention. (Grade 1A)**

#### Further assessment

##### *Recommendation 9*

**We recommend CT assessment for haemodynamically stable patients. (Grade 1B)**

#### Rationale

Blunt abdominal trauma represents a major diagnostic challenge and an important source of internal bleeding. Ultrasonography has been established as a rapid and non-invasive diagnostic approach for the detection of intra-abdominal free fluid in the emergency room [112–114]. Large prospective observational studies determined a high specificity and accuracy but low sensitivity of initial ultrasonographic examination for detecting intra-abdominal injuries in adults and children [115–121]. Liu and colleagues [122] found a high sensitivity, specificity and accuracy of initial ultrasound examination for the detection of haemoperitoneum. Ultrasonography has a high specificity but a low sensitivity for detecting free intraperitoneal fluid in penetrating torso trauma [123] and in blunt abdominal trauma in children [124]. A positive ultrasound suggests haemoperitoneum, but a negative initial abdominal ultrasound should direct further diagnostic investigations.

The role of CT scanning in acute trauma patients is well documented [125–132], and in recent years imaging for trauma patients has migrated towards multislice computed tomography (MSCT). The integration of modern MSCT scanners in the emergency room area allows the immediate assessment of trauma victims following admission [127, 128]. Using modern MSCT scanners, total whole-body scanning time may be reduced to less than 30 seconds. In a retrospective study comparing 370 patients in two groups, Weninger and colleagues [128] showed that faster diagnosis using MSCT led to shorter emergency room and operating room time and shorter intensive care unit (ICU) stays [128]. Huber-Wagner et al. [109] also showed the benefit of integration of the whole-body CT into early trauma care. CT diagnosis significantly increases the probability of survival in patients with polytrauma [110]. Whole-body CT as a standard diagnostic tool during the earliest resuscitation phase for polytraumatised patients provides the added benefit of identifying head and chest injuries and other bleeding sources in multiply injured patients.

Some authors have shown the benefit of contrast medium-enhanced CT scanning. Anderson et al. [133, 134] found high accuracy in the evaluation of splenic injuries resulting from trauma after administration of intravenous (i.v.) contrast material. Delayed-phase CT may be used to detect active bleeding in solid organs. Fang et al. [135] demonstrated that the pooling of contrast material within the peritoneal cavity in blunt liver injuries indicates active and massive bleeding. Patients with this finding showed rapid deterioration of haemodynamic status, and most required emergent surgery. Intraparenchymal pooling of contrast material with an unruptured liver capsule often indicates a self-limited haemorrhage, and these patients respond well to non-operative treatment. Tan and colleagues [136] found that patients with hollow viscus and mesenteric injuries following blunt abdominal trauma exhibited an abnormal preoperative CT scan. Wu et al. [137] showed the accuracy of CT in identifying severe, life-threatening mesenteric haemorrhage and blunt bowel injuries.

Compared to MSCT, all traditional techniques for diagnostic and imaging evaluation are associated with some limitations. The diagnostic accuracy, safety and effectiveness of immediate MSCT are dependent on sophisticated pre-hospital treatment by trained and experienced emergency personnel and short transportation times [138, 139]. If an MSCT is not available in the emergency room, the realisation of CT scanning implies transportation of the patient to the CT room, therefore the clinician must evaluate the implications and potential risks and benefits of the procedure. During transport, all vital signs should be closely monitored and resuscitation measures continued. For those patients in whom haemodynamic stability is questionable, imaging techniques such as ultrasound and chest and pelvic radiography may be useful. Peritoneal lavage is rarely indicated if ultrasound or CT are available [140]. Transfer times to and from all forms of diagnostic imaging need to be considered carefully in any patient who is haemodynamically unstable. In addition to the initial clinical assessment, point-of-care testing results, including full blood count, haematocrit (Hct), blood gases, and lactate, should be readily available under ideal circumstances.

The hypotensive patient (systolic blood pressure below 90 mmHg) presenting free intra-abdominal fluid according to ultrasonography or CT is a potential candidate for early surgical intervention if he or she cannot be stabilised by initiated fluid resuscitation [141–143]. A retrospective study by Rozycki and colleagues [144] of 1540 patients (1227 blunt, 313 penetrating trauma) assessed with ultrasound as an early diagnostic tool showed that the ultrasound examination had a sensitivity and specificity close to 100 % when patients were hypotensive.

A number of patients who present with free intra-abdominal fluid according to ultrasound can safely undergo further investigation using MSCT. Under normal circumstances, adult patients need to be haemodynamically stable when MSCT is performed outside of the emergency room [144]. Haemodynamically stable patients with a high-risk mechanism of injury, such as high-energy trauma or even low-energy injuries in elderly individuals, should be scanned after ultrasound for additional injuries using MSCT. As CT scanners are integrated in resuscitation units, whole-body CT diagnosis may replace ultrasound as a diagnostic method.

MSCT is the gold standard for the identification of retroperitoneal haemorrhage (RPH). After injection of i.v. contrast solution, CT identified RPH in all cases (100 %) and may show the source of bleeding (40 %) by extravasation of contrast media [145].

Haemodynamically unstable patients with significant intrathoracic, intra-abdominal or retroperitoneal bleeding may need urgent intervention. In these cases with thoracic trauma and chest bleeding the insertion of a chest tube is the first surgical step, usually just prior to acute damage control thoracotomy. Surgical bleeding control is necessary in unstable patients presenting with haemoperitoneum. Patients with pelvic trauma and significant retroperitoneal haematoma may need external compression, retroperitoneal packing or urgent radiologic embolisation for pelvic haemorrhage control [146–148].

#### Haemoglobin

##### *Recommendation 10*

**We recommend that a low initial Hb be considered an indicator for severe bleeding associated with coagulopathy. (Grade 1B)**

**We recommend the use of repeated Hb measurements as a laboratory marker for bleeding, as an initial Hb value in the normal range may mask bleeding. (Grade 1B)**

#### Rationale

Hb or Hct assays are part of the basic diagnostic work-up for trauma patients. Currently the use of Hb rather than Hct is widespread, and the latter is a calculated parameter derived from the Hb. However, most studies on which these recommendations are based analysed Hct rather than Hb. Because both parameters are used interchangeably in clinical practice, in these guidelines we refer to both parameters according to the parameter described by the literature to which we refer.

The diagnostic value of the Hb or Hct for detecting trauma patients with severe injury and occult bleeding sources has been a topic of debate [149–151]. A major limit of the Hb/Hct’s diagnostic value is the confounding influence of resuscitation measures on the Hb/Hct due to administration of i.v. fluids and erythrocyte concentrates [152–154]. In addition, initial Hb or Hct may not accurately reflect blood loss because patients bleed whole blood and compensatory mechanisms that move fluids from interstitial space require time and may not be reflected in initial measurements. The concept of the low sensitivity of initial Hb/Hct for the detection of severe bleeding has been challenged. In a retrospective study of 196 trauma patients, Ryan et al. [155] found that Hct at admission closely correlates with haemorrhagic shock. Other authors also recommended that the initial Hct play a greater role in the assessment of blood loss in trauma patients. In a retrospective analysis of 1492 consecutive trauma patients Thorson et al. found that the initial Hct is associated more strongly with the need for transfusion than other parameters such as heart rate, blood pressure or acidaemia, suggesting that fluid shifts are rapid after trauma and imply a more important role for Hct in the initial assessment of trauma victims [156]. An initial low Hb level is one of the predictive criteria for massive transfusion using the TASH [92] and Vandromme [157] scores.

Thorson et al. [158] analysed changes in Hct in two successive determinations and concluded that the change in Hct is a reliable parameter with which to detect blood loss. Two prospective observational diagnostic studies also showed the sensitivity of serial Hct measurements in the detection of patients with severe injury [149, 150]. Decreasing serial Hct measurements may reflect continued bleeding; however the patient with significant bleeding may maintain the serial Hct in the context of ongoing resuscitation and physiological compensatory mechanisms. Acute anaemia may play an adverse role in the clotting process because a low Hct may reduce platelet marginalisation with a potentially negative impact on platelet activation. Moreover Schlimp et al. [159] demonstrated that levels of fibrinogen lower than 150 mg/dl are detected in as many as 73 % of the patients with admission Hb lower than 10 g/dl.

#### Serum lactate and base deficit

##### *Recommendation 11*

**We recommend serum lactate and/or base deficit measurements as sensitive tests to estimate and monitor the extent of bleeding and shock. (Grade 1B)**

#### Rationale

Serum lactate has been used as a diagnostic parameter and prognostic marker of haemorrhagic shock since the 1960s [160]. The amount of lactate produced by anaerobic glycolysis is an indirect marker of oxygen debt, tissue hypoperfusion and the severity of haemorrhagic shock [161–164]. Similarly, base deficit values derived from arterial blood gas analysis provide an indirect estimation of global tissue acidosis due to impaired perfusion [161, 163]. Vincent and colleagues [165] showed the value of serial lactate measurements for predicting survival in a prospective study in patients with circulatory shock. This study showed that changes in lactate concentration provide an early and objective evaluation of a patient’s response to therapy and suggested that repeated lactate determinations represent a reliable prognostic index for patients with circulatory shock [165]. Abramson and colleagues [166] performed a prospective observational study in patients with multiple traumatic injuries to evaluate the correlation between lactate clearance and survival. All patients in whom lactate levels returned to the normal range (≤2 mmol/l) within 24 h survived. Survival decreased to 77.8 % if normalisation occurred within 48 h and to 13.6 % in those patients in whom lactate levels were elevated above 2 mmol/l for more than 48 h [166]. These findings were confirmed in a study by Manikis et al. [167], who showed that initial lactate levels were higher in non-survivors after major trauma and that prolongation of time to normalisation of lactate levels of more than 24 h was associated with the development of post-traumatic organ failure [167]. The determination of lactate and/or base deficit may be particularly important in penetrating trauma. In this type of trauma, triage vital signs such as blood pressure, heart rate and respiratory rate do not reflect the severity of injury and are not related to lactate or base deficit levels [168].

The reliability of lactate determination may be lower when traumatic injury is associated with alcohol consumption. Ethanol metabolism induces the conversion of pyruvate to lactate via lactate dehydrogenase, causing an increase in the level of lactate in the blood. In alcohol-associated trauma, therefore, base deficit may be a better predictor of prognosis than lactate [169], although some authors suggest that ethanol-induced acidosis may also affect base deficit, masking the prognosis of trauma patients [170]. Therefore, in the case of traumatic injury associated with alcohol consumption, the results of the lactate measurements should be interpreted with caution.

Similar to the predictive value of lactate levels, the initial base deficit, obtained either from arterial or peripheral venous blood [171] has been established as a potent independent predictor of mortality in patients with traumatic haemorrhagic shock [169]. Davis and colleagues [172] stratified the extent of base deficit into three categories: mild (-3 to -5 mEq/l), moderate (-6 to -9 mEq/l) and severe (<-10 mEq/l), and established a significant correlation between the admission base deficit, transfusion requirements within the first 24 h and the risk of post-traumatic organ failure or death [172]. The same group of authors showed that the base deficit is a better prognostic marker of death than the pH in arterial blood gas analyses [173]. Mutschler et al. [174] analysed a cohort of 16,305 severely injured patients derived from the German Trauma Registry database and concluded that the determination of base deficit upon emergency department admission predicts transfusion requirements and mortality better than ATLS classification [174]. Furthermore, the base deficit was shown to represent a highly sensitive marker for the extent of post-traumatic shock and mortality, both in adult and paediatric patients [175, 176].

In contrast to the data on lactate levels in haemorrhagic shock, reliable large-scale prospective studies on the correlation between base deficit and outcome are still lacking. Although both the base deficit and serum lactate levels are well correlated with shock and resuscitation, these two parameters do not strictly correlate with each other in severely injured patients [177]. Therefore, the independent assessment of both parameters is recommended for the evaluation of shock in trauma patients [161, 163, 177].

#### Coagulation monitoring

##### *Recommendation 12*

**We recommend that routine practice include the early and repeated monitoring of coagulation, using either a traditional laboratory determination [prothrombin time (PT), activated partial thromboplastin time (APTT) platelet counts and fibrinogen] (Grade 1A) and/or a viscoelastic method. (Grade 1C)**

#### Rationale

Standard coagulation monitoring comprises the early and repeated determination of PT, APTT, platelet counts and fibrinogen. Increasing emphasis focuses on the importance of fibrinogen and platelet measurements. It is often assumed that the conventional coagulation screens [international normalised ratio (INR) and APTT] monitor coagulation, however these tests monitor only the initiation phase of blood coagulation, and represent only the first 4 % of thrombin production [178]. It is therefore possible that the conventional coagulation screen appears normal, while the overall state of blood coagulation is abnormal [13, 179–183]. In addition, the delay in detection of traumatic coagulopathy can influence outcome, and the turnaround time of thromboelastometry has been shown to be significantly shorter than conventional laboratory testing, with a time saving of 30–60 min [181, 184, 185]. Viscoelastic testing may also be useful in the detection of coagulation abnormalities associated with the use of direct thrombin inhibitors such as dabigatran, argatroban, bivalirudin or hirudin. Furthermore, (early) variables of clot firmness assessed by viscoelastic testing have been shown to be good predictors for the need for massive transfusion, the incidence of thrombotic/thromboembolic events and for mortality in surgical and trauma patients [181, 186–195]. Therefore, complete and rapid monitoring of blood coagulation and fibrinolysis using viscoelastic methods may facilitate a more accurate targeting of therapy compared to conventional laboratory tests alone.

Tools such as thromboelastometry and portable coagulometers have been developed to detect coagulopathy in the emergency room or at the bedside, improving the availability of real-time data to guide patient management. Portable coagulometers that provide INR or APTT seem to provide acceptable accuracy for point-of-care INR testing in the emergency department compared with laboratory-based methods [196–198], however others have observed a lack of agreement with conventional laboratory determinations [199]. The usefulness of the parameters measured is therefore limited.

Viscoelastic methods provide a rapid assessment of coagulation to support clinical decision-making, generating a growing confidence in these methods and increased use [200, 201]. Case series using viscoelastic testing to assess trauma patients have been published. One study applied rotational thrombelastography to 23 patients, but without a comparative standard [179]. Johansson et al. [180] implemented a haemostatic resuscitation regime [early platelets and fresh frozen plasma (FFP)] guided using thrombelastography in a before-and-after study (n = 832), which showed improved outcomes. In a retrospective study of cardiovascular surgery patients (n = 3865) the combined use of thromboelastometry and portable coagulometry resulted in a reduction in blood product transfusion and thromboembolic events, but did not influence mortality [202]. Rapid thrombelastography is a new variant of viscoelastic testing in which coagulation is initiated by the addition of kaolin and tissue factor that appears to reduce the measurement time compared with conventional thrombelastography [203].

Despite the widespread use of viscoelastic methods, the usefulness has recently been questioned. In a recent systematic review Hunt et al. [204] found no evidence of the accuracy of thrombelastography and very little evidence to support the accuracy of thromboelastometry and were therefore unable to offer any advice about the use of these methods [204]. In another systematic review Da Luz et al. [205] concluded that only limited evidence from observational studies support the use of viscoelastic tests to diagnose early traumatic coagulopathy, but while these tests may predict blood-product transfusion, mortality and other patient-important outcomes may be unaffected [205]. A number of other limitations to the use of viscoelastic methods have been described. Larsen et al. [206] found that thrombelastography was unable to distinguish coagulopathies caused by dilution from thrombocytopenia, whereas thromboelastometry was indeed capable of distinguishing these two different types of coagulopathy and suggesting the correct treatment [206]. The use of thrombelastography may thus lead to unnecessary transfusion with platelets, whereas the application of thromboelastometry may result in goal-directed fibrinogen substitution. Although use is rapidly increasing, controversy remains at present regarding the utility of viscoelastic methods for the detection of post-traumatic coagulopathy.

The agreement between viscoelastic methods and standard coagulation test also remains a matter of debate. Some studies find acceptable agreement [207–209], however a number of other studies found significant discrepancies [25, 199, 210, 211] even among different viscoelastic methods (thrombelastography and thromboelastometry). Hagemo et al. [212] found that the correlation was highly variable at different stages of the clotting process and between centres, highlighting the need for clarification and standardisation of these techniques. One limitation of viscoelastic tests is the lack of sensitivity to detect and monitor platelet dysfunction due to antiplatelet drugs. If platelet dysfunction is expected, point-of-care platelet function tests, for example whole blood impedance aggregometry, should be used in addition to viscoelastic tests [213, 214]. More research is required in this area, and in the meantime physicians should use their own judgement when developing local policies.

It is theoretically possible that the pattern of change in measures of coagulation such as D-dimers may help to identify patients with ongoing bleeding. However, a single publication showed that the positive predictive value of D-dimers is only 1.8 % in the postoperative and/or post-traumatic setting [215], therefore traditional methods of detection for ongoing bleeding, such as serial clinical evaluation of radiology (ultrasound, CT or angiography) should be used.

### III. Tissue oxygenation, type of fluid and temperature management

#### Tissue oxygenation

##### *Recommendation 13*

**We recommend a target systolic blood pressure of 80**–**90 mmHg until major bleeding has been stopped in the initial phase following trauma without brain injury. (Grade 1C)**

**In patients with severe TBI (GCS ≤8), we recommend that a mean arterial pressure ≥80 mmHg be maintained. (Grade 1C)**

#### Restricted volume replacement

##### *Recommendation 14*

**We recommend use of a restricted volume replacement strategy to achieve target blood pressure until bleeding can be controlled. (Grade 1B)**

#### Vasopressors and inotropic agents

##### *Recommendation 15*

**In the presence of life-threatening hypotension, we recommend administration of vasopressors in addition to fluids to maintain target arterial pressure. (Grade 1C)**

**We recommend infusion of an inotropic agent in the presence of myocardial dysfunction. (Grade 1C)**

#### Rationale

In order to maintain tissue oxygenation, traditional treatment of trauma patients used early and aggressive fluid administration to restore blood volume. This approach may, however, increase the hydrostatic pressure on the wound, cause dislodgement of blood clots, a dilution of coagulation factors and undesirable cooling of the patient. The concept of “damage control resuscitation” aims to achieve a lower than normal blood pressure, also called “permissive hypotension”, and thereby avoid the adverse effects of early aggressive resuscitation using high doses of fluids while there is a potential risk of tissue hypoperfusion during short periods [216]. The general effectiveness of permissive hypotension remains to be confirmed in randomised clinical trials, however, two studies published in the 1990s demonstrated increased survival when a low and delayed fluid volume resuscitation concept was used in penetrating [217] or penetrating and blunt [218] trauma. However, in contrast to these studies, no significant differences in survival were found in two further trials in patients with either penetrating and blunt trauma [219] or blunt trauma alone [220].

Several retrospective analyses published in the last few years demonstrated that aggressive resuscitation techniques, often initiated in the pre-hospital setting, may be detrimental for trauma patients [9, 28, 221, 222]. One of these studies showed that this strategy increased the likelihood that patients with severe extremity injuries developed secondary abdominal compartment syndrome (ACS) [221]. In that study, early large-volume crystalloid administration was the greatest predictor of secondary ACS. Moreover, another retrospective analysis using the German Trauma Registry database, including 17,200 multiply injured patients, showed that the incidence of coagulopathy increased with increasing volume of i.v. fluids administered pre-clinically [9]. Coagulopathy was observed in >40 % of patients with >2000 ml, in >50 % with >3000 ml and in >70 % with >4000 ml administered. Using the same trauma registry, a retrospective matched pairs analysis (n = 1896) demonstrated that multiply injured trauma patients with an Injury Severity Score (ISS) ≥16 points and a systolic blood pressure ≥60 mmHg at the accident site who received pre-hospital low-volume resuscitation (0–1500 ml) had a higher survival rate than patients in whom a pre-hospital high-volume strategy (≥1501 ml) was used [28]. These results are supported by another retrospective analysis of patients from the US National Trauma Data Bank [222]. In this study the authors analysed 776,734 patients, of whom about 50 % received pre-hospital i.v. fluid and 50 % did not. The group of patients receiving preoperative i.v. fluids were significantly more likely to die (OR 1.11, 95 % CI 1.05 to 1.17), an association which was especially marked in patients with penetrating mechanisms of injury (OR 1.25, 95 % CI 1.08 to 1.45), hypotension (OR 1.44, 95 % CI 1.29 to 1.59), severe head injury (OR 1.34, 95 % CI 1.17 to 1.54) and patients undergoing immediate surgery (OR 1.35, 95 % CI 1.22 to 1.50). The authors concluded that the routine use of pre-hospital i.v. fluid for all trauma patients should be discouraged. It should be noted that this study, and especially its conclusion, has been criticised [223].

Initial use of a restrictive volume replacement strategy is supported by a prospective randomised trial that analysed the consequences of an initial intra-hospital hypotensive resuscitation strategy in trauma patients with haemorrhagic shock [224]. In this study, with nearly all of the 90 patients suffering from penetrating trauma, patients who had at least one documented in-hospital systolic blood pressure ≤90 mmHg were randomised to a target minimum mean arterial pressure of 50 mmHg or 65 mmHg. One major drawback to this study was that no statistically significant difference between the actual mean arterial pressure was observed between the two groups over the duration of the study (64.4 mmHg vs. 68.5 mmHg, *P* = 0.15). Although the authors could not demonstrate a survival difference for the two treatment strategies at day 30, 24 h postoperative death and coagulopathy were increased in the group with the higher target minimum pressure. The patients in this group received not only more i.v. fluids overall, but also more blood product transfusions. Another study that supports a restrictive volume replacement strategy was reported by Brown et al. [225]. In this study 1216 trauma patients with an ISS >15 were included; 51 % suffered from hypotension, defined as a systolic arterial blood pressure (SAP) <90 mmHg. 68 % of the patients received a volume load of >500 ml crystalloid solution. The authors demonstrated that administration of >500 ml pre-hospital crystalloid was associated with worse outcome in patients without pre-hospital hypotension but not in patients with hypotension. The administration of >500 ml crystalloid was associated with a correction of hypotension. The authors suggested that pre-hospital volume resuscitation should be goal-directed based on the presence or absence of hypotension. Recently, Schreiber et al. [226] assessed the feasibility and safety of controlled resuscitation (n = 97) in hypotensive trauma patients compared to standard resuscitation (n = 95). Patients were enrolled and randomised in the pre-hospital setting. Eligible patients had a pre-hospital systolic blood pressure ≤90 mmHg. Controlled resuscitation patients received 250 ml fluid if no radial pulse or an SAP <70 mmHg was present and additional 250 ml boluses to maintain a radial pulse or a systolic blood pressure ≥70 mmHg. The mean (SD) crystalloid volume administered during the study period was 1.0 l (1.5) in the controlled resuscitation group and 2.0 l (1.4) in the standard resuscitation group. ICU-free days, ventilator-free days, renal injury and renal failure did not differ between the groups.

A meta-analysis by Kwan et al. analysed randomised trials that investigated the timing and volume of i.v. fluid administration in bleeding trauma patients [227]. The authors identified three trials that addressed the timing of administration and that included a total of 1957 patients. Three studies investigated volume load, but included only 171 patients. In contrast to the retrospective analysis described above, the meta-analysis failed to demonstrate an advantage associated with delayed compared to early fluid administration nor of smaller compared to larger volume fluid administration in this small group of prospective studies that included only a very limited number of patients. A further meta-analysis that assessed seven retrospective observational studies that included a total of 13,687 patients and three prospective studies that included 798 patients estimated a small benefit in favour of a restricted volume replacement strategy [228], however, the authors cautioned that the available studies were subject to a high risk of selection bias and clinical heterogeneity.

It should be noted that a damage control resuscitation strategy using restrictive volume replacement is contraindicated in patients with TBI and spinal injuries, because an adequate perfusion pressure is crucial to ensure tissue oxygenation of the injured central nervous system [229]. Rapid bleeding control is of particular importance in these patients. In addition, the concept of permissive hypotension should be carefully considered in the elderly patient, and may be contraindicated if the patient suffers from chronic arterial hypertension [230].

In conclusion, a damage control resuscitation strategy that aims to achieve a lower than normal systolic blood pressure of 80–90 mmHg using a concept of restricted fluid replacement in patients without TBI and/or spinal injury is supported by the literature, however strong evidence from RCTs is lacking.

Vasopressors may also be required transiently to sustain life and maintain tissue perfusion in the presence of life-threatening hypotension, even when fluid expansion is in progress and hypovolaemia has not yet been corrected. Norepinephrine (NE) is often used to restore arterial pressure in septic and haemorrhagic shock and is now recommended as the agent of choice for this purpose during septic shock [231]. Although NE has some β-adrenergic effects, it acts predominantly as a vasoconstrictor. Arterial α-adrenergic stimulation increases arterial resistance and may increase cardiac afterload; NE exerts both arterial and venous α-adrenergic stimulation [232]. Indeed, in addition to its arterial vasoconstrictor effect, NE induces venoconstriction at the level of the splanchnic circulation in particular, which increases the pressure in capacitance vessels and actively shifts splanchnic blood volume to the systemic circulation [233]. This venous adrenergic stimulation may recruit some blood from the venous unstressed volume, i.e., the volume that fills the blood vessels without generating intravascular pressure. Moreover, stimulation of β_2_-adrenergic receptors decreases venous resistance and increases venous return [233].

Animal studies that investigated uncontrolled haemorrhage have suggested that NE infusion reduces the amount of fluid resuscitation required to achieve a given arterial pressure target, is associated with lower blood loss and significantly improved survival [234, 235]. However, the effects of NE have not been rigorously investigated in humans during haemorrhagic shock. An interim analysis performed during an ongoing multicentre prospective cohort study suggested that the early use of vasopressors for haemodynamic support after haemorrhagic shock may be deleterious in comparison to aggressive volume resuscitation and should be used cautiously [236]. This study has several limitations, however. First, this was a secondary analysis of a prospective cohort study and was not designed to answer the specific hypothesis tested, and second, the group receiving vasopressors had a higher rate of thoracotomy. Thus, a prospective study to define the effect of vasopressors on patients during haemorrhagic shock is clearly needed.

A double-blind randomised trial to assess the safety and efficacy of adding vasopressin to resuscitative fluid has been performed [237]. Patients were given fluid alone or fluid plus vasopressin (bolus 4 IU) and i.v. infusion of 200 ml/h (vasopressin 2.4 IU/h) for 5 h. The fluid plus vasopressin group needed a significantly lower total resuscitation fluid volume over 5 days than the control group (*P* = 0.04). The rates of adverse events, organ dysfunction and 30-day mortality were similar.

Vasopressors may be useful if used transiently to sustain arterial pressure and maintain tissue perfusion in the face of life-threatening hypotension. If used, it is essential to respect the recommended objectives for SAP (80–90 mmHg) in patients without TBI.

Because vasopressors may increase cardiac afterload if the infusion rate is excessive or left ventricular function is already impaired, an assessment of cardiac function during the initial ultrasound examination is essential. Cardiac dysfunction could be altered in the trauma patient following cardiac contusion, pericardial effusion or secondary to brain injury with intracranial hypertension. The presence of myocardial dysfunction requires treatment with an inotropic agent such as dobutamine or epinephrine. In the absence of an evaluation of cardiac function or cardiac output monitoring, as is often the case in the early phase of haemorrhagic shock management, cardiac dysfunction must be suspected in the presence of a poor response to fluid expansion and NE.

#### Type of fluid

##### *Recommendation 16*

**We recommend that fluid therapy using isotonic crystalloid solutions be initiated in the hypotensive bleeding trauma patient. (Grade 1A)**

**We suggest that excessive use of 0.9 % NaCl solution be avoided. (Grade 2C)**

**We recommend that hypotonic solutions such as Ringer’s lactate be avoided in patients with severe head trauma. (Grade 1C)**

**We suggest that the use of colloids be restricted due to the adverse effects on haemostasis. (Grade 2C)**

#### Rationale

Although fluid resuscitation is the first step to restore tissue perfusion in severe haemorrhagic shock, it is still unclear whether crystalloids or colloids, and more specifically which crystalloid or which colloid, should be used in the initial treatment of the bleeding trauma patient.

In most trauma studies 0.9 % sodium chloride was used as the crystalloid solution. However, recent studies suggest that this crystalloid may increase acidosis and the incidence of kidney injury in healthy volunteers or critically ill adults [238, 239]. In contrast to 0.9 % sodium chloride, balanced electrolyte solutions contain physiological or near-physiological concentrations of electrolytes. Recently, in a small prospective randomised trial in 46 trauma patients a balanced electrolyte solution improved acid-base status and caused less hyperchloraemia at 24 h post injury compared to 0.9 % sodium chloride [240]. A secondary analysis of this study demonstrated that the use of a balanced electrolyte solution resulted in a net cost benefit in comparison to the use of 0.9 % saline chloride [241]. Therefore, if 0.9 % sodium chloride is used it should be limited to a maximum of 1–1.5 l.

If crystalloids are used, hypotonic solutions such as Ringer’s lactate should be avoided in patients with TBI in order to minimise a fluid shift into the damaged cerebral tissue. In addition, the use of solutions with the potential to restore pH may be advantageous, since a recent study demonstrated that Ringer’s acetate solution more rapidly ameliorated splanchnic dysoxia, as evidenced by gastric tonometry, than Ringer’s lactate [242]. Whether an advantage for certain isotonic balanced crystalloids with respect to a reduced morbidity or mortality exists is not clear and remains to be evaluated [241, 243].

The most recent Cochrane meta-analysis on the type of fluid, colloids or crystalloids, failed to demonstrate that colloids reduce the risk of death compared to resuscitation with crystalloids in critically ill patients treated in an ICU [244]. The authors compared the use of albumin or plasma protein fraction with crystalloids, performing an analysis of 24 trials that included a total of 9920 patients, and demonstrated a pooled risk ratio (RR) of 1.01 (95 % CI 0.93 to 1.10). Twenty-five trials compared hydroxyethyl starch (HES) to crystalloids in a total of 9147 patients, demonstrating a beneficial effect in favour of crystalloids [RR 1.10 (1.02 to 1.19)], and modified gelatin was assessed in 11 trials that included a total of 506 patients showing neither a beneficial nor a deleterious effect [RR 0.91 (0.49 to 1.72)]. The authors concluded that there is no evidence that resuscitation with colloids has any beneficial effect on survival, and HES may even cause harm. However, neither the time point of fluid resuscitation nor the duration and dosages of fluid resuscitation were analysed or discussed. Nevertheless, at the present time good data demonstrating the benefit of colloids are lacking.

Since colloids are also more expensive than crystalloids, if fluids are used during the initial treatment phase as part of the restricted volume replacement strategy, administration of crystalloids rather than colloids to treat the hypotensive bleeding trauma patient seems to be justified. Also in later stages of resuscitation, large volume crystalloid administration is not independently associated with multiple organ failure [245]. In addition, if high ratios of FFP:RBC (red blood cells) cannot be administered to trauma patients, a retrospective study showed that resuscitation with at least 1 l crystalloid per unit RBC seems to be associated with reduced overall mortality [246].

At present it is not clear whether, and if, which colloids should be used if crystalloids fail to restore target blood pressure. Bunn et al. published a Cochrane meta-analysis with the aim of comparing the effects of different colloid solutions in a total of 5484 patients thought to require volume replacement [247]. From this review, there is no evidence that one colloid solution is more effective or safer than any other, although the confidence intervals were wide and do not exclude clinically significant differences between colloids. Nevertheless, there are conflicting meta-analysis data showing on the one hand increased kidney injury and increased mortality in critically ill patients treated with HES [248, 249] and on the other hand no differences in the incidence of death or acute kidney failure in surgical patients receiving 6 % HES [250]. It seems doubtful whether any conclusions can be drawn from these studies performed mostly under completely different conditions than are present in the acute hypovolaemic trauma patient. In addition to these conflicting results, a recent in vitro study using blood from healthy volunteers demonstrated that coagulation and platelet function are impaired by all HES and gelatin solutions [251]. However, gelatin-induced coagulopathy was reversible with the administration of fibrinogen, whereas HES-induced coagulopathy was not. So far, only one small RCT described a benefit for a HES solution in trauma patients. HES (130/0.4) provided significantly better lactate clearance and less renal injury than saline in 67 penetrating trauma patients [252]. Because only 42 blunt trauma patients were included in the study, no differences in these parameters could be observed using the different solutions. Therefore, if colloids are administered in patients in whom crystalloids fail to restore target blood pressure, dosing should be within the prescribed limits and, if HES is employed, a modern HES solution should be used.

A number of studies have investigated hypertonic solutions. In 2008, a double-blind RCT in 209 patients with blunt traumatic injuries analysed the effect of treatment with 250 ml 7.5 % hypertonic saline and 6 % dextran 70 compared to lactated Ringer’s solution on organ failure [253]. The intent-to-treat analysis demonstrated no significant difference in organ failure and in ARDS-free survival. However, there was improved ARDS-free survival in the subset (19 % of the population) requiring 10 U or more of packed RBC [253]. A clinical trial with brain injury patients found that hypertonic saline reduced intracranial pressure more effectively than dextran solutions with 20 % mannitol when compared in equimolar dosing [254]. However, Cooper et al. found almost no difference in neurological function 6 months after TBI in patients who had received pre-hospital hypertonic saline resuscitation compared to conventional fluid [255]. Moreover, two large prospective randomised multicentre studies by Bulger and co-workers [256, 257] analysed the effect of out-of-hospital administration of hypertonic fluids on neurological outcome following severe TBI and survival after traumatic hypovolaemic shock. These studies were not able to demonstrate any advantage compared to normal 0.9 % saline among the 2184 patients included. In contrast, a recent study demonstrated that hypertonic solutions interfere with coagulation in this group of patients [258].

In conclusion, the evidence suggests that hypertonic saline solutions are safe, but will neither improve survival nor improve neurological outcome after TBI. So far only one study reported that initial fluid resuscitation with hypertonic saline dextran was beneficial and improved survival compared to normal saline [259].

#### Erythrocytes

##### *Recommendation 17*

**We recommend a target Hb of 7 to 9 g/dl. (Grade 1C)**

#### Rationale

Oxygen delivery to tissues is the product of blood flow and arterial oxygen content, which is directly related to the Hb concentration, therefore decreasing Hb might be expected to give tissue hypoxia. However, compensatory responses to acute normovolaemic anaemia occur, including macro- and microcirculatory changes in blood flow, so the clinical effects of low Hb are complex.

RCTs that have evaluated Hb thresholds for transfusion in critically ill patients have consistently found that restrictive transfusion strategies (Hb thresholds between 7 and 9 g/dL) are as safe as, or safer than, liberal strategies (thresholds ≥9 g/dL) [260–263], with the possible exception of patients following cardiac surgery [264] or with acute coronary syndrome. These studies have excluded patients with massive bleeding. No prospective RCT has compared restrictive and liberal transfusion regimens in trauma patients. A subset of 203 trauma patients from the Transfusion Requirements in Critical Care (TRICC) trial [260] was re-analysed [265]. A restrictive transfusion regimen (Hb transfusion trigger <7.0 g/dl) resulted in fewer transfusions compared with the liberal transfusion regimen (Hb transfusion trigger <10 g/dl) and appeared to be safe. However, no statistically significant benefit in terms of multiple organ failure or post-traumatic infections was observed. It should be emphasised that this study was neither designed nor powered to answer these questions with precision. In addition, it cannot be ruled out that the number of RBC units transfused merely reflects the severity of injury. Nevertheless, RBC transfusions have been shown in multiple studies to be associated with increased mortality [266–270], lung injury [270–272], increased infection rates [273, 274] and renal failure in trauma victims [269].

Because anaemia is a possible cause of secondary ischaemic damage, concerns have been raised about the safety of restrictive transfusion strategies in the subpopulation of patients with TBI. Most early clinical information comes from retrospective observational studies with important methodological limitations. These data have yielded inconsistent results on the effects of RBC transfusion on markers of cerebral perfusion and metabolism in patients with isolated severe TBI. Two systematic reviews published in 2012 stressed the lack of high-level scientific evidence for a specific Hb transfusion trigger in this setting [275, 276]. More recently, two studies have focused on the effect of anaemia and RBC transfusion on neurological outcome after TBI [277, 278]. A retrospective review of data collected prospectively in 1158 patients with a GCS ≤8 in the absence of haemorrhagic shock found that RBC transfusion was associated with worse outcomes (28-day survival, ARDS-free survival, 6-month neurological outcome) when the initial Hb was >10 g/dl [277]. No relationship between RBC transfusion and outcomes was found in patients with an initial Hb ≤10 g/dl [277]. In a 2 × 2 factorial design RCT of 200 patients with TBI at two clinical sites, Robertson et al. compared two Hb transfusion thresholds (7 or 10 g/dl), and separately compared administration of erythropoietin (EPO) or placebo [278]. Patients were enrolled within 6 h of injury and 99 patients were assigned to the 7 g/dl transfusion threshold and 101 patients to the 10 g/dl threshold. The main outcome was neurological recovery at 6 months that was assessed using the Glasgow Outcome Scale dichotomised as favourable or unfavourable. No advantage was found in favour of the 10 g/dl Hb level. In the 7 g/dl threshold group, 42.5 % of patients had a favourable outcome, compared to 33.0 % in the 10 g/dl threshold group (95 % CI for difference −0.06 to 0.25). There was no difference in mortality. More thromboembolic events were observed in the 10 g/dl threshold group [278]. Overall, patients with severe TBI should not be managed with a Hb transfusion threshold different than that of other critically ill patients.

Erythrocytes contribute to haemostasis by influencing the biochemical and functional responsiveness of activated platelets via the rheological effect on platelet margination and by supporting thrombin generation [279]. The effects of the Hct on blood coagulation have not been fully elucidated [280]. An acute reduction of the Hct results in an increase in the bleeding time [281, 282], with restoration upon re-transfusion [281]. This may relate to the presence of the enzyme elastase on the surface of RBC membranes, which may activate coagulation factor IX [283, 284]. However, an animal model showed that a moderate reduction in Hct does not increase blood loss from a standard spleen injury [282], and an isolated in vitro reduction of the Hct did not compromise blood coagulation as assessed by thromboelastometry [285].

Alternative methods of raising Hb have been little studied. The erythropoietic response is blunted in trauma patients [286] and therefore the administration of epoetin alpha appears an attractive option. In a first prospective randomised trial in ICU patients (n = 1302, 48 % being trauma patients) a significant reduction in RBC transfusion percentage from 60.4 to 50.5 % (*P* < 0.001) and reduction in the median number of RBC units transfused from two to one (*P* < 0.001) was observed [287]. In the subgroup of trauma patients 28-day mortality was also reduced [OR 0.43 (0.23 to 0.81)] [287]. In a subsequent prospective randomised trial in ICU patients (n = 1460, 54 % being trauma patients) no significant reduction in RBC transfusions was found [288]. Thrombotic complications were higher in epoetin alpha-treated patients [HR 1.58 (1.09 to 2.28)], however this difference was observed exclusively in patients without heparin prophylaxis [288]. Nevertheless, a trend towards a reduced mortality was found in the entire group of ICU patients, and trauma patients had a lower 29-day [adjusted HR 0.37 (0.19 to 0.72)] and 140-day mortality [adjusted HR 0.40 (0.23 to 0.69)] when treated with epoetin alpha. A third prospective randomised trial enrolled patients (n = 194) with major blunt orthopaedic trauma [289], and no significant effect of epoetin alpha was found, however this study was characterised by a nearly 50 % drop-out rate during the study and a non-significant result is therefore not surprising.

The relatively limited effect of epoetin alpha treatment on transfusion needs may be surprising given the blunted EPO response in trauma patients [286]. However, iron metabolism is also altered after trauma, with iron not being fully available for haematopoiesis [286]. Neither iron metabolism nor availability are fully understood following traumatic injury and complicated by the fact that certain proteins such as ferritin are massively upregulated after trauma as part of the acute-phase response [286]. Intravenous iron may therefore represent another attractive option with which to foster haematopoiesis. Indeed, studies that assess the effect of i.v. iron (with [290, 291] or without [292] concomitant epoetin alpha) showed reduced RBC transfusions [290–292], postoperative infections [290–292], length of hospital stay [291] and mortality in patients with hip fractures [291]. While i.v. iron appears to be promising, oral iron is largely ineffective [293]. In the near future, the Efficacy of Ferric Carboxymaltose With or Without EPO Reducing Red-cell Transfusion Packs in Hip Fracture Perioperative Period (PAHFRAC-01) project, a prospective randomised multicentre study (NCT01154491), will provide further insight into the benefit of i.v. iron and epoetin alpha treatment in patients with hip fracture [294].

In non-trauma patients a meta-analysis showed that preoperative i.v. iron administration was efficacious in correcting preoperative anaemia and in lowering RBC transfusion rates in elective surgery, but found an increased infection rate [295]. This potential risk has not been evaluated for postoperative i.v. iron administration or in trauma patients. Interestingly, i.v. iron treatment in 20,820 haemodialysis patients was associated with a trend towards lower infection rates, lower mortality and a shorter hospital stay [296]. Similarly, i.v. iron treatment equally in anaemic mice with sepsis did not cause increased mortality and corrected anaemia [297]. Short-term preoperative treatment with iron carboxymaltose and epoetin alpha also resulted in a highly significant decrease in postoperative infectious complications (12.0 to 7.9 %) and a shortening of hospitalisation by approximately 1 day in anaemic patients undergoing orthopaedic surgery [291]. In addition, 30-day mortality decreased from 9.4 to 4.8 % in patients with hip fractures [291]. The potential adverse effect of i.v. iron administration in trauma patients may thus be overestimated and certainly remains to be investigated further.

#### Temperature management

##### *Recommendation 18*

**We recommend early application of measures to reduce heat loss and warm the hypothermic patient in order to achieve and maintain normothermia. (Grade 1C)**

#### Rationale

Hypothermia, a core body temperature <35 °C, is associated with acidosis, hypotension and coagulopathy in severely injured patients. The effects of hypothermia include altered platelet function, impaired coagulation factor function (a 1 °C drop in temperature is associated with a 10 % drop in function), enzyme inhibition and fibrinolysis [298–300]. Body temperatures below 34 °C compromise blood coagulation, but this has only been observed when coagulation tests (PT and APTT) are carried out at the low temperatures seen in patients with hypothermia, and not when assessed at 37 °C as is routine practice for such tests.

The profound clinical effects of hypothermia ultimately lead to higher morbidity and mortality [301], and hypothermic patients require more blood products [302]. In a retrospective study of 604 trauma patients who required massive transfusion, a logistic regression analysis demonstrated that a temperature lower than 34 °C was associated with a greater independent risk of mortality of more than 80 % after controlling for differences in shock, coagulopathy, injury severity and transfusion requirements (OR 1.87; 95 % CI 1.18 to 3.0; *P* = 0.007) [303]. A recent study performed a secondary data analysis of 10 years of the Pennsylvania Trauma Outcome Study (PTOS), which analysed 11,033 patients with severe TBI and demonstrated that spontaneous hypothermia at hospital admission was associated with a significant increase in the risk of mortality in patients with severe TBI [304]. Steps to prevent hypothermia and the risk of hypothermia-induced coagulopathy include removing wet clothing, covering the patient to avoid additional heat loss, increasing the ambient temperature, forced air warming, warm fluid therapy, and, in extreme cases, extracorporeal re-warming devices [305–307].

Whereas accidental or induced hypothermia should clearly be avoided in patients without TBI, contradictory results have been reported in patients with TBI. In this trauma setting several large multicentre clinical trials failed to show an effect of therapeutic hypothermia [308–310], while a recent meta-analysis by Crossley et al., which also included several single-centre studies, demonstrated an overall reduction in mortality and poor outcomes [311]. Earlier meta-analyses that examined mortality and neurological outcomes associated with mild hypothermia in TBI were not able to demonstrate such a benefit, which might be explained by the use of different exclusion and inclusion criteria for the analysis [312, 313]. Another reason for controversial results could be differences in the speed of induction and duration of hypothermia, for example it has been shown that 5 days of long-term cooling is more efficacious than 2 days of short-term cooling when mild hypothermia is used to control refractory intracranial hypertension in adults with severe TBI [314, 315]. Moreover, the situation might be different if hypothermia in TBI is compared to conventional treatment that allows fever episodes or compared to strict temperature control between 35.5 and 37 °C [310]. Therefore, at the present time no recommendation can be made in favour of the therapeutic use of whole-body hypothermia in TBI patients.

### IV. Rapid control of bleeding

#### Damage control surgery

##### *Recommendation 19*

**We recommend that damage control surgery be employed in the severely injured patient presenting with deep haemorrhagic shock, signs of ongoing bleeding and coagulopathy. (Grade 1B)**

**Other factors that should trigger a damage control approach are severe coagulopathy, hypothermia, acidosis, inaccessible major anatomic injury, a need for time-consuming procedures or concomitant major injury outside the abdomen. (Grade 1C)**

**We recommend primary definitive surgical management in the haemodynamically stable patient and in the absence of any of the factors above. (Grade 1C)**

#### Rationale

The severely injured patient arriving at the hospital with continuing bleeding or deep haemorrhagic shock generally has a poor chance of survival without early control of bleeding, proper resuscitation and blood transfusion. This is particularly true for patients who present with uncontrolled bleeding due to multiple penetrating injuries or patients with major abdominal injury and unstable pelvic fractures with bleeding from fracture sites and retroperitoneal vessels. The final common pathway in these patients is the exhaustion of physiological reserves with resulting profound acidosis, hypothermia and coagulopathy, also known as the “bloody vicious cycle” or “lethal triad”.

In 1983, Stone et al. described the techniques of abbreviated laparotomy, packing to control haemorrhage and of deferred definitive surgical repair until coagulation has been established [316]. Several articles have since described the beneficial results of this approach, now referred to as “damage control” [317–320]. This approach should be considered in patients with major abdominal injury and a need for adjunctive use of angioembolisation, major abdominal injury and a need to evaluate as early possible other injuries, major abdominal injury and traumatic amputation of a limb. Factors that should trigger a damage control approach in the operating theatre are temperature ≤34 °C, pH ≤7.2, an inaccessible major venous injury, a need for time-consuming procedures in a patient with suboptimal response to resuscitation or inability to achieve haemostasis due to recalcitrant coagulopathy [321, 322].

Damage control surgery of the abdomen consists of three components: the first component is an abbreviated resuscitative laparotomy for control of bleeding, the restitution of blood flow where necessary and the control of contamination. This should be achieved as rapidly as possible without spending unnecessary time on traditional organ repairs that can be deferred to a later phase. The abdomen is packed and temporary abdominal closure is performed. Packing aims to compress liver ruptures or exert direct pressure on the sources of bleeding and abdominal packing may permit further attempts to achieve total haemostasis through angiography and/or correction of the “lethal triad”. The removal of packs should preferably be deferred for at least 48 h to lower the risk of re-bleeding.

The second component of damage control surgery is intensive care treatment, focused on core re-warming, correction of the acid-base imbalance and coagulopathy as well as optimising the ventilation and the haemodynamic status. If complementary angiography and/or further injury investigation is needed, it should be performed during this phase.

The third component is the definitive surgical repair that is performed only when target parameters have been achieved [95, 317–320, 323, 324]. Although the concept of “damage control” intuitively makes sense, no RCTs exist to support it. Retrospective studies support the concept showing reduced morbidity and mortality rates in selective populations [320].

The same “damage control” principles have been applied to orthopaedic injuries in severely injured patients. Scalea et al. were the first to coin the term “damage control orthopaedics” [325]. Relevant fractures are primarily stabilised with external fixators rather than primary definitive osteosynthesis [325–327]. The less traumatic nature and shorter duration of the surgical procedure aims to reduce the secondary procedure-related trauma. Definitive osteosynthesis surgery can be performed after 4–14 days when the patient has recovered sufficiently. Retrospective clinical studies and prospective cohort studies seem to support the concept of damage control. The only available randomised study shows an advantage for this strategy in “borderline” patients [327]. The damage control concept has also been described for thoracic and neurosurgery [328, 329]. In addition to damage control surgical approaches, damage control anaesthesia or resuscitation comprises a number of important measures described in the other recommendations within this document.

#### Pelvic ring closure and stabilisation

##### *Recommendation 20*

**We recommend that patients with pelvic ring disruption in haemorrhagic shock undergo immediate pelvic ring closure and stabilisation. (Grade 1B)**

#### Packing, embolisation and surgery

##### *Recommendation 21*

**We recommend that patients with ongoing haemodynamic instability despite adequate pelvic ring stabilisation receive early pre-peritoneal packing, angiographic embolisation and/or surgical bleeding control. (Grade 1B)**

#### Rationale

The mortality rate for patients with severe pelvic ring disruptions and haemodynamic instability remains high [330, 331]. The early detection of these injuries and initial efforts to reduce disruption and stabilise the pelvis as well as containing bleeding is therefore crucial. Markers of pelvic haemorrhage include anterior-posterior and vertical shear deformations on standard roentgenograms, CT “blush” (active arterial extravasation), bladder compression pressure, pelvic haematoma evident by CT and ongoing haemodynamic instability despite adequate fracture stabilisation [332–334].

The initial therapy for pelvic fractures includes control of venous and/or cancellous bone bleeding by pelvic closure as a first step [335]. Some institutions use primarily external fixators to control haemorrhage from pelvic fractures [332], but pelvic closure may also be achieved using a pelvic binder, a pelvic C-clamp or improvised methods such as a bed sheet [335, 336]. In addition to the pelvic closure, fracture stabilisation and the tamponade effect of the haematoma, pre-, extra or retroperitoneal packing will reduce or stop the venous bleeding [337–339]. Pre-peritoneal packing is used to decrease the need for pelvic embolisation and may be performed simultaneously, or soon after, initial pelvic fracture stabilisation. The most commonly embolised vascular bed and therefore the most studied is the pelvis [340]. Pelvic packing could potentially aid in early intrapelvic bleeding control and provide crucial time for more selective haemorrhage management [337, 339].

Resuscitative endovascular balloon occlusion of the aorta (REBOA) has been used in patients in end-stage shock following blunt and penetrating trauma together with embolisation of the vascular bed in the pelvis. Descriptions of REBOA are few and there are no published trials. Some combined approaches are reported and the technology is evolving [331]. These techniques can be combined with a consecutive laparotomy if deemed necessary [337]. This may decrease the high mortality rate observed in patients with major pelvic injuries who have undergone laparotomy as the primary intervention, however non-therapeutic laparotomy should be avoided [341]. Time to pelvic embolisation for haemodynamically unstable pelvic fractures may affect survival [331, 342].

Angiography and embolisation are currently accepted as highly effective means with which to control arterial bleeding that cannot be controlled by fracture stabilisation [146, 332, 336, 339, 341, 343, 344]. Radiological management can also be usefully applied to abdominal and thoracic bleeding [345–349]. Martinelli et al. [350] report the use of intra-aortic balloon occlusion to reduce bleeding and permit transport to the angiography theatre. In contrast, Morozumi et al. suggest the use of mobile digital subtraction angiography in the emergency department for arterial embolisation performed by trauma surgeons themselves [351]. A number of authors argue that permissive hypotension while obtaining pelvic stabilisation and/or angiography (damage control resuscitation, hypertonic solutions, controlled hypothermia) could achieve better survival. Institutional differences in the capacity to perform timely angiography and embolisation may explain the different treatment algorithms suggested by many authors. Reports on transcatheter angiographic embolisation suggest a 100 % higher mortality during off-hours due to lack of radiological service [352], therefore a multidisciplinary approach to these severe injuries is required.

#### Local haemostatic measures

##### *Recommendation 22*

**We recommend the use of topical haemostatic agents in combination with other surgical measures or with packing for venous or moderate arterial bleeding associated with parenchymal injuries. (Grade 1B)**

#### Rationale

A wide range of local haemostatic agents are currently available for use as adjuncts to traditional surgical techniques to obtain haemorrhagic control. These topical agents can be particularly useful when access to the site of bleeding is difficult. Local haemostatic agents include collagen, gelatin or cellulose-based products, fibrin and synthetic glues or adhesives that can be used for both external and internal bleeding while polysaccharide-based and inorganic haemostatics are still mainly used and approved for external bleeding.

The use of topical haemostatic agents should consider several factors such as the type of surgical procedure, cost, severity of bleeding, coagulation status and each agent’s specific characteristics. Some of these agents should be avoided when autotransfusion is applied, and several other contraindications need to be considered [353, 354]. The capacity of each agent to control bleeding was initially studied in animals, but increasing experience in humans is now available [353–369].

The different types of local haemostatic agents are briefly presented according to their basis and haemostatic capacity.

- Collagen-based agents trigger platelet aggregation, resulting in clot formation when in contact with a bleeding surface. They are often combined with a procoagulant substance such as thrombin to enhance the haemostatic effect. A positive haemostatic effect has been shown in several human studies [360–363].
- Gelatin-based products can be used alone or in combination with a procoagulant substance [353]. Swelling of the gelatin in contact with blood reduces the blood flow and, in combination with a thrombin-based component, enhances haemostasis [357–359]. The products have been successfully used for local bleeding control in brain or thyroid surgery when electrocautery may cause damage to nerves [356] or to control bleeding from irregular surfaces such as post-sinus surgery [355].
- Absorbable cellulose-based haemostatic agents have been widely used to treat bleeding for many years, and case reports as well as a prospective observational human study support their effectiveness [368]. The oxidised cellulose-based product can be impregnated with polyethylene glycol and other salts and achieve comparable and more rapid haemostasis compared to the combined products described below [367].
- Fibrin and synthetic glues or adhesives have both haemostatic and sealant properties, and their significant effect on haemostasis has been shown in several randomised controlled human studies involving vascular, bone, skin and visceral surgery [364–366].
- Polysaccharide-based haemostatics can be divided into two broad categories [353]: N-acetyl-glucosamine-containing glycosaminoglycans purified from microalgae and diatoms and microporous polysaccharide haemospheres produced from potato starch. The mechanism of action is complex and depends on the purity or combination with other substances such as cellulose or fibrin. A number of different products in the form of pads, patches or bandages are currently available and have been shown to be efficient for external use and for splanchnic bleeding in animals. An observational study showed that haemorrhage control was achieved using a poly-*N*-acetyl glucosamine-based bandage applied to ten patients with severe hepatic and abdominal injuries, acidosis and clinical coagulopathy [369].

Although the evidence is mainly observational, these agents have become widely used.

### V. Initial management of bleeding and coagulopathy

#### Coagulation support

##### *Recommendation 23*

**We recommend that monitoring and measures to support coagulation be initiated immediately upon hospital admission. (Grade 1B)**

#### Rationale

Some means with which to evaluate trauma-related coagulopathy have been developed [370], however, these largely confirm the main pathophysiological mechanisms described above [371, 372]. While several general pathophysiological mechanisms can be described that result in trauma-related coagulopathy, it is essential to quickly determine the type and degree of coagulopathy in the individual patient in order to determine the most prominent cause or causes to be treated specifically in a goal-directed manner [373].

Early monitoring of coagulation is essential to detect trauma-induced coagulopathy and to define the main causes, including hyperfibrinolysis [13, 25, 179, 183, 374]. Early therapeutic intervention does improve coagulation tests [375], reduce the need for transfusion of RBC, FFP and platelets [12, 376], reduce the incidence of post-traumatic multi-organ failure, shorten length of hospital stay [12] and may improve survival [377, 378]. Interestingly, the success of early algorithm-based and goal-directed coagulation management in reducing transfusions and improving outcome, including mortality, has also been shown in cardiac surgery [202, 379–381]. Therefore, early algorithm-based and goal-directed coagulation management treatment is likely to improve the outcome of severely injured patients [382, 383]. This has indeed been shown in a prospective randomised study [384] and in a large study assessing the introduction of such a concept in two large Italian trauma centres [385]. However, there are also studies in which no survival benefit could be shown [375, 386, 387]; variation in published results may be due to choice of coagulation monitoring tests (negative trials tended to use traditional laboratory values such as PT, APTT and platelet count) and type of therapy used (negative trials tended to use only FFP and platelets [379–381, 384].

#### Initial coagulation resuscitation

##### *Recommendation 24*

**In the initial management of patients with expected massive haemorrhage, we recommend one of the two following strategies:**

- **Plasma (FFP or pathogen-inactivated plasma) in a plasma**–**RBC ratio of at least 1:2 as needed. (Grade 1B)**
- **Fibrinogen concentrate and RBC according to Hb level. (Grade 1C)**

#### Rationale

We define “initial resuscitation” as the period between arrival in the emergency department and availability of results from coagulation monitoring (coagulation screen, fibrinogen level and/or viscoelastic monitoring and platelet count). There are still conflicting opinions about use of plasma as the initial strategy to support coagulation, and several authors, mainly in Europe, strongly disagree with the initial transfusion of patients based on an empirical ratio rather than guided by concurrent laboratory data (goal-directed therapy) [388]. In the absence of rapid point-of-care coagulation testing to facilitate goal-directed therapy, initial treatment with blood components in a fixed ratio may constitute a reasonable approach. If concurrent coagulation results are available, they should be used to guide therapy.

In May 2005, based on reports from the ongoing conflict in Iraq, an international expert conference on massive transfusion hosted by the US Army’s Institute of Surgical Research introduced a new concept for the resuscitation of patients with massive bleeding and recommended the immediate administration of coagulation components with a 1:1:1 ratio for RBC, plasma and platelets [389–391] until laboratory measurements to adjust therapy were available. In the following few years retrospective evidence from both military and civilian practice suggested improved outcomes in patients with massive bleeding after the adoption of a massive transfusion protocol, including the early administration of high-dose plasma therapy [392]. Several subsequent studies focused on this strategy to determine whether standard doses of plasma and platelets in a fixed ratio relative to RBC were able to improve survival. Notwithstanding a large number of studies, the evidence with respect to the use of high ratios shows conflicting results. Although many authors suggested that early and aggressive plasma transfusion may reduce mortality [393], the optimal FFP:RBC and platelet:RBC ratio was controversial because of the possible survival bias that flaws most studies [394, 395]. Survival bias is the bias resulting from the fact that surviving patients are more likely to receive more plasma and platelets compared with non-survivors, because they live long enough to receive those blood products. A prospective multicentre study that included a large population of patients undergoing massive transfusion showed that high FFP:RBC and platelet:RBC ratios are associated with a survival benefit, also when time-dependency is accounted for [225], however other authors have come to opposite conclusions [396]. Khan et al. were unable to confirm significant increases in procoagulant factor levels or consistent correction of any measure of clot function when FFP was delivered during the acute phase of ongoing bleeding [396]. The recent Pragmatic, Randomized Optimal Platelet and Plasma Ratios (PROPPR) randomised clinical trial in 680 trauma patients who were suspected to sustain or had experienced massive blood loss [397, 398] reported that there was no difference in overall survival between early administration of plasma, platelets and RBC in a 1:1:1 ratio (FFP:platelets: RBC) compared to 1:1:2. However more patients in the 1:1:1 group achieved “anatomic” haemostasis and fewer experienced death due to exsanguination by 24 h. The early use of platelets and high level of FFP use in the 1:1:1 group was not associated with a significantly increased rate of complications. The early administration of platelets as described in Recommendation 29 is important, however from a practical standpoint platelets may not be readily available during the initial resuscitation period described here.

As with all products derived from human blood, the complications associated with FFP treatment include circulatory overload, ABO incompatibility, transmission of infectious diseases (including prion diseases) and mild allergic reactions. Transfusion-related acute lung injury (TRALI) [399, 400] is a severe adverse effect associated with the presence of leucocyte antibodies in transfused plasma. The risk of TRALI has been greatly reduced by avoiding the use of plasma from women with a history of pregnancy [401]. Transmission of infectious diseases can be minimised by the use of pathogen-inactivated plasma (industrial purified plasma).

Further controversy concerns the use of plasma to correct the decreased fibrinogen levels associated with haemorrhagic shock. Haemostasis is critically dependent on fibrinogen as a substrate for clot formation and the ligand for platelet aggregation. Fibrinogen is the single coagulation factor that is affected more and earlier in association with trauma-induced coagulopathy. Many bleeding trauma patients with trauma-induced coagulopathy present with a fibrinogen depletion, below levels currently recommended for therapeutic supplementation. Recently Schlimp et al. [159] demonstrated that levels of fibrinogen lower than 1.5 g/l are detected in as many as 73 % of patients with an admission Hb lower than 100 g/l and in 63 % of those with a BE lower than -6. Moreover, Rourke et al. [402] found low fibrinogen in 41 % of the patients who were hypotensive on admission. In this study, hypotension, increasing shock severity and a high degree of injury (ISS ≥25), were all associated with a reduction in fibrinogen levels. Fibrinogen depletion is associated with poor outcomes and survival improves with administered fibrinogen [403]. Fibrinogen is by far the coagulation protein with the highest plasma concentration. One litre of plasma contains on average 2 g of fibrinogen. Therefore for very initial coagulation support, while waiting for the results of viscoelastic or laboratory tests, it has been proposed to administer 2 g of fibrinogen to mimic the expected 1:1 ratio corresponding to the first four units of RBC and potentially correct hypofibrinogenaemia if already present [385, 404]. Recent experimental data show that administration of fibrinogen does not suppress endogenous fibrinogen synthesis [405].

Administration of plasma to bleeding patients may stabilise fibrinogen levels, avoiding a further decrease, but plasma transfusions cannot contribute to a significant increase in fibrinogen level unless very high volumes are infused [406]. The Activation of Coagulation and Inflammation in Trauma (ACIT) study [396] confirmed these findings, showing that the percentage of coagulopathic patients increased with a standard near 1:1 FFP:RBC transfusion protocol. Similar results were recently reported by Khan et al. [15]. Again, a 1:1 FFP:RBC transfusion protocol did not alleviate coagulopathy; the percentage of coagulopathic patients even increased the longer this treatment lasted. Interestingly, in the same study it was shown that only high-dose fibrinogen administration resulted in improved coagulation and a reduction in coagulopathy. Furthermore, both FFP and pathogen-inactivated plasma need to be group-matched, thawed and warmed prior to administration. Therefore, unless pre-thawed plasma is available, plasma transfusion cannot be initiated at the same time as universal RBC transfusion. An average delay of 93 min was reported by Snyder et al. [394] and recently confirmed by Halmin et al. [407], possibly explaining why a real-life targeted plasma:RBC ratio is achieved only a few hours after treatment initiation. During this interval the fibrinogen level is likely to be lower than desired.

#### Antifibrinolytic agents

##### *Recommendation 25*

**We recommend that tranexamic acid be administered as early as possible to the trauma patient who is bleeding or at risk of significant haemorrhage at a loading dose of 1 g infused over 10 min, followed by an i.v. infusion of 1 g over 8 h. (Grade 1A)**

**We recommend that tranexamic acid be administered to the bleeding trauma patient within 3 h after injury. (Grade 1B)**

**We suggest that protocols for the management of bleeding patients consider administration of the first dose of tranexamic acid en route to the hospital. (Grade 2C)**

#### Rationale

Tranexamic acid (trans-4-aminomethyl cyclohexane-1-carboxylic acid; TXA) is a synthetic lysine analogue that is a competitive inhibitor of plasminogen. TXA is distributed throughout all tissues, and the plasma half-life is 120 min [408]. The Clinical Randomisation of Antifibrinolytic therapy in Significant Haemorrhage (CRASH-2) trial [409] assessed the effects of early administration of a short course of TXA on death, vascular occlusive events and the receipt of blood product transfusion in trauma patients who were bleeding or at risk of significant bleeding. The trial randomised 20,211 adult trauma patients with or at risk of significant bleeding to either TXA (loading dose 1 g over 10 min followed by infusion of 1 g over 8 h) or matching placebo within 8 h of injury. The primary outcome was death in hospital within 4 weeks of injury. All analyses assessed the intention-to-treat population. All-cause mortality was significantly reduced with TXA by 1.5 %, and the risk of death due to bleeding was significantly reduced by 0.8 % and a reduction in bleeding deaths by one-third, mainly through preventing exsanguination within the first 24 h [410, 411]. One retrospective study has suggested that TXA is of no benefit in patients with viscoelastic hyperfibrinolysis [412] and another found TXA to reduce multiple organ failure and mortality in severely injured shocked patients [413]. This discrepancy is probably attributable to methodological limitations.

The risk of precipitated thrombosis with the use of the lysine analogues TXA and ε-aminocaproic acid had been of major theoretical concern; however CRASH-2 showed that the rate of venous thromboembolism (VTE) was not altered, while post-traumatic arterial thromboses, especially myocardial infarction, were lower with the use of TXA. No adverse events were described with the use of TXA in CRASH-2, although an increased rate of seizures has been described in patients receiving a high-dose TXA undergoing cardiac surgery [414], probably reflecting the role of fibrinolytic molecules as neurotransmitters.

An unplanned subgroup analysis of the CRASH-2 data [415] showed that early treatment (≤1 h from injury) significantly reduced the risk of death due to bleeding by 2.5 %. Treatment administered between 1 and 3 h also reduced the risk of death due to bleeding by 1.3 %. Treatment given after 3 h increased the risk of death due to bleeding by 1.3 %; therefore we recommend that TXA not be given more than 3 h following injury. In order to ensure that TXA is given early, the administration of TXA at the pre-hospital site of injury needs to be planned, and we suggest that protocols for the management of bleeding patients consider administration of the first dose of TXA at the site of injury. If TXA is restricted to massive transfusion protocols or only used in patients clinically judged to be at “high risk”, it is estimated that only 40 % of the potential benefit from this treatment will be achieved [416]. For the full benefit, TXA should therefore be administered to all patients with trauma and significant bleeding. Thus TXA should be included as part of each institutional “trauma management protocol” not the “massive blood loss” or “major haemorrhage” protocols.

The cost-effectiveness of TXA in trauma has been calculated in three countries [417, 418]: Tanzania as an example of a low-income country, India as a middle-income country and the UK as a high-income country. The cost of TXA administration to 1000 patients was US$17,483 in Tanzania, US$19,550 in India and US$30,830 in the UK. The estimated incremental cost per life year gained of administering TXA was $48, $66 and $64 in Tanzania, India and the UK respectively.

ε-aminocaproic acid is also a synthetic lysine analogue that has a potency tenfold weaker than that of TXA. It is administered at a loading dose of 150 mg/kg followed by a continuous infusion of 15 mg/kg/h. The initial elimination half-life is 60–75 min and must therefore be administered by continuous infusion in order to maintain therapeutic drug levels until the bleeding risk has diminished. This agent is a potential alternative to TXA if TXA is not available.

Due to concerns about safety [419] the use of aprotinin is not advised in bleeding trauma patients, now that TXA has been shown to be efficacious and safe.

### VI. Further resuscitation

#### Goal-directed therapy

##### *Recommendation 26*

**We recommend that resuscitation measures be continued using a goal-directed strategy guided by standard laboratory coagulation values and/or viscoelastic tests. (Grade 1C)**

#### Rationale

Treatment of the bleeding trauma patient is carried out in a manner that supports the concept that normalisation of coagulation parameters will improve outcomes, although there is little evidence for or against this presumption. During initial resuscitation the state of the coagulation system is unknown until test results are available, therefore blood, blood products and other treatment is administered using a “best guess” policy, with local variation as there is no firm evidence for the best “formula” to follow. The “best guess” policy usually comprises a specified ratio of RBC, FFP and other treatments, given in “bundles” or “packs”. During further resuscitation as more information becomes available from laboratory or point-of-care tests, the treatments being administered are modified and management switches to becoming goal-directed. If no information is available initially, it is reasonable to presume that the severely injured patient is coagulopathic and initiate “best guess” treatment. During further resuscitation, a goal-directed approach is appropriate.

Clinicians need to be aware of the time lag between a sample being taken and the result being available, but should not delay treatment while waiting for a result. Delays in coagulation results represent a much greater challenge in the absence of point-of-care testing. Lack of awareness of the dynamic status of the patient’s condition can lead to treatment that is always “behind the curve”. To avoid this hazard, patient treatment should be determined by a combination of the test results and the clinician’s judgement about how the patient’s coagulation status may have changed since the test was taken. The specific goals for treatment are explored in the following sections.

#### Fresh frozen plasma

##### *Recommendation 27*

**If a plasma-based coagulation resuscitation strategy is used, we recommend that plasma (FFP or pathogen-inactivated plasma) be administered to maintain PT and APTT <1.5 times the normal control. (Grade 1C)**

**We recommend that plasma transfusion be avoided in patients without substantial bleeding. (Grade 1B)**

#### Rationale

Plasma (thawed FFP or pathogen-inactivated plasma) is used for many years and throughout the world as a source of coagulation factors. FFP contains about 70 % of the normal level of all clotting factors; therefore, it would seem to be an adequate source for replacement; however, different preparations show great variability [256]. We recommend the use of FFP if a plasma-based coagulation strategy is applied and there is evidence of coagulation factor deficiency as evidenced by a prolonged PT and APTT greater than 1.5 times the normal control or viscoelastic measures. RCTs that investigate the utility of this approach have never been conducted, however this strategy is widely applied. Management of haemorrhage should be carefully monitored to ensure that FFP transfusion is appropriate, as it is associated with significant risks, including circulatory overload, allergic reactions and TRALI.

A prolongation of “clotting time” or “reaction time” using viscoelastic tests may also be considered an indication for the administration of FFP, however the scientific evidence for this is scarce and a normalisation of fibrinogen level as described in recommendation 28 will often normalise these parameters.

#### Fibrinogen and cryoprecipitate

##### *Recommendation 28*

**If a concentrate-based strategy is used, we recommend treatment with fibrinogen concentrate or cryoprecipitate if significant bleeding is accompanied by viscoelastic signs of a functional fibrinogen deficit or a plasma fibrinogen level of less than 1.5**–**2.0 g/l. (Grade 1C)**

**We suggest an initial fibrinogen supplementation of 3**–**4 g. This is equivalent to 15**–**20 single donor units of cryoprecipitate or 3**–**4 g fibrinogen concentrate. Repeat doses must be guided by viscoelastic monitoring and laboratory assessment of fibrinogen levels. (Grade 2C)**

#### Rationale

Fibrinogen is the final component in the coagulation cascade, the ligand for platelet aggregation and therefore key to effective coagulation and platelet function [280, 420]. Hypofibrinogenaemia is a common component of complex coagulopathies associated with massive bleeding. Fibrinogen levels decrease early in many patients who sustain severe trauma, and low fibrinogen levels are associated with higher transfusion requirements and increased mortality [421]. Since there are no fibrinogen reserves outside the plasma, the overall stock of fibrinogen within the body amounts to just 10 g in a 80 kg individual, which means that a sharp fall in fibrinogen level cannot be quickly compensated. Recently, Schlimp et al. [159] demonstrated that fibrinogen levels on admission show strong correlation with rapidly obtainable routine laboratory parameters such as Hb and base excess. Fibrinogen levels lower than 1.5 g/l are detected in as many as 73 % of trauma patients with an admission Hb lower than 10 g/dl and in 63 % of those with a BE lower than -6. Moreover Rourke et al. [402] observed low fibrinogen levels in 41 % of the patients who were hypotensive on admission.

Coagulopathic civilian trauma patients had a median fibrinogen concentration of 0.9 g/l [interquartile ratio (IQR) 0.5–1.5 g/l] in conjunction with a maximum fibrinogen thromboelastometric maximum clot firmness (MCF) of 6 mm (IQR 0–9 mm) using thromboelastometry, whereas only 2.5 % of healthy volunteers had a MCF of <7 mm [25]. In trauma patients, a MCF of 7 mm was associated with a fibrinogen level of approximately 1.5–2.0 g/l [191]. During postpartum haemorrhage, fibrinogen plasma concentration is the only coagulation parameter independently associated with progress towards severe bleeding, with a level <2 g/l having a positive predictive value of 100 % [422].

An early observational study suggested that fibrinogen substitution can improve survival in combat-related trauma [403]. In the civilian setting, the use of thromboelastometry-guided fibrinogen replacement reduced the exposure to allogeneic blood products [12, 378, 385]. Retrospective reviews of single-centre experiences managing massive blood loss in trauma patients have also suggested a reduced mortality when compared to expected mortality [378] and increased 30-day survival [423]. However, there are still no adequately powered prospective clinical trials to demonstrate the risk:benefit of using a source of additional fibrinogen to manage bleeding trauma patients [424, 425]. It has been suggested that the required fibrinogen dosage may be estimated based on the results of thromboelastometric monitoring using a simple formula: the administration of 0.5 g fibrinogen to 80 kg patient may increase the A10 MCF by 1 mm, the application of which may facilitate a rapid and predictable increase in plasma fibrinogen to a target level [426].

The retrospective Military Application of Tranexamic Acid in Trauma Emergency Resuscitation (MATTERs II) study of massive military bleeding suggested that cryoprecipitate may independently add to the survival benefit of TXA in the seriously injured patient who requires transfusion [427]. However, cryoprecipitate is often administered with great delay: in the Prospective, Observational, Multicenter, Major Trauma Transfusion (PROMMTT) study [428] the median time from admission to the first cryoprecipitate unit was 2.8 h (IQR 1.7–4.5) and in the ACIT study [396], cryoprecipitate was administered only after the first six units of blood. A small randomised, controlled feasibility trial suggested that the early administration of cryoprecipitate in trauma patients is possible [429].

Methodological issues associated with the various techniques with which to measure fibrinogen concentration remain [430, 431]. The Clauss method is the most frequently recommended laboratory method, however in the presence of artificial colloids such as HES this method may overestimate the actual fibrinogen concentration, but remains the gold standard as it measures fibrinogen function directly [431]. Fibrinogen thromboelastometry is also influenced by Hct [432] and factor XIII levels [433].

The issue of whether the administration of fibrinogen via factor concentrate, cryoprecipitate or FFP is associated with an increased risk of hospital-acquired VTE has never been systematically addressed. However, fibrinogen levels are expected to rise as part of the acute phase response after major surgery and trauma [371, 434–436] even without intraoperative fibrinogen administration. Interestingly, intraoperative administration of fibrinogen concentrate in trauma patients [371] or in patients undergoing cardiac surgery resulted in higher intra- and early postoperative fibrinogen levels but fibrinogen levels were identical on postoperative days 1–7 in patients with and without intraoperative fibrinogen administration [436, 437].

The rationale for fibrinogen administration should be read in conjunction with that for plasma (Recommendation 27). There is insufficient evidence to support a firm statement about which of the two strategies is best, or if even a combined used of both strategies could be of benefit.

#### Platelets

##### *Recommendation 29*

**We recommend that platelets be administered to maintain a platelet count above 50 × 10**^**9**^**/l. (Grade 1C)**

**We suggest maintenance of a platelet count above 100 × 10**^**9**^**/l in patients with ongoing bleeding and/or TBI. (Grade 2C)**

**If administered, we suggest an initial dose of four to eight single platelet units or one aphaeresis pack. (Grade 2C)**

#### Rationale

Although platelets play a pivotal role in haemostasis after injury, the effect of platelet transfusion is controversial. Historically, platelet transfusion was based on critical thresholds of platelet counts. One small prospective study performed in massively transfused patients found a platelet count of <100 × 10^9^/l as the threshold for diffuse bleeding [438], and another study indicated a platelet count <50 × 10^9^/l or fibrinogen <0.5 g/l as the most sensitive laboratory predictors of microvascular bleeding [439]. However, an older prospective randomised trial evaluating prophylactic platelet transfusion at a ratio to whole blood of 1:2 versus same amount of plasma in patients receiving ≥12 units of whole blood in 12 h concluded that platelet administration did not affect microvascular non-surgical bleeding [440]. Recently, it was shown that a low or decreasing platelet count in trauma patients predicts greater mortality [441] and proactive administration of platelets in patients with massive bleeding due to ruptured aortic abdominal aneurysms increased survival from 30 to 45 % when the platelet count was >50 × 10^9^/l as compared to <50 × 10^9^/l and further increased to 69 % for those with platelet count >100 × 10^9^/l [442].

A lower than normal platelet count also predicts progression of intracranial haemorrhage (ICH) and mortality after TBI [443, 444]. In patients with blunt TBI, a platelet count of ≤100 × 10^9^/l was found to be an independent predictor of ICH progression using repeated head CT, need for neurosurgical intervention and mortality [445]. However, platelet transfusion did not influence the outcome in patients with TBI and moderate thrombocytopenia (50–107 × 10^9^/l) [446]. Accordingly, at this time there is weak scientific evidence to support a particular platelet count threshold for platelet transfusion in the trauma patient.

The normal therapeutic dose of platelets is one concentrate (60–80 × 10^9^ platelets) per 10 kg body weight. One aphaeresis platelet product, which is approximately equivalent to six whole blood-derived units, generally contains approximately 3–4 × 10^11^ platelets in 150–450 ml donor plasma [447, 448], depending on local collection practice. The platelet-rich plasma used in the United States contains fewer platelets than the high-output platelet concentrate manufactured by apheresis or pooling five buffy coats mainly used in Europe [449]. This difference should be considered when analysing the results of studies supporting higher levels of platelet transfusion. A dose of four to eight platelet units or a single-donor aphaeresis unit is usually sufficient to provide haemostasis in a thrombocytopenic, bleeding patient and should increase the platelet count by 30–50 × 10^9^/l [375]. However, the usual 60–70 % recovery rate in peripheral blood may be lower under conditions associated with increased platelet consumption [449]. The platelets transfused must be ABO-identical, or at least ABO-compatible, in order to provide a good yield [448].

Early, up-front administration of platelets in patients with massive bleeding who are not yet thrombocytopenic is controversial. In initial acute loss, the bone marrow and spleen variably release platelets into the circulation, and therefore their decrease in the peripheral blood is delayed. As a result, platelet counts are typically within normal range (150 × 10^9^/l to 400 × 10^9^/l) during early traumatic coagulopathy [441, 450–452]. Upon admission, platelet count <150 × 10^9^/l has been reported in only 4 % of trauma patients with an ISS of 5 and in 18 % of patients with ISS >5 [450]. In another study, less than 5 % of patients arrived in the emergency room with a platelet count <100 × 10^9^/l [11]. In a large cohort study over an 8.5 year period, platelet counts decreased markedly in the 2 h after hospital admission and 1 × 10^9^/l/h over the next 22 h, suggesting an important role for the treatment administered [441]. A platelet count of 50 × 10^9^/l may be anticipated when approximately two blood volumes have been replaced by fluid or red cell components [421].

Platelet count on admission, may be predictive of outcome as documented in some cohorts of massively transfused trauma patients, in which platelet count was inversely correlated with injury severity [450], morbidity [443] and mortality [450, 451, 453]. The association between lower platelet counts and higher mortality applies to platelet counts well into the normal range [441, 451], suggesting that a normal platelet count may be insufficient for cellular-based haemostasis after severe trauma. Thus, platelet count alone is a weak indicator of platelet transfusion need because it ignores platelet function.

There is a growing body of evidence to support a prominent role for platelet dysfunction in the pathophysiology of traumatic coagulopathy [454, 455], and it seems that moderate or even mildly decreased platelet aggregation is strongly associated with mortality [214, 456, 457]. Recently, it was found that platelet dysfunction (analysed by thromboelastographic platelet mapping) is present after injury even before substantial fluid or blood products have been administered and continues during the resuscitation period, suggesting a potential role for early platelet transfusion in the management of traumatic coagulopathy [455]. In a retrospective cohort analysis of patients with TBI, it was possible to reverse aspirin-like platelet inhibition in 42 % of patients using platelet transfusion [458], while in a prospective study performed in patients with isolated TBI, platelet dysfunction involved the response to collagen and was not improved by the administration of platelets [459].

There is still no high-quality evidence to support up-front platelet transfusion or higher doses of platelets given in pre-defined ratios with other blood products in trauma patients. Although most of the combat [460, 461] and civilian studies [462–466], one meta-analysis [467] and one systematic review [468] that investigated the impact of platelet transfusion in severe trauma and massive transfusion showed an improved survival rate among patients receiving high platelet:RBC ratios, such evidence provided by retrospective and observational studies may be subject to serious confounding factors, such as survivorship bias [467] or co-interventions [469]. The timing of platelet transfusion relative to the initiation of RBC and FFP transfusion was not reported in most of the studies, and there may be more than one optimal ratio depending on trauma severity, degree and dynamics of blood loss and previous fluid administration [467]. Another major drawback to these observational studies is the wide range of platelet:RBC ratios examined, along with reported poor compliance with specified platelet ratios during active resuscitation [470]. Moreover, the actual number of platelets transfused to each patient is unknown because blood bank standards estimate only the minimum number of platelets contained in apheresis and pooled platelet units [468]. However, recent large prospective cohort studies showed that a high platelet:RBC ratio was associated with survival benefit as early as 6 h after admission, suggesting that survivor bias is unlikely [469, 471]. Interestingly, in one study the significant protective association between higher platelet ratios and mortality was concentrated during the first 6 h only, in contrast to high plasma ratios which were protective throughout the first 24 h [471].

Negative [472–474] and partially positive results [475] with high platelet:RBC ratios were also reported in patients receiving massive transfusion. Interestingly, patients with penetrating injuries [472] and females [475] do not benefit from high platelet:RBC ratios, and no difference in mortality was observed in patients with non-massive transfusion receiving higher platelet:RBC ratios [476]. When a research intervention (before-and-after introduction of a massive haemorrhage protocol performed with high plasma and platelet:RBC ratios) was reported, improved survival was shown in three studies [180, 392, 423], but not in a further study [477].

A small feasibility RCT that included trauma patients expected to require a massive transfusion compared a fixed ratio of RBC, FFP and platelets in a 1:1:1 ratio to standard practice (laboratory result-guided transfusion protocol). Nascimento et al. found an all-cause 28-day mortality of 32 % in the 1:1:1 group vs. 14 % in the laboratory result-guided transfusion protocol group (RR for fixed ratio 2.27; 95 % CI 0.98 to 9.63, *P* = 0.053) [384]. However, this study was not powered to detect a difference in mortality and the 1:1:1 ratio was achieved in only 57 % of the fixed ratio group.

One additional reason for the lack of clarity in these studies is the difficulty in separating the effect of a high platelet:RBC ratio from the effect of a high plasma:RBC ratio. Patients receiving a combination of high plasma and high platelet ratios had an improved 6-h, [463, 464, 469], 24-h [392, 460, 463, 465, 466, 469], 30-day [180, 392, 423, 460, 462, 463, 466], in-hospital [464] and discharge survival [465]. However, in comparison with increased plasma:RBC ratios, the impact exerted by platelets on survival was not as strong [472, 475], higher than the impact of plasma [423] or even absent [473]. In contrast to the civilian studies, US military experience with blood transfusions demonstrated that higher platelet ratios are independently associated with increased survival [478] and that the association was stronger for high platelet ratios than for high FFP ratios [461]. In patients with TBI, transfusion of a high platelet:RBC ratio and not a high plasma:RBC ratio was found to be associated with improved survival [479].

Early (within minutes of arrival to a trauma centre) administration of plasma, platelets and RBC is also supported by the first RCT designed to evaluate the benefit of blood product ratios (1:1:1 or 1:1:2 FFP:platelets:RBC) on patient outcome [397]. More patients in the 1:1:1 group achieved haemostasis and fewer experienced death as a result of exsanguination at 24 h. However, a 1:1:1 ratio compared to a 1:1:2 ratio did not result in significant differences in all-cause mortality at 24 h or 30 days [397]. Unfortunately, this study did not independently examine the effects of plasma and platelets on outcomes.

A theoretical shortcoming of ratio-driven resuscitation is over-transfusion with plasma and platelets, resulting in no benefit or in added morbidity such as multiple organ failure [466, 480]. Recent observations suggest that both early FFP (0–6 h) and delayed (7–24 h) platelet transfusions are risk factors for hypoxaemia and ARDS after 24 h, respectively [481]. The age of transfused platelets may also play a role [482]. Although decreased morbidity due to aggressive use of plasma and platelets has been reported [382, 463, 464], evidence for routine early prophylactic platelet transfusion as part of a massive transfusion protocol is weak [483].

#### Calcium

##### *Recommendation 30*

**We recommend that ionised calcium levels be monitored and maintained within the normal range during massive transfusion. (Grade 1C)**

#### Rationale

Acute hypocalcaemia is a common complication of massive transfusion [484]. Citrate added to stored blood binds calcium and may reduce the serum level of the ionised fraction [485]. Two observational cohort studies showed that low ionised calcium levels at admission are associated with increased mortality as well as an increased need for massive transfusion [486, 487]. Hypocalcaemia during the first 24 h can predict mortality and the need for multiple transfusion better than the lowest fibrinogen concentrations, acidosis and the lowest platelet counts [486]. Measurement of ionised calcium levels at admission may facilitate the rapid identification of patients who require massive transfusion, allowing for earlier preparation and administration of appropriate blood products. However, no data are available to demonstrate that the prevention of ionised hypocalcaemia reduces mortality among patients with critical bleeding who require massive transfusion.

Calcium in the extracellular plasma exists either in a free ionised state (45 %) or bound to proteins and other molecules in a biologically inactive state (55 %). The normal concentration of the ionised form ranges from 1.1 to 1.3 mmol/l and is influenced by the pH; a 0.1 unit increase in pH decreases the ionised calcium concentration by approximately 0.05 mmol/l [488]. The availability of ionised calcium is essential for the timely formation and stabilisation of fibrin polymerisation sites, and a decrease in cytosolic calcium concentration precipitates a decrease in all platelet-related activities [489]. In addition, contractility of the heart and systemic vascular resistance are low at reduced ionised calcium levels. Combining beneficial cardiovascular and coagulation effects, the level of ionised calcium concentration should therefore be maintained within the normal range [489].

Early hypocalcaemia following traumatic injury shows a significant correlation with the amount of FFP transfused and also with the amount of infused colloids, but not with crystalloids. Hypocalcaemia is most common in association with FFP and platelet transfusion because these products contain high citrate concentrations. Citrate undergoes rapid hepatic metabolism, and hypocalcaemia is generally transient during standard transfusion procedures. Citrate metabolism may be dramatically impaired by hypoperfusion states, hypothermia and in patients with hepatic insufficiency [489].

#### Antiplatelet agents

##### *Recommendation 31*

**We suggest administration of platelets in patients with substantial bleeding or intracranial haemorrhage who have been treated with antiplatelet agents. (Grade 2C)**

**We suggest the measurement of platelet function in patients treated or suspected of being treated with antiplatelet agents. (Grade 2C)**

**We suggest treatment with platelet concentrates if platelet dysfunction is documented in a patient with continued microvascular bleeding. (Grade 2C)**

#### Rationale

Conflicting data exist about the effects of antiplatelet agents (APA), mainly aspirin and clopidogrel, on traumatic bleeding. Data from non-elective orthopaedic procedures show either increased perioperative blood loss in patients taking APA prior to surgery [490, 491] or no effect [492–494]. The need for blood transfusion in orthopaedic patients on APA is also controversial, being either higher [491, 495, 496] or similar to control patients [492–494, 497, 498]. Pre-injury use of APA did not affect morbidity and mortality in retrospective studies of patients with pelvic fractures [495] or general trauma without brain injury [499], but had conflicting effects on early hip fracture surgery outcome [491, 494, 497, 498, 500]. Aspirin was associated with a significantly increased need for postoperative blood transfusion (adjusted OR 1.8; 95 % CI 1.04 to 3.3) and significantly higher all-cause mortality (adjusted HR 2.35; 95 % CI 1.23 to 4.49) during 1 year after hip fracture surgery in one observational cohort study [491]. However, retrospective studies have shown that postoperative outcomes of hip fracture surgery in patients on clopidogrel were similar to those not taking the agent at the time of surgery performed within 48 h [497, 498, 500, 501], except for a significantly longer hospital stays in some studies [494, 498].

The role of pre-injury APA in the genesis of ICH in patients with blunt head trauma is controversial as well [502–506]. One observational study found a fivefold increase in traumatic ICH in patients on APA [502]. Even mild head trauma (GCS 14–15) while on APA was associated with a high incidence of ICH [507–509], mandating a longer period of observation for delayed ICH in this group of patients [510, 511]. Others failed to demonstrate the association [503, 504, 506], however, pre-injury use of clopidogrel was significantly associated with ICH following minor trauma (OR 16.7; 95 % CI 1.71 to 162.7) [512].

The relationship between outcome and pre-injury APA in the setting of ICH is conflicting in both the trauma [504, 508, 513–518] and stroke literature [519–522]. In the setting of non-trauma-related ICH, a recent retrospective cohort analysis indicated that pre-injury APA administration was an independent risk factor for death within 7 days (OR 5.12; *P* = 0.006) and within 90 days (HR 1.87; *P* = 0.006) [522], but a systematic review, which did not include the latter study, showed that pre-ICH APA users experienced only modestly increased mortality (OR 1.27; 95 % CI 1.10 to 1.47) and little or no increase in poor clinical functional outcome (OR 1.10; 95 % CI 0.93 to 1.29) [523]. In patients with blunt head trauma, a meta-analysis of case-control and cohort studies showed only a slight and non-significant increased risk of death in patients who were taking pre-injury APA [524]. However, the effect of pre-injury APA on traumatic ICH is still controversial as more recent studies found both an association of worsening of the lesion [525, 526] and need for neurosurgical intervention [526] or no influence on survival and need for neurosurgical intervention [527].

Few studies have directly focused on outcome associated with a specific APA. Those that have analysed the use of clopidogrel prior to both spontaneous and traumatic ICH reported worsened outcome compared to controls: increased mortality [518, 520], increased morbidity [528], including progression of the lesion [503, 508, 520, 529], need for neurosurgical intervention [503, 529] and an increase in disposition to a long-term facility [518, 520]. Pre-injury aspirin did not affect outcomes in mild to moderate head injury [504, 530] or mortality [458] in observational studies but increased haemorrhage volume and mortality in one RCT [531]. Surprisingly, reduced platelet activity has been shown in patients with ICH in the absence of known aspirin use [458, 532] and this was associated with more ICH volume growth and worse 3-month outcome [533].

Early platelet dysfunction was also prevalent after severe TBI in the absence of APA treatment [534] and impaired platelet function (with or without the use of APA) demonstrated using an aspirin detection assay was associated with increased haematoma volume [516]. However, greater platelet inhibition was identified among patients taking a combination of APAs compared to those on single agents [532].

Lower platelet counts add additional risks. TBI patients on pre-hospital APA with a platelet count of 135 × 10^9^/l or less were 12.4 times (95 % CI 7.1 to 18.4) more likely to experience progression of initial ICH on repeated head CT scan; those with a platelet count of 95 × 10^9^/l or less were 31.5 times (95 % CI 19.7 to 96.2) more likely to require neurosurgical intervention [444].

These findings, coupled with the fact that 20–30 % of patients are non-responders to aspirin, clopidogrel or both agents [535], suggest that reliable measures of platelet function would be useful in the setting of the bleeding trauma patient to guide clinicians in the use of platelet transfusion or other reversal agents. Patients with occult platelet dysfunction who would benefit from platelet transfusion could be identified [536] or unnecessary platelet transfusion avoided [458].

Currently, there is no agreement on the optimal assay for platelet function, and controversy exists as to whether ICH in the setting of APA use warrants platelet transfusion. Transfusion of platelets has a low grade recommendation in the guidelines on ICH management in patients on APA [537] and is currently indicated for patients on clopidogrel and traumatic haemorrhage, although its clinical utility remains to be established [538]. Retrospective studies have failed to show an outcome benefit from platelet transfusion in patients on APA with spontaneous [521, 522, 539] or traumatic [514, 540, 541] ICH. A meta-analysis that included six small studies on the impact of platelet transfusion on survival in patients with pre-injury APA who experienced ICH, either spontaneous or traumatic, found no clear benefit [542]. Similarly, a systematic review of five retrospective registry studies on traumatic ICH provides inadequate evidence to support the routine use of platelet transfusion in patients with pre-injury antiplatelet use [505]. However, the timing of platelet administration was not optimal in some studies [533, 539], and a small prospective study showed that early platelet transfusion, within 12 h of symptom onset, improved platelet activity and was associated with smaller final haemorrhage size and more independence at 3 months [543].

An in vitro study performed in healthy volunteers taking aspirin and clopidogrel showed that an equivalent of two to three platelet pools could normalise platelet function in patients treated with APA [544]. However, further studies on the effect of platelet transfusion on platelet function in patients with traumatic ICH have been conflicting and inconclusive [458, 459, 545–547]. Platelet transfusion restored platelet function measured using an antiplatelet detection assay in patients on aspirin in some studies [458, 545], but not in others [546] and not in patients on clopidogrel [545]. In contrast, the effect of ex vivo platelet supplementation on platelet aggregation in blood samples from patients treated with aspirin, clopidogrel or ticagrelor showed improved aggregation independent of antiplatelet therapy [547]. However, while the aspirin effect was completely reversed, the recovery of ADP-dependent aggregation was limited even with a high dose of platelets (up to five apheresis units). One small prospective trial also showed that platelet transfusion improved aspirin-induced but not collagen trauma-induced platelet dysfunction measured using multiple electrode aggregometry (MEA) in patients with isolated TBI [459]. The outcome benefit of platelet transfusion in patients with non-traumatic ICH on aspirin is supported by a recent RCT [531]. These divergent results could be explained by the different amounts of platelets transfused, from one pack [546] to three to five units of apheresis platelets [458]. Another explanation for the observation that platelet transfusion shows no obvious benefit is that the inhibitory effect of the APA is not normalised due to recent ingestion of APA, which may also inactivate transfused platelets [543]. The results of a multicentre RCT on platelet transfusion in patients with APA-associated ICH are awaited [548].

The suggested dose for normalisation of platelet activity in healthy volunteers given aspirin alone or a combination of aspirin and clopidogrel was five and ten to 15 platelet units, respectively [544]. Successful perioperative management of patients on aspirin and clopidogrel requiring urgent surgery using two apheresis platelet units was recently reported [549]. Given that an active metabolite of clopidogrel persists after cessation of the medication and that the half-life of transfused platelets is short, recurring platelet transfusion may be justified [550].

Besides platelet transfusion, current potential antiplatelet reversal therapies include desmopressin and recombinant activated coagulation factor VII (rFVIIa) [538]. The rationale for treatment with desmopressin in patients treated with aspirin alone is included as part of Recommendation 32 (see next section). In healthy volunteers, rFVIIa reversed the inhibitory effects of aspirin and clopidogrel [551]. Interestingly, the effective dose was lower than the dose used in haemophilia patients [552]. In addition, TXA was shown to partially improve platelet function in patients treated with dual antiplatelet therapy as measured using MEA [553]. Potential effectiveness in improving haemostasis in trauma patients receiving APA was also shown for fibrinogen concentrate [554].

#### Desmopressin

##### *Recommendation 32*

**We suggest that desmopressin (0.3 μg/kg) be administered in patients treated with platelet-inhibiting drugs or with von Willebrand disease. (Grade 2C)**

**We do not suggest that desmopressin be used routinely in the bleeding trauma patient. (Grade 2C)**

#### Rationale

Desmopressin (DDAVP; 1-deamino-8-D-arginine vasopressin) enhances platelet adherence and platelet aggregate growth on human artery subendothelia and is the first choice in the treatment of bleeding in patients with von Willebrand disease, a disorder which occurs in roughly 1 in 100 patients [555, 556]. Two meta-analyses in patients not diagnosed with von Willebrand disease [557, 558] were able to demonstrate either a trend towards a reduced perioperative blood loss [557] or a small significant reduction in blood transfusion requirements [-0.29 (-0.52 to -0.06) units per patient] [558]. Patients with impaired platelet function as assessed by a platelet function analyser [559] or whole blood multiple electrode aggregometer [560] may benefit from desmopressin therapy. Concerns regarding possible thromboembolic complications [561] were not confirmed in the last meta-analysis from 2008 [558].

Although desmopressin has been shown to improve platelet function in volunteers on aspirin [562] and clopidogrel [563] and perioperatively in patients with mild inherited platelet defects [564], the use of desmopressin for acquired bleeding disorders is not supported by sound clinical evidence. One older meta-analysis suggested a benefit of desmopressin in patients taking aspirin [565], and desmopressin has been recommended in patients taking platelet inhibitors who suffer an ICH [538, 566]. The standard dose is 0.3 μg/kg diluted in 50 ml saline and infused over 30 min [564]. Recently, two small prospective studies have shown that desmopressin can improve platelet function in patients with ICH who have received aspirin [567] or not [568] prior to the event. Identification of impaired platelet function with a platelet function analyser PFA-100 [559] or whole blood MEA [560] might be helpful in the identification of patients who could benefit from desmopressin therapy. The combined effect of platelet concentrates and subsequent administration of desmopressin has also been advocated to enhance the recovery of normal platelet function [569], however, desmopressin and platelet administration was not associated with either a decreased risk of early radiographic haemorrhage progression (OR 1.40, 95 % CI 0.80 to 2.40; *P* = 0.2) or mortality (OR 1.50, 95 % CI 0.60 to 4.30; *P* = 0.4) in patients with traumatic ICH [570].

Desmopressin appears to be efficacious in the mitigation of platelet inhibition by adenosine diphosphate receptor inhibitors such as clopidogrel [571] and ticagrelor [572]. Equivalent data for prasugrel appear not to have been published.

There are only a few studies on the use of desmopressin in general trauma, ICH or TBI [538]. However, in patients with ICH and reduced platelet activity and/or prior aspirin use, desmopressin (0.4 µg/kg) shortened platelet function analyser closure time and increased von Willebrand factor levels [568]. Conversely, in a recent retrospective study on early ICH progression in 401 patients with TBI (54 on platelet inhibitors prior to trauma) the co-administration of desmopressin (0.3 µg/kg) with platelet transfusion was found inefficacious in terms of slowing the early ICH progression [570]. Nevertheless, desmopressin has been recommended in patients treated with platelet inhibitors with intracerebral bleeding [538, 566] and in trauma patients with von Willebrand disease [573]. Interestingly, desmopressin prevents the development of hypothermia-induced impairment of primary haemostasis [574] and significantly increases platelet aggregation during hypothermia and acidosis [575].

#### Prothrombin complex concentrate

##### *Recommendation 33*

**We recommend the early use of prothrombin complex concentrate (PCC) for the emergency reversal of vitamin K-dependent oral anticoagulants. (Grade 1A)**

**We suggest the administration of PCC to mitigate life-threatening post-traumatic bleeding in patients treated with novel oral anticoagulants. (Grade 2C)**

**Provided that fibrinogen levels are normal, we suggest that PCC or plasma be administered in the bleeding patient based on evidence of delayed coagulation initiation using viscoelastic monitoring. (Grade 2C)**

#### Rationale

The use of PCC has been shown to be superior to FFP in the rapid reversal of vitamin K antagonists [576–578] with evidence of less haematoma formation in those with head injury [579, 580]. It is therefore the agent of choice to reverse the effects of vitamin K antagonists [581].

No universally adopted reversal strategies for the non-vitamin K antagonist oral anticoagulants (NOAC) have been established, but despite limited clinical evidence, though data from animal studies exist [582], PCC has been used anecdotally to reverse the effect of NOAC [582–586]. The specific approach and rationale in patients on new oral anticoagulants are outlined in the recommendations on novel anticoagulants (R34-35).

Thromboelastometry appears to be a useful tool to guide PCC therapy in patients with traumatic coagulopathy [12, 587–591]. With an ageing population, more trauma patients are likely to have been pre-treated with vitamin K antagonists or oral direct inhibitors, therefore every trauma unit should have an established management policy for these patients [592, 593].

Because there are variations in the composition of PCC, the dosage should be determined according to the instructions of the individual manufacturer [594, 595]. A retrospective study that included 42 patients with warfarin-associated TBI and an INR ≥1.5 examined the effect of different doses of PCC. A dose of 35 IU/kg PCC compared to 25 IU/kg was associated with a higher percentage of INR reversal and a more rapid time (median time to INR reversal 6.9 h in the low-dose group and 1.9 h in the moderate-dose group) to INR normalisation in patients with TBI. In contrast, a RCT in patients with vitamin K antagonist-associated ICH showed no difference between two doses (25 IU/kg vs. 40 IU/kg) of four-factor PCC in terms of achieving target INR <1.5, however a lower INR was achieved with the higher dosage [596, 597].

The use of PCC is associated with an increased risk of both venous and arterial thrombosis during the recovery period, therefore the risk of thrombotic complications due to treatment with PCC should be weighed against the need for rapid and effective correction of coagulopathy [598–603]. Beyond emergency reversal of vitamin K antagonists, safety data on PCC used in trauma patients are scarce [604]. Activated PCC (aPCC) may be associated with a higher risk of thrombosis compared to non-activated PCC according to some expert opinion [605] due to presence of activated factor IX, because the thrombogenic trigger associated with PCC infusion occurs at the level of factor X activation as a part of aPCC [593]. In a study evaluating two doses of four-factor PCC in patients with vitamin K antagonist-associated ICH no safety concerns were raised regarding the 40 IU/kg dose [597]. Nevertheless, PCC administration to major trauma patients resulted in an increased endogenous thrombin potential over 3 days which was not reflected in standard laboratory coagulation tests [371]. Therefore, thromboprophylaxis as early as possible after control of bleeding has been achieved is prudent in patients who have received PCC.

#### Direct oral anticoagulants – factor Xa inhibitors

##### *Recommendation 34*

**We suggest the measurement of plasma levels of oral anti-factor Xa agents such as rivaroxaban, apixaban or edoxaban in patients treated or suspected of being treated with one of these agents. (Grade 2C)**

**If measurement is not possible or available, we suggest that advice from an expert haematologist be sought. (Grade 2C)**

**If bleeding is life-threatening, we suggest treatment with TXA 15 mg/kg (or 1 g) intravenously and high-dose (25-50 U/kg) PCC/aPCC until specific antidotes are available. (Grade 2C)**

#### Direct oral anticoagulants – thrombin inhibitors

##### *Recommendation 35*

**We suggest the measurement of dabigatran plasma levels in patients treated or suspected of being treated with dabigatran. (Grade 2C)**

**If measurement is not possible or available, we suggest thrombin time and APTT to allow a qualitative estimation of the presence of dabigatran. (Grade 2C)**

**If bleeding is life-threatening, we recommend treatment with idarucizumab (5 g intravenously) (Grade 1B), or, if unavailable, we suggest treatment with high-dose (25–50 U/kg) PCC/aPCC, in both cases combined with TXA 15 mg/kg (or 1 g) intravenously. (Grade 2C)**

#### Rationale

In recent years, direct oral anticoagulants for the prevention of VTE, prevention of stroke in atrial fibrillation, acute coronary syndrome and treatment of pulmonary embolism (PE) and deep venous thrombosis (DVT) have been developed. The primary modes of action by these novel drugs are direct factor Xa inhibition (rivaroxaban, apixaban and edoxaban) or thrombin inhibition (dabigatran) [606]. Physicians are therefore increasingly likely to be confronted with trauma patients who have been treated with one of these drugs [607], which exert an effect on both coagulation tests [607, 608] and haemostasis [609].

No published clinical studies and very little clinical experience in trauma patients who have been treated with one of these drugs exist [608, 610]. However, animal studies and ex vivo human studies on the effect of three- and four-factor PCC/aPCC and recombinant factor VIIa have been published. In summary, although not completely consistent, laboratory coagulation tests, parameters of viscoelastic tests and of thrombin generation were (nearly) normalised with high-dose treatment [611–619]. Whether this effect results in improved haemostasis with reduced bleeding may depend on the level of the anticoagulants; no effect on bleeding was seen at a rivaroxaban plasma concentration of approximately 500–700 ng/ml in rabbits [609] while a concomitant reduction in bleeding was found at a dabigatran plasma concentration of 65 ng/ml in mice [620]. Also in rats, progressive doses of four-factor PCC were able to reverse the bleeding volume [621]. At a rivaroxaban plasma concentration of approximately 150 ng/ml bleeding volume was normalised with a PCC dose of 25 U/kg, at a rivaroxaban plasma concentration of approximately 280 ng/ml normalisation of bleeding required a PCC dose of 50 U/kg and at a rivaroxaban plasma concentration of approximately 480 ng/ml even the administration of 100 U/kg PCC was unable to reduce the elevated blood loss [621].

Measurement of the plasma concentration of these anticoagulants is recommended in order to ascertain whether and to what extent these agents might exert and influence the coagulation system [622]. There are no threshold values above which a significant effect is to be expected, since the effect is gradual with increasing plasma concentration [621]. However, low concentrations (<30 ng/ml) may be regarded as having a very mild and likely a clinically insignificant effect [622]. High levels (>200–300 ng/ml) are likely to seriously compromise coagulation, and fatal exsanguinations have been described.

If factor Xa antagonist treatment is known or suspected, anti-factor Xa activity can be measured using a substrate-specific anti-factor Xa test. If unavailable, anti-factor Xa activity tests for low molecular weight heparin (LMWH) can be used to gather qualitative information about the presence of a factor Xa antagonist. If factor IIa antagonist treatment is known or suspected, dabigatran-calibrated thrombin time can be measured. Factor Xa and IIa inhibitors have an effect on viscoelastic tests [623], however these tests provide an overall snapshot of the coagulation state, and the observed changes cannot be used to estimate the specific effect of Xa/IIa inhibition on coagulation. If measurement is not possible or available, thrombin time and APTT can be used to qualitatively assess the presence of dabigatran. If anti-factor Xa activity is detected, high-dose (25–50 U/kg) PCC/aPCC treatment may be initiated. We suggest an initial dose of 25 U/kg, repeated if necessary, as a cautious approach given the possible thrombotic potential of PCC/aPCC products [599]. In the presence of anti-FIIa activity due to dabigatran, treatment with dabigatran antidote idarucizumab (5 g i.v.) should be initiated [624, 625], or if unavailable, preoperative haemodialysis considered [626, 627]. The co-administration of TXA is generally indicated in trauma patients (see Recommendation 25). In addition, in patients undergoing hip replacement surgery with rivaroxaban thromboembolic prophylaxis, the use of TXA reduced postoperative blood loss [628]. The use of recombinant factor VIIa has been described, but cannot be recommended as a first-line treatment. The involvement of a haematologist with expertise in coagulation should be considered.

As of late 2015 idarucizumab, the antidote to dabigatran, had received marketing approval from the US Food and Drug Administration (FDA) and the European Medicines Agency (EMA). Specific antidotes against Xa antagonists are in development, including andexanet alfa, a specific factor Xa inhibitor-reversing agent [629], however, these are not yet approved for clinical use [630, 631].

#### Recombinant activated coagulation factor VII

##### *Recommendation 36*

**We suggest that the off-label use of rFVIIa be considered only if major bleeding and traumatic coagulopathy persist despite all other attempts to control bleeding and best-practice use of conventional haemostatic measures. (Grade 2C)**

#### Rationale

rFVIIa should be considered only if first-line treatment with a combination of surgical approaches, best-practice use of blood products, (RBC, platelets, FFP, and cryoprecipitate/fibrinogen resulting in a Hct above 24 %, platelets above 50 × 10^9^/l and fibrinogen above 1.5–2.0 g/l), the use of antifibrinolytics and correction of severe acidosis, severe hypothermia and hypocalcaemia fail to control bleeding.

rFVIIa acts on the patient’s own coagulation system and adequate numbers of platelets and fibrinogen levels are needed to support activity [632, 633]. pH and body temperature should be restored as near to physiological levels as possible, since even small reductions in pH and temperature result in slower coagulation enzyme kinetics [299, 300, 634]. Predictors of a poor response to rFVIIa are a pH <7.2 (*P* < 0.0001), a platelet count <100 × 10^9^/l (*P* = 0.046), and blood pressure ≤90 mmHg (*P* < 0.0001) [635]. In one study administration of rFVIIa to patients with a pH of <6.9 appeared futile [636]. In another study from the The Australian and New Zealand Haemostasis Registry a pH <7.1 prior to rVFIIa administration was independently associated with an increased 28-day mortality [637]. Moreover, hypocalcaemia is frequently present in severely injured patients [638], therefore monitoring of ionised calcium is necessary, and administration of intravenous calcium may be required [639].

Despite numerous case studies and series reporting that treatment with rFVIIa can be beneficial in the treatment of bleeding following trauma, there are few high-quality studies [640–643]. A multicentre, randomised, double-blind, placebo-controlled study examined the efficacy of rFVIIa in patients with blunt (n = 143) or penetrating (n = 134) trauma [644] and showed that patients with blunt trauma who survived for more than 48 h assigned to receive rFVIIa 200 μg/kg after they had received eight units of RBC and a second and third dose of 100 μg/mg 1 and 3 h later had a reduction in RBC transfusion requirements and the need for massive transfusions (>20 units of RBC) compared to placebo. They also had a significantly reduced incidence of ARDS. In contrast, there were no significant effects in the penetrating trauma patients in this study, although trends towards reduced RBC requirements and fewer massive transfusions were observed. Similar results and trends were observed in other retrospective studies and case reports [645–647]. A further randomised clinical trial [648] aimed to evaluate rFVIIa as an adjunct to direct haemostasis in major trauma patients who bled four to eight RBC units within 12 h of injury and were still bleeding despite strict damage control resuscitation and operative management. Patients were treated with rFVIIa (200 μg/kg initially; 100 μg/kg at 1 and 3 h) or placebo. The trial was terminated early (n = 573) due to difficulty in consenting and enrolling sicker patients and resulting low mortality rates that prompted a futility analysis. Thrombotic adverse events were similar across study cohorts.

A recent study from the German trauma registry comparing two matched groups of 100 patients each with or without early administration of rFVIIa found no difference in mortality or transfusion requirements between groups, however, there was an increased incidence of multiple organ failure in the rFVIIa group (82 % vs. 62 %) [649]. In a retrospective study of thromboelastographic-guided haemostatic therapy in 38 abdominal trauma patients, 20 patients who received rFVIIa (average dose 52.3 μg/kg) experienced decreased R time and were transfused with RBC, platelets and FFP significantly less compared to 18 patients not given rFVIIa [650].

In contrast, the use of rFVIIa in isolated head injury was found to be harmful in a case-controlled study of patients with traumatic ICH, with the risk of death appearing to increase with administration regardless of the severity of injury [651]. No reliable evidence from RCTs exists to support the effectiveness of haemostatic drugs in reducing mortality or disability in patients with TBI [652]. In warfarin-treated patients with TBI the use of recombinant factor VIIa did not improve mortality or reduce the use of plasma [653]. As there is no evidence that would lead a clinician to consider rFVIIa in ICH caused by isolated head trauma, the previous negative recommendation – “We do not suggest the use of rFVIIa in patients with intracerebral haemorrhage caused by isolated head trauma” has been removed from this version of the guideline, as this conclusion is self-evident.

If used, the dose(s) of rFVIIa is still under debate. Whereas the dosing administered in the published RCTs in trauma patients was recommended by a group of European experts [654], Israeli guidelines based on findings from a case series of 36 patients who received rFVIIa on a compassionate-use basis [641] proposed an initial dose of 120 μg/kg (between 100 and 140 μg/kg) and (if required) a second and third dose. Pharmacokinetic modelling techniques have shown that the dose regimen for rFVIIa treatment used in the RCT described above is capable of providing adequate plasma levels of drug to support haemostasis [655]. Bain et al. compared their institutional rFVIIa low-dose protocol to previous practice using higher doses of rFVIIa. The total dose of rFVIIa in pre-protocol patients (n = 80) was significantly higher (62 μg/kg) compared to 48 μg/kg in post-protocol patients (n = 117) but no differences were found in outcome measures such as mortality, blood product use or adverse events [656].

In a recent prospective non-randomised trial evaluating 87 patients with isolated TBI and coagulopathy at admission, in addition to blood products 38 patients were administered a single dose of rFVIIa (20 μg/kg) intravenously. Not surprisingly, the improvement in INR as a primary outcome measure was significantly greater in the rFVIIa group, but hospital mortality was similar in both groups [657].

If rFVIIa is administered and if possible, the patient and/or next of kin should be informed that rFVIIa is being used outside the currently approved indications (off-label use), especially since the use of rFVIIa may increase the risk of thromboembolic complications [658]. A meta-analysis showed a higher risk of arterial thromboembolic adverse events (5.6 % in patients receiving rFVIIa versus 3.0 % in placebo-treated patients) among over 2000 patients enrolled in placebo-controlled trials outside currently approved indications in various clinical settings [659]. In trauma patients, rFVIIa use was not associated with an increased risk of thromboembolic complications [660]. In a recent retrospective single-centre cohort study that analysed 152 surgical and trauma patients that received different doses of off-label rFVIIa, the overall incidence of thromboembolic events was 12.5 % without any difference between low (30 μg/kg) and high dose (100 μg/kg) rFVIIa. A higher incidence of thromboembolic events (approximately 21 %) was found in cardiothoracic surgery and penetrating trauma [661].

#### Thromboprophylaxis

##### *Recommendation 37*

**We recommend pharmacological thromboprophylaxis within 24 h after bleeding has been controlled. (Grade 1B)**

**We recommend early mechanical thromboprophylaxis with intermittent pneumatic compression (IPC) (Grade 1C) and suggest early mechanical thromboprophylaxis with anti-embolic stockings. (Grade 2C)**

**We do not recommend the routine use of inferior vena cava filters as thromboprophylaxis. (Grade 1C)**

#### Rationale

The risk of hospital-acquired VTE is high after multiple trauma, exceeding 50 %; PE is the third leading cause of death in those who survive beyond the third day [662]. There are few RCTs that have investigated thromboprophylaxis in trauma patients, and the use of anti-embolic stockings has never been evaluated in this group. A meta-analysis was unable to show any reduction in the rate of DVT with IPC [663], however mechanical methods are widely used because of the low bleeding risk.

A systematic review and meta-analysis [664] showed that any type of heparin thromboprophylaxis decreases DVT and PE in medical-surgical critically ill patients, and LMWH compared with twice daily unfractionated heparin (UFH) decreases both the overall rate and symptomatic rate of PE. Major bleeding and mortality rates did not appear to be significantly influenced by heparin thromboprophylaxis in the ICU setting. Another study of 289 patients who developed VTE during or after a critical care stay showed that thromboprophylaxis failure was more likely with elevated body mass index, a personal or family history of VTE and those administered vasopressors [665].

Side effects associated with the use of heparin include heparin-induced thrombocytopenic thrombosis. This effect is seen more frequently with UFH than LMWH. The severity of trauma has been associated with the risk of heparin-induced thrombocytopenia, therefore the greater the risk, the greater the importance of monitoring platelet counts in trauma patients [666]. In summary, the use of heparin once haemostasis has been achieved is the most efficacious option for trauma patients. In those with a bleeding risk, mechanical methods are preferable. Due to the varied results from trials comparing UFH with LMWH, we do not recommend one over the other. Because LMWHs are mainly excreted renally, unlike UFH, which is excreted via the liver as well, there is risk of accumulation in patients with renal failure, therefore dose adjustments and/or monitoring should be performed with LMWH according to the manufacturer’s instructions.

Contraindications to pharmacological thromboprophylaxis include patients already receiving full-dose anticoagulation, those with significant thrombocytopenia (platelet count <50 × 10^9^/l), an untreated inherited or acquired bleeding disorder, evidence of active bleeding, uncontrolled hypertension (blood pressure >230/120), a lumbar puncture/spinal analgesia expected within the next 12 h or performed within the last 4 h (24 h if traumatic), procedures with a high bleeding risk or a new haemorrhagic stroke, although a recent systematic review found that pharmacological thromboprophylaxis appears to be safe among patients with TBI and stabilised haemorrhagic patterns [667].

The use of prophylactic inferior vena cava filters is common; however no evidence of added benefit when used in combination with pharmacological thromboprophylaxis exists. PE still occur despite the presence of a filter, and filters have short- and long-term complication rates, are associated with high cost and often provide a false sense of security, delaying the use of effective pharmacological thromboprophylaxis. Furthermore, inferior vena cava filters require a second invasive procedure to remove.

The optimal timing for the initiation of pharmacological thromboprophylaxis is often difficult to judge. Data from 175,000 critical care admissions showed that the risk of mortality was higher in those who did not receive thromboprophylaxis during the first 24 h [668]. This reflects the concern that those who bleed have a higher rate of VTE than those who do not [669].

There is inadequate research on the use of mechanical thromboprophylaxis in critical care. The recent Clots in Legs or Stockings after Stroke (CLOTS 3) study was the first large RCT to look at the utility of IPC in 2876 stroke patients and showed a clear benefit with a reduction in DVT from 12.1 to 8.5 % and an absolute reduction of 3.6 % (95 % CI 1.4 to 5.8), with a non-significant reduction in death [670]. While the population in this study is different from those in critical care, both populations have similar risk factors (immobility and acute-phase response), which led us to upgrade the recommendation for IPC.

### VII. Guideline implementation and quality control

#### Guideline implementation

##### *Recommendation 38*

**We recommend the local implementation of evidence-based guidelines for management of the bleeding trauma patient. (Grade 1B)**

#### Assessment of bleeding control and outcome

##### *Recommendation 39*

**We recommend that local clinical quality and safety management systems include parameters to assess key measures of bleeding control and outcome. (Grade 1C)**

#### Rationale

Evidence to support the effectiveness of patient management algorithms in changing clinical care is weak, however local implementation of a multidisciplinary, evidence-based treatment algorithm or clinical management guideline for the bleeding trauma patient is likely to create awareness among all involved medical specialities and to improve mutual understanding. The local treatment algorithm allows, within the framework of the available evidence, flexibility to accommodate local pre-hospital rescue conditions, locally available diagnostic and therapeutic options and improves the consistency of care. However, any guideline is designed for the “average” patient, therefore the clinician must adapt and tailor treatment to best accommodate each individual case.

If key interventions described in a guideline are implemented, outcomes are likely to be improved [671, 672] and death and other complications reduced [673]. Moreover, treatment according to management guidelines may be associated with cost savings [674]. Unfortunately, strict guideline adherence is often challenging in a complex case with poor prognosis, therefore the association between guideline adherence and good outcomes is not necessarily causal.

The implementation of our recommendations might be facilitated by a checklist approach analogous to the Safe Surgery Initiative [675], which led to fewer postoperative complications [676]. In addition or alternatively, it may be possible to implement our recommendations using bundles as has been successfully achieved during implementation of the Surviving Sepsis Campaign guidelines [677]. Suggested items that should be included in such a checklist are summarised in Table 4. Suggested patient management bundles are listed in Table 5.

**Table 4:** Treatment pathway checklist

Treatment phase		Yes	No	N/A	Reason for variance
Initial assessment and management					
	Extent of traumatic haemorrhage assessed	☐	☐	☐	
	Patient in shock with identified source of bleeding treated immediately	☐	☐	☐	
	Patient in shock with unidentified source of bleeding sent for further investigation	☐	☐	☐	
	Coagulation, haematocrit, serum lactate, base deficit assessed	☐	☐	☐	
	Antifibrinolytic therapy initiated	☐	☐	☐	
	Patient history of anticoagulant therapy assessed (vitamin K antagonists, antiplatelet agents, oral anticoagulants)	☐	☐	☐	
Resuscitation					
	Systolic blood pressure of 80–90 mmHg achieved in absence of traumatic brain injury	☐	☐	☐	
	Measures to achieve normothermia implemented	☐	☐	☐	
	Target haemoglobin level 7–9 g/dl achieved	☐	☐	☐	
Surgical intervention					
	Abdominal bleeding control achieved	☐	☐	☐	
	Pelvic ring closed and stabilised	☐	☐	☐	
	Peritoneal packing, angiographic embolisation or surgical bleeding control completed in haemodynamically unstable patient	☐	☐	☐	
	Damage control surgery performed in haemodynamically unstable patient	☐	☐	☐	
	Local haemostatic measures applied	☐	☐	☐	
	Thromboprophylactic therapy recommended	☐	☐	☐	
Coagulation management					
	Coagulation, haematocrit, serum lactate, base deficit, calcium reassessed	☐	☐	☐	
	Target fibrinogen level 1.5–2 g/l achieved	☐	☐	☐	
	Target platelet level achieved	☐	☐	☐	
	Prothrombin complex concentrate administered if indicated due to vitamin K antagonist, oral anticoagulant or evidence from viscoelastic monitoring	☐	☐	☐

**Table 5:** Suggested management bundles

Pre-hospital bundle	Intra-hospital bundle	Coagulation bundle
• Pre-hospital time minimised • Tourniquet employed in case of life-threatening bleeding from extremities • Damage control resuscitation concept applied • Trauma patient transferred directly to an adequate trauma specialty centre	• Full blood count, prothrombin time, fibrinogen, calcium, viscoelastic testing, lactate, BE and pH assessed within the first 15 min • Immediate intervention applied in patients with haemorrhagic shock and an identified source of bleeding unless initial resuscitation measures are successful • Immediate further investigation undertaken using focused assessment with sonography for trauma (FAST), computed tomography (CT) or immediate surgery if massive intra-abdominal bleeding is present in patients presenting with haemorrhagic shock and an unidentified source of bleeding • Damage control surgery concept applied if shock or coagulopathy are present • Damage control resuscitation concept continued until the bleeding source is identified and controlled • Restrictive erythrocyte transfusion strategy (haemoglobin 7–9 g/dl) applied	• Tranexamic acid administered as early as possible • Acidosis, hypothermia and hypocalcaemia treated • Fibrinogen maintained at 1.5–2 g/l • Platelets maintained at >100 × 10^9^/l • Prothrombin complex concentrate administered in patients pre-treated with warfarin or direct-acting oral coagulants (until antidotes are available)

Training in trauma care should emphasise the key role of coagulation in determining outcome. Increasing clinician knowledge and understanding in this area should be an integral part of the implementation of the algorithm. All trauma care centres should evaluate their own performance using a routine institutional quality management programme. An audit of adherence to best practice, including feedback and practice change where needed should be included as part of the local implementation of these guidelines. In order to evaluate the quality of care provided to the patient who is bleeding after major trauma, we suggest that adherence to the following quality standards be assessed:

- Time from injury to the initiation of intervention to stop bleeding (surgery or embolisation) in hypotensive patients who do not respond to initial resuscitation.
- Time from hospital arrival to availability of a full set of blood results [full blood count, PT, fibrinogen, calcium, viscoelastic testing (if available)].
- Proportion of patients receiving TXA within 3 h after injury.
- Time from hospital arrival to CT scan in bleeding patients without an obvious source of haemorrhage.
- Damage control surgical techniques used in accordance with Recommendation 19.
- Thromboprophylaxis commenced in accordance with Recommendation 37.

## Discussion

**Fig. 2:**
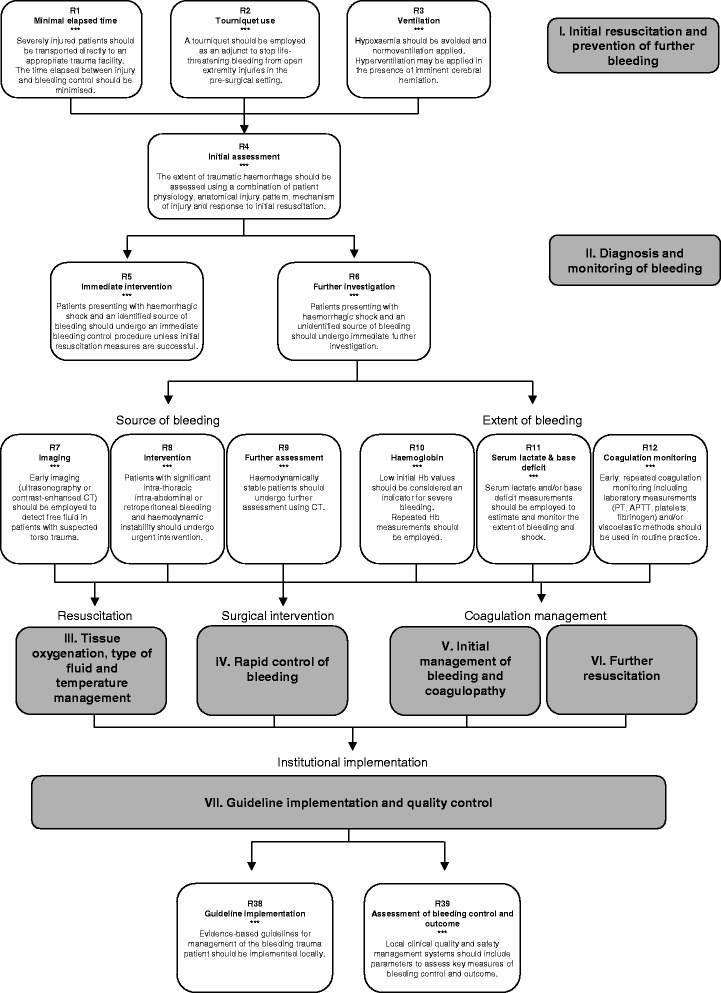
Summary of treatment modalities for the bleeding trauma patient included in this guideline (part 1 of 2). APTT, activated partial thromboplastin time; CT, computed tomography; Hb, haemoglobin; PT, prothrombin time

**Fig. 3:**
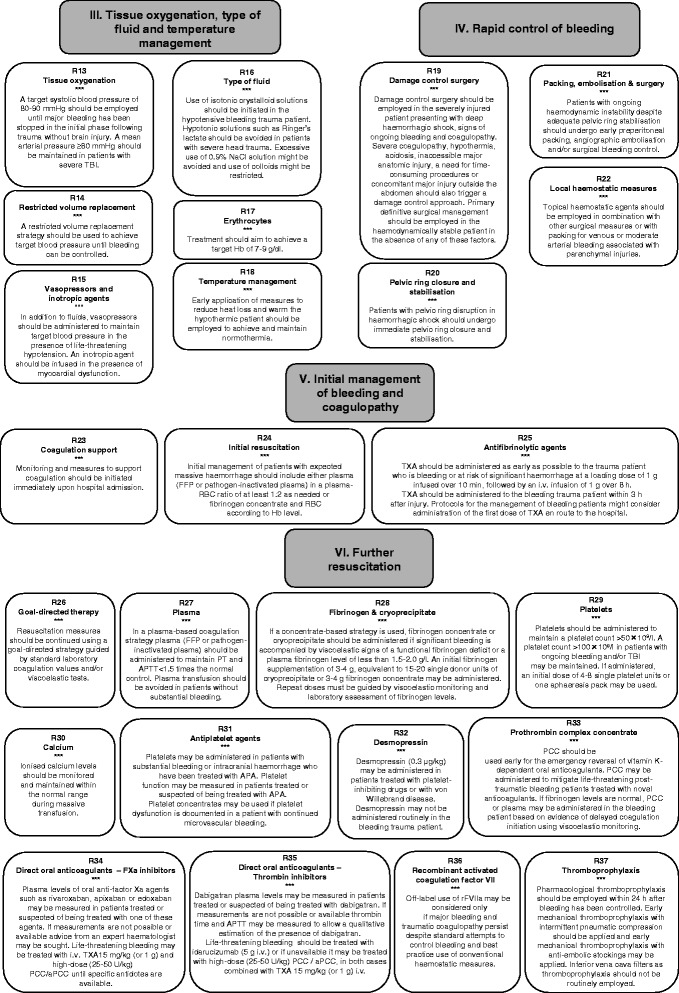
Summary of treatment modalities for the bleeding trauma patient included in this guideline (part 2 of 2). APA, antiplatelet agents; aPCC, activated PCC; APTT, activated partial thromboplastin time; FFP, fresh frozen plasma; Hb, haemoglobin; i.v., intravenous; PCC, prothrombin complex concentrate; PT, prothrombin time; RBC, red blood cells; rFVIIa, recombinant activated coagulation factor VIIa; TBI, traumatic brain injury; TXA, tranexamic acid

These guidelines on the management of significant bleeding and coagulopathy following major trauma reflect the current published literature as identified using structured queries to identify relevant published abstracts and full publications. Expert opinion and current clinical practice were also considered, particularly in areas in which randomised clinical trials have not or cannot be performed for practical or ethical reasons. Recommendations published in previous editions of the guideline [32–34] were reconsidered and revised based on new scientific evidence and observed shifts in clinical practice as appropriate. In addition, new recommendations were formulated to reflect current clinical concerns and areas in which new research data have been generated. All recommendations were developed using a consensus process among the author group, comprising a multidisciplinary, pan-European task force that includes representatives from relevant European professional societies. Figures 2 and 3 graphically summarise the current recommendations included in this guideline.

In the initial resuscitation phase of treatment, the current edition of the guideline now recommends not only that the time between injury and bleeding control be minimised, but that the severely injured patient be transferred directly to an appropriate trauma treatment centre, which may not be the same as the nearest medical facility. The recommendations on ventilation measures have also now been refined to include a general recommendation to avoid hypoxaemia (Grade 1A), normoventilation in the bleeding trauma patient in general (Grade 1B), but with a suggestion to apply hyperventilation to the brain-injured patient (Grade 2C) to decrease intracranial pressure. The former recommendation to avoid the use of a single Hct measurement as a marker for bleeding has also been differentiated to recommend that a low initial Hct value serve as a signal for possible severe bleeding and coagulopathy, but that monitoring continue even in the presence of an initial normal value (both Grade 1B).

A new section has been added to specifically recommend a restricted volume replacement strategy (Grade 1B) and the recommendations on fluid therapy have been condensed to generally recommend the initial use, if any, of isotonic crystalloid solutions (Grade 1A), but avoid excessive use of 0.9 % NaCl (Grade 2C), colloid solutions (Grade 2C) and hypotonic solutions such as Ringer’s lactate in patients with head injury (Grade 1C). The chapter on surgical interventions has been updated with publications that have become available in the interim where appropriate, but the overall recommendations have not changed compared to the previous edition of the guideline.

To reflect different strategic approaches that depend on the availability of rapid point-of-care coagulation testing to facilitate goal-directed therapy, a new section has been added to the chapter on the initial management of bleeding and coagulopathy that recommends either the use of plasma and erythrocytes in a ratio of at least 1:2 (Grade 1B) or fibrinogen concentrate and erythrocytes (Grade 1C). Similarly, further resuscitation measures should be guided by a goal-directed strategy (Grade 1C) using either the conventional blood products or a factor concentrate-based strategy. The sections that discuss the management of patients pre-treated with novel anticoagulants have been further expanded to reflect accumulating experience and awareness of the necessity of monitoring for potential exposure, particularly in the elderly population, and suggestions for treatment and haematological consultation (Grade 2C).

The present guideline should be viewed as an educational aid to improve and standardise the care of the bleeding trauma patients across Europe and beyond. The recommendations that comprise the final chapter continue to encourage the local implementation of evidence-based guidelines for the management of the bleeding patient following traumatic injury and that local quality and safety management systems specifically assess key measures of bleeding control and outcome.

## Conclusions

The appropriate management of trauma patients with massive bleeding and coagulopathy remains a major challenge in routine clinical practice. A multidisciplinary approach and adherence to evidence-based guidance are key to improving patient outcomes. The implementation of locally adapted treatment algorithms should strive to achieve measureable improvements in patient outcome.

## Key messages

- Traumatically injured patients should be transported as quickly as possible and treated by a specialised trauma centre whenever possible.
- Measures to monitor and support coagulation should be initiated as early as possible and used to guide resuscitation.
- A damage control approach to surgical intervention should guide patient management.
- Awareness of potential thrombotic risk and pre-treatment with anticoagulant agents, particularly in older patients, should be part of routine clinical management.
- Local adherence to a multidisciplinary, evidence-based treatment protocol should serve as the cornerstone of patient management and undergo regular quality assessment.

## Additional file

Additional file 1: MeSH terms and limits applied to address guideline literature queries – 2015. (PDF 419 kb)

## Abbreviations

ACIT: Activation of Coagulation and Inflammation in Trauma
ACS: abdominal compartment syndrome
APA: antiplatelet agents
aPCC: activated PCC
APTT: activated partial thromboplastin time
ARDS: acute respiratory distress syndrome
ATLS: Advanced Trauma Life Support
CLOTS 3: Clots in Legs or Stockings after Stroke
CRASH-2: Clinical Randomisation of Antifibrinolytic therapy in Significant Haemorrhage
CT: computed tomography
DDAVP: 1-deamino-8-D-arginine vasopressin
DVT: deep venous thrombosis
EMA: European Medicines Agency
EPO: erythropoietin
ESA: European Society of Anaesthesiology
ESICM: European Society of Intensive Care Medicine
ESS: European Shock Society
ESTES: European Society for Trauma and Emergency Surgery
EuSEM: European Society for Emergency Medicine
FDA: US Food and Drug Administration
FFP: fresh frozen plasma
GCS: Glasgow Coma Score
GRADE: Grading of Recommendations Assessment, Development and Evaluation
Hb: haemoglobin
Hct: haematocrit
HES: hydroxyethyl starch
i.v.: intravenous
ICH: intracranial haemorrhage
ICU: intensive care unit
INR: international normalised ratio
IPC: intermittent pneumatic compression
IQR: interquartile ratio
ISS: Injury Severity Score
LMWH: low molecular weight heparin
MATTERs II: Military Application of Tranexamic Acid in Trauma Emergency Resuscitation
MCF: maximum clot firmness
MEA: multiple electrode aggregometry
MeSH: medical subject headings
MSCT: multislice computed tomography
NATA: Network for the Advancement of Patient Blood Management, Haemostasis and Thrombosis
NE: norepinephrine
NOAC: non-vitamin K antagonist oral anticoagulants
PAHFRAC-01: Efficacy of Ferric Carboxymaltose With or Without EPO Reducing Red-cell Transfusion Packs in Hip Fracture Perioperative Period
PCC: prothrombin complex concentrate
PE: pulmonary embolism
PEEP: positive end-expiratory pressure
PT: prothrombin time
PROMMTT: Prospective, Observational, Multicenter, Major Trauma Transfusion
PROPPR: Pragmatic, Randomized Optimal Platelet and Plasma Ratios
PTOS: Pennsylvania Trauma Outcome Study
RBC: red blood cells
RCTs: randomised controlled trials
REBOA: resuscitative endovascular balloon occlusion of the aorta
rFVIIa: recombinant activated coagulation factor VII
RPH: retroperitoneal haemorrhage
RR: risk ratio
SAP: systolic arterial pressure
TASH: Trauma-Associated Severe Hemorrhage
TBI: traumatic brain injury
TRALI: transfusion-related acute lung injury
TRICC: Transfusion Requirements in Critical Care
TXA: tranexamic acid
UFH: unfractionated heparin
VTE: venous thromboembolism
WHO: World Health Organization
