# Supplementary material for: The European guideline on management of major bleeding and coagulopathy following trauma: fourth edition
Source: Crit Care. 2016 Apr 12;20:100. doi: 10.1186/s13054-016-1265-x (PMC4828865; doi:10.1186/s13054-016-1265-x)
Supplement: Additional file 1: — MeSH terms and limits applied to address guideline literature queries – 2015. (PDF 419 kb) [file 13054_2016_1265_MOESM1_ESM.pdf]

The European guideline on management of major bleeding and coagulopathy following trauma: Fourth edition

Rossaint R, Bouillon B, Cerny V, Coats TJ, Duranteau J, Fernández-Mondéjar E, Filipescu D, Hunt BJ, Komadina R, Nardi G, Neugebauer EAM, Ozier Y, Riddez L, Schultz A, Vincent J-L, Spahn DR

Additional data file 1. MeSH terms and limits applied to address guideline literature queries were performed on 30 January 2015; some additional and/or newer citations may have been added ad hoc in response to professional society and peer reviewer requests.

Question		Query	Limit
**I**		**INITIAL RESUSCITATION AND PREVENTION OF FURTHER BLEEDING**	
	**1**	**Does coagulopathy have an effect on outcome in patients with different types of injury?**	
		“Wounds and Injuries”[MeSH] AND “Blood coagulation disorders”[MeSH]	Clinical Trial, Meta-Analysis, Practice Guideline, Randomized Controlled Trial, Review, Humans, English, 2012-end
	**2**	**Does control of acid-base balance during the initial resuscitation affect outcome?**	
		"Wounds and Injuries"[MAJR] AND "Acid-Base Equilibrium"[MeSH]	Humans English 2012-end
	**3**	**Does the degree of initial bleeding affect coagulopathy?**	
	**4**	**Does the degree of initial bleeding affect outcome?**	
		“Wounds and Injuries”[MeSH] AND “Hemorrhage”[MeSH] AND “Hemostasis”[MeSH]	Humans English 2012-end
	**5**	**Does initial resuscitation with respect to haemostasis have an effect on outcome in patients with different types of injury?**	
		("Wounds and Injuries"[MeSH] OR "injuries"[Subheading] OR "Craniocerebral Trauma"[MeSH] OR "Trauma Severity Indices"[MeSH] OR "Trauma Centers"[MeSH] OR "Cumulative Trauma Disorders"[MeSH] OR "Cerebrovascular Trauma"[MeSH] OR "Multiple Trauma"[MeSH] OR "Trauma, Nervous System"[MeSH] OR "Head Injuries, Penetrating"[MeSH] OR "Abducens Nerve Injury"[MeSH] OR "Optic Nerve Injuries"[MeSH] OR "Coma, Post-Head Injury"[MeSH] OR "Facial Nerve Injuries"[MeSH] OR "Carotid Artery Injuries"[MeSH] OR "Head Injuries, Closed"[MeSH] OR "Spinal Cord Injuries"[MeSH] OR "Traumatology"[MeSH] OR "Brain Injuries"[MeSH] OR "Emergency Treatment"[MeSH] OR "Emergencies"[MeSH] OR "Critical Care"[MeSH] OR "Emergency Treatment"[MeSH]) AND ("Resuscitation"[MeSH] OR "Resuscitation Orders"[MeSH]) AND ("Hemostatic Techniques"[MeSH] OR "Hemostasis"[MeSH])	Humans English 2012-end
	**6**	**Is wound compression effective in preventing bleeding and coagulopathy?**	

	a	("Wounds and Injuries"[MeSH] OR "injuries"[Subheading] OR "Craniocerebral Trauma"[MeSH] OR "Trauma Severity Indices"[MeSH] OR "Trauma Centers"[MeSH] OR "Cumulative Trauma Disorders"[MeSH] OR "Cerebrovascular Trauma"[MeSH] OR "Multiple Trauma"[MeSH] OR "Trauma, Nervous System"[MeSH] OR "Head Injuries, Penetrating"[MeSH] OR "Abducens Nerve Injury"[MeSH] OR "Optic Nerve Injuries"[MeSH] OR "Coma, Post-Head Injury"[MeSH] OR "Facial Nerve Injuries"[MeSH] OR "Carotid Artery Injuries"[MeSH] OR "Head Injuries, Closed"[MeSH] OR "Spinal Cord Injuries"[MeSH] OR "Traumatology"[MeSH] OR "Brain Injuries"[MeSH] OR "Emergency Treatment"[MeSH] OR "Emergencies"[MeSH] OR "Critical Care"[MeSH] OR "Emergency Treatment"[MeSH]) AND ("Resuscitation"[MeSH] OR "Resuscitation Orders"[MeSH]) AND ("Hemostatic Techniques"[MeSH] OR "Hemostasis"[MeSH]) AND ("Bandages"[MeSH] OR "Tourniquets"[Mesh])	Humans<br>English<br>2012-end
	b	("Hemostatic Techniques"[MeSH] OR "Hemostasis"[MeSH]) AND ("Bandages"[MeSH] OR "Tourniquets"[Mesh])	Humans<br>English<br>2012-end
	c	"Tourniquets"[Mesh]	Humans<br>English<br>2012-end
	**7**	**Does the duration of the pre-hospital phase of initial resuscitation have an effect on outcome in patients with haemorrhagic shock?**	
		"Shock, Hemorrhagic"[MeSH] AND ("Emergency Medical Services"[MeSH] OR "Emergencies"[MeSH] OR "Ambulances"[MeSH] OR "Emergency Medical Technicians"[MeSH] OR "Emergency Treatment"[MeSH] OR "Emergency Medical Tags"[MeSH] OR "Emergency Nursing"[MeSH] OR "Emergency Medicine"[MeSH] OR "Emergency Medical Service Communication Systems"[MeSH] OR "Air Ambulances"[MeSH]) AND (pre-hospital OR prehospital)	Humans<br>English<br>2012-end
	**8**	**Does the amount of bleeding and type of injury influence the selection of the hospital to which the trauma patient should be transported?**	
		"Health Care Category"[MAJR] AND "Hemorrhage"[MeSH] AND "Wounds and Injuries"[MeSH] AND ("Emergencies"[MeSH] OR "Emergency Treatment"[MeSH] OR "Emergency Medicine"[MeSH] OR "Emergency Medical Technicians"[MeSH])	Humans<br>English<br>2012-end
	**9**	**Does the gender of the bleeding trauma patient affect outcome?**	
	**10**	**Does the age of the bleeding trauma patient affect outcome?**	
	a	“Wounds and Injuries”[MeSH] AND “Hemorrhage”[MeSH] AND “Hemostasis”[MeSH] AND ("sex"[MeSH Terms] OR "Age Factors"[Mesh])	Humans<br>English
	b	"Wounds and Injuries"[MeSH] AND "Blood coagulation disorders"[MeSH] AND ("sex"[MeSH Terms] OR "Female"[Mesh] OR "Male"[Mesh] OR "Age Factors"[MeSH] OR "Aged"[MeSH] OR "Geriatrics"[Mesh])	Humans<br>English<br>2012-end
	**11**	**Is there a specific coagulation disorder associated with traumatic injury?**	
		“Wounds and Injuries”[MeSH] AND “Hemorrhage”[MeSH] AND “Hemostasis”[MeSH] AND "Blood Coagulation Disorders"[Mesh]	Humans<br>English
	**12**	**Should ventilation strategy be targeted to maximize oxygenation?**	
		("Craniocerebral Trauma"[MeSH] OR "Cerebrovascular Trauma"[MeSH] OR "Multiple Trauma"[MeSH] OR "Trauma, Nervous System"[MeSH] OR "Head Injuries, Penetrating"[MeSH] OR "Carotid Artery Injuries"[MeSH] OR "Head Injuries, Closed"[MeSH] OR "Spinal Cord Injuries"[MeSH] OR "Traumatology"[MeSH] OR "Brain Injuries"[MeSH]) AND ("Extracorporeal Membrane Oxygenation"[Mesh] OR "Respiration, Artificial"[Mesh] OR "Positive-Pressure Respiration"[Mesh] OR "Pulmonary Ventilation"[Mesh] OR "Intermittent Positive-Pressure Ventilation"[Mesh] OR "Continuous Positive Airway Pressure"[Mesh])	Humans<br>English<br>2012-end
	**13**	**Is tracheal intubation required in all patients?**	
	**14**	**Is there a rationale to support a delay in tracheal intubation?**	

		("Emergencies"[MeSH] OR "Emergency Treatment"[MeSH] OR "Emergency Medicine"[MeSH] OR "Emergency Medical Technicians"[MeSH] OR "Wounds and Injuries"[MeSH] OR "Craniocerebral Trauma"[MeSH] OR "Cerebrovascular Trauma"[MeSH] OR "Multiple Trauma"[MeSH] OR "Trauma, Nervous System"[MeSH] OR "Head Injuries, Penetrating"[MeSH] OR "Carotid Artery Injuries"[MeSH] OR "Head Injuries, Closed"[MeSH] OR "Spinal Cord Injuries"[MeSH] OR "Traumatology"[MeSH] OR "Brain Injuries"[MeSH]) AND "Intubation, Intratracheal"[Mesh] AND (“timing”[All Fields] OR “early”[All Fields] OR “late”[All Fields] OR “delay”[All Fields] OR “defer”[All Fields])	Humans, English, 2012-end/
	**15**	**Does the type of injury influence the selection of the hospital?**	
		("Trauma Severity Indices"[Mesh]) AND "Trauma Centers"[Mesh]) AND "Wounds and Injuries"[Mesh] OR "Regional Medical Programs"[Mesh])	Humans, English, Systematic Review
**II**		**DIAGNOSIS AND MONITORING OF BLEEDING**	
	**1**	**Is there evidence to support a correlation between the mechanism of injury and the risk of bleeding?**	
		“Wounds and Injuries”[MAJR] AND “Hemorrhage”[MeSH] AND correlation[All Fields]	Humans English 2012-end
	**2**	**Which clinical signs are most appropriate to detect the patient who is actively bleeding?**	
		"Wounds and Injuries"[MeSH] AND "Hemorrhage"[MeSH] AND ("Emergencies"[MeSH] OR "Emergency Treatment"[MeSH] OR "Emergency Medicine"[MeSH] OR "Emergency Medical Technicians"[MeSH]) AND ("Diagnosis"[MeSH] OR "Signs and Symptoms"[MeSH])	Humans English 2012-end
	**3**	**Which laboratory parameters (biochemical tests) are most appropriate to detect the patient who is actively bleeding?**	
		"Wounds and Injuries" [MeSH] AND "Hemorrhage" [MeSH] AND ("Clinical Chemistry Tests"[MeSH] OR "Monitoring, Physiologic" [MeSH] OR "Chemistry, Clinical"[MeSH] OR "Biological Markers"[MeSH] OR "Intercellular Signaling Peptides and Proteins"[MeSH])	Humans English 2012-end
	**4**	**Which imaging diagnostic tools are most appropriate to detect the patient who is actively bleeding?**	
		"Wounds and injuries"[MeSH] AND "Hemorrhage"[MeSH] AND "Diagnostic Imaging"[MeSH] AND ("Emergencies"[MeSH] OR "Emergency Treatment"[MeSH] OR "Emergency Medicine"[MeSH] OR "Emergency Medical Technicians"[MeSH])	Humans English 2012-end
	**5**	**Is there evidence to support the use of a specific score to assess the extent of bleeding?**	
	**6**	**Is the ATLS shock classification still a valid classification system?**	
		"Hemorrhage"[MeSH] AND "Trauma Severity Indices"[MeSH] AND "Wounds and Injuries"[MeSH]	Humans English 2012-end
	**7**	**Which coagulation monitoring tools are most appropriate to detect the patient who is actively bleeding?**	
	a	("Wounds and Injuries/diagnosis"[MAJR] OR "Hemorrhage/diagnosis"[MAJR] OR "Blood Coagulation Disorders/diagnosis"[MAJR] OR "Blood Coagulation Factors/analysis"[MAJR] OR "Blood Coagulation Factors/diagnostic use"[MAJR]) AND ("Point-of-Care Systems"[Mesh] OR "Thrombelastography"[Mesh])	Humans English 2012-end

	b	("Wounds and Injuries/diagnosis"[MAJR] AND "Hemorrhage/diagnosis"[MAJR]) AND ("Tomography, X-Ray"[Mesh] OR "Tomography, X-Ray Computed"[Mesh] OR "Multidetector Computed Tomography"[Mesh] OR "Ultrasonography"[Mesh] OR "Peritoneal Lavage"[Mesh])	Humans<br>English<br>2012-end
	**8**	**Which coagulation monitoring tools are most appropriate to detect post-traumatic coagulopathy?**	
		"Wounds and Injuries"[MeSH] AND ("Hemorrhage"[Mesh] OR "Disseminated Intravascular Coagulation"[Mesh]) AND ("Emergencies"[MeSH] OR "Emergency Treatment"[MeSH] OR "Emergency Medicine"[MeSH] OR "Emergency Medical Technicians"[MeSH]) AND ("Diagnosis"[MeSH] OR "Monitoring, Physiologic"[MeSH] OR "Point-of-Care Systems"[Mesh] OR "Thrombelastography"[Mesh] OR "Predictive Value of Tests"[Mesh])	Humans<br>English<br>2012-end
	**9**	**Does the type of monitoring tool used have an impact on outcome?**	
		"Wounds and Injuries"[MeSH] AND ("Hemorrhage"[Mesh] OR "Disseminated Intravascular Coagulation"[Mesh]) AND ("Emergencies"[MeSH] OR "Emergency Treatment"[MeSH] OR "Emergency Medicine"[MeSH] OR "Emergency Medical Technicians"[MeSH]) AND "Outcome Assessment (Health Care)"[Mesh] AND ("Pathological Conditions, Signs and Symptoms"[Mesh] OR "Diagnosis"[MeSH] OR "Point-of-Care Systems"[Mesh] OR "Thrombelastography"[Mesh] OR "Predictive Value of Tests"[Mesh])	Humans<br>English<br>2012-end
	**10**	**Using which coagulation monitoring tools can the effectiveness of therapeutic measures be monitored?**	
	**11**	**Is there any advantage to thrombelastometry performed at the bedside?**	
	**12**	**Is there any advantage to thrombelastometry performed in the central laboratory?**	
		"Wounds and Injuries"[MeSH] AND (("Blood Coagulation"[Mesh] AND "Blood Coagulation Disorders"[Mesh]) OR "Hemorrhage "[MeSH] OR "Shock, Hemorrhagic"[MeSH]) AND "Treatment Outcome"[Mesh] AND "Therapeutics"[Mesh] AND ("Emergencies"[MeSH] OR "Emergency Treatment"[MeSH] OR "Emergency Medicine"[MeSH] OR "Emergency Medical Technicians"[MeSH]) AND ("Diagnosis"[MeSH] OR "Monitoring, Physiologic" [MeSH] OR "Point-of-Care Systems"[Mesh] OR "Thrombelastography"[Mesh])	Humans<br>English<br>2012-end
	**13**	**Do early detection or diagnostic assessment of coagulation status influence outcome?**	
		"Wounds and Injuries"[MeSH] AND ("Hemorrhage"[Mesh] OR "Disseminated Intravascular Coagulation"[Mesh]) AND ("Emergencies"[MeSH] OR "Emergency Treatment"[MeSH] OR "Emergency Medicine"[MeSH] OR "Emergency Medical Technicians"[MeSH]) AND ("Diagnosis"[MeSH] OR "Monitoring, Physiologic"[MeSH] OR "Point-of-Care Systems"[Mesh] OR "Thrombelastography"[Mesh]) AND ("Early Medical Intervention"[Mesh] OR "Early Diagnosis"[Mesh])	Humans<br>English
	**14**	**Which type of intervention should patients with abdominal and/or thoracic bleeding receive?**	
		("Wounds and Injuries"[MeSH] OR "Emergencies"[MeSH] OR "Emergency Treatment"[MeSH] OR "Emergency Medicine"[MeSH] OR "Emergency Medical Technicians"[MeSH]) AND ("Hemorrhage"[Mesh] OR "Disseminated Intravascular Coagulation"[Mesh]) AND ("Thorax"[Mesh] OR "Abdomen"[Mesh] OR "Abdominal Cavity"[Mesh])	Humans<br>English<br>2012-end
	**15**	**Does the use of 'hybrid' shock/resuscitation/operating theatre facilities improve outcome in polytrauma patients?**	
		("Emergencies"[MeSH] OR "Emergency Treatment"[MeSH] OR "Emergency Medicine"[MeSH] OR "Emergency Medical Technicians"[MeSH] OR "Wounds and Injuries"[MeSH] OR "Craniocerebral Trauma"[MeSH] OR "Cerebrovascular Trauma"[MeSH] OR "Multiple Trauma"[MeSH] OR "Trauma, Nervous System"[MeSH] OR "Head Injuries, Penetrating"[MeSH] OR "Carotid Artery Injuries"[MeSH] OR "Head Injuries, Closed"[MeSH] OR "Spinal Cord Injuries"[MeSH] OR "Traumatology"[MeSH] OR "Brain Injuries"[MeSH]) AND ("Health Facility Size"[Mesh] OR "Facility Design and Construction"[Mesh])	Humans<br>English<br>2012-end
**III**		**TISSUE OXYGENATION, TYPE OF FLUID AND TEMPERATURE MANAGEMENT**	
	**1**	**What is the corridor for the haematocrit to be achieved for adequate tissue oxygenation?**	

		"Hematocrit"[MeSH] AND ("Oxygen Consumption"[MeSH] OR "Blood Gas Monitoring, Transcutaneous"[MeSH] OR "Blood Gas Analysis"[MeSH] OR "Gases"[MeSH] OR "Cell Respiration"[MeSH] OR "Blood Substitutes"[MeSH] OR "Blood Chemical Analysis"[MeSH] OR "Respiratory Function Tests"[MeSH] OR "Oximetry"[MeSH] OR "Blood Pressure"[MeSH] OR "Venous Pressure"[MeSH] OR "Hypotension"[MeSH]) AND ("Emergencies"[MeSH] OR "Emergency Treatment"[MeSH] OR "Emergency Medicine"[MeSH] OR "Emergency Medical Technicians"[MeSH] OR "Wounds and Injuries"[MeSH] OR "Craniocerebral Trauma"[MeSH] OR "Cerebrovascular Trauma"[MeSH] OR "Multiple Trauma"[MeSH] OR "Trauma, Nervous System"[MeSH] OR "Head Injuries, Penetrating"[MeSH] OR "Carotid Artery Injuries"[MeSH] OR "Head Injuries, Closed"[MeSH] OR "Spinal Cord Injuries"[MeSH] OR "Traumatology"[MeSH] OR "Brain Injuries"[MeSH])	Humans<br>English<br>2012-end
	**2**	**How should volume loading be managed?**	
		("Blood Substitutes"[MeSH] OR "Fluorocarbons"[MeSH] OR "Plasma Substitutes"[MeSH] OR "Fluorocarbons"[MeSH] OR "Plasma Substitutes"[MeSH] OR "Fluid Therapy"[MeSH] OR "Rehydration Solutions"[MeSH] OR "Solutions"[MeSH] OR "Colloids"[MeSH] OR "Sodium Chloride"[MeSH] OR "Saline Solution, Hypertonic"[MeSH]) AND ("Wounds and Injuries"[MeSH] OR "Craniocerebral Trauma"[MeSH] OR "Cerebrovascular Trauma"[MeSH] OR "Multiple Trauma"[MeSH] OR "Trauma, Nervous System"[MeSH] OR "Head Injuries, Penetrating"[MeSH] OR "Carotid Artery Injuries"[MeSH] OR "Head Injuries, Closed"[MeSH] OR "Spinal Cord Injuries"[MeSH] OR "Traumatology"[MeSH] OR "Brain Injuries"[MeSH]) AND ("Infusions, Parenteral"[MeSH] OR "Infusions, Intravenous"[MeSH])	Humans<br>English<br>2012-end
	**3**	**Does the blood pressure achieved during initial resuscitation influence morbidity or outcome in the trauma patient?**	
		("Blood Pressure Determination"[MeSH] OR "Blood Pressure"[MeSH] OR "Blood Pressure Monitoring, Ambulatory"[MeSH] OR "Hypertension"[MeSH] OR "Venous Pressure"[MeSH] OR "Hypotension"[MeSH]) AND ("Wounds and Injuries"[MeSH] OR "Craniocerebral Trauma"[MeSH] OR "Cerebrovascular Trauma"[MeSH] OR "Multiple Trauma"[MeSH] OR "Trauma, Nervous System"[MeSH] OR "Head Injuries, Penetrating"[MeSH] OR "Carotid Artery Injuries"[MeSH] OR "Head Injuries, Closed"[MeSH] OR "Spinal Cord Injuries"[MeSH] OR "Traumatology"[MeSH] OR "Brain Injuries"[MeSH]) AND ("Emergencies"[MeSh] OR "Emergency Treatment"[MeSH] OR "Emergency Medicine"[MeSh] OR "Emergency Medical Technicians"[MeSH])	Humans<br>English<br>2012-end
	**4**	**Does the type of fluid used for initial resuscitation influence morbidity or outcome in the trauma patient?**	
		("Blood Substitutes"[MeSH] OR "Fluorocarbons"[MeSH] OR "Plasma Substitutes"[MeSH] OR "Fluorocarbons"[MeSH] OR "Plasma Substitutes"[MeSH] OR "Fluid Therapy"[MeSH] OR "Rehydration Solutions"[MeSH] OR "Solutions"[MeSH] OR "Colloids"[MeSH] OR "Sodium Chloride"[MeSH] OR "Saline Solution, Hypertonic"[MeSH]) AND ("Wounds and Injuries"[MeSH] OR "Craniocerebral Trauma"[MeSH] OR "Cerebrovascular Trauma"[MeSH] OR "Multiple Trauma"[MeSH] OR "Trauma, Nervous System"[MeSH] OR "Head Injuries, Penetrating"[MeSH] OR "Carotid Artery Injuries"[MeSH] OR "Head Injuries, Closed"[MeSH] OR "Spinal Cord Injuries"[MeSH] OR "Traumatology"[MeSH] OR "Brain Injuries"[MeSH]) AND ("Emergencies"[MeSH] OR "Emergency Treatment"[MeSH] OR "Emergency Medicine"[MeSH] OR "Emergency Medical Technicians"[MeSH])	Humans<br>English<br>2012-end
	**5**	**Does controlled mild hypothermia (34°C) affect outcome or morbidity in the trauma patient compared to normothermia?**	
		("Hypothermia"[MeSH] OR "Gastric Hypothermia"[MeSH] OR "Hypothermia, Induced"[MeSH] OR "Circulatory Arrest, Deep Hypothermia Induced"[MeSH]) AND "Outcome and Process Assessment (Health Care)"[MeSH] AND ("Wounds and Injuries"[MeSH] OR "injuries"[Subheading] OR "Craniocerebral Trauma"[MeSH] OR "Trauma Severity Indices"[MeSH] OR "Trauma Centers"[MeSH] OR "Cumulative Trauma Disorders"[MeSH] OR "Cerebrovascular Trauma"[MeSH] OR "Multiple Trauma"[MeSH] OR "Trauma, Nervous System"[MeSH] OR "Head Injuries, Penetrating"[MeSH] OR "Abducens Nerve Injury"[MeSH] OR "Optic Nerve Injuries"[MeSH] OR "Coma, Post-Head Injury"[MeSH] OR "Facial Nerve Injuries"[MeSH] OR "Carotid Artery Injuries"[MeSH] OR "Head Injuries, Closed"[MeSH] OR "Spinal Cord Injuries"[MeSH] OR "Traumatology"[MeSH] OR "Brain Injuries"[MeSH])	Humans<br>English<br>2012-end
	**6**	**Which types of colloids are appropriate for use under which circumstances in the bleeding trauma patient?**	

		("Wounds and Injuries"[MeSH] OR "Craniocerebral Trauma"[MeSH] OR "Cerebrovascular Trauma"[MeSH] OR "Multiple Trauma"[MeSH] OR "Trauma, Nervous System"[MeSH] OR "Head Injuries, Penetrating"[MeSH] OR "Carotid Artery Injuries"[MeSH] OR "Head Injuries, Closed"[MeSH] OR "Spinal Cord Injuries"[MeSH] OR "Traumatology"[MeSH] OR "Brain Injuries"[MeSH]) AND ("Emergencies"[MeSH] OR "Emergency Treatment"[MeSH] OR "Emergency Medicine"[MeSH] OR "Emergency Medical Technicians"[MeSH]) AND "Colloids"[MeSH]	Humans<br>English
	**7**	**Which types of crystalloids are appropriate for use under which circumstances in the bleeding trauma patient?**	
	**8**	**Is the use of Ringer's lactate appropriate in patients with severe head injury?**	
		("Wounds and Injuries"[MeSH] OR "Craniocerebral Trauma"[MeSH] OR "Cerebrovascular Trauma"[MeSH] OR "Multiple Trauma"[MeSH] OR "Trauma, Nervous System"[MeSH] OR "Head Injuries, Penetrating"[MeSH] OR "Carotid Artery Injuries"[MeSH] OR "Head Injuries, Closed"[MeSH] OR "Spinal Cord Injuries"[MeSH] OR "Traumatology"[MeSH] OR "Brain Injuries"[MeSH]) AND ("Emergencies"[MeSH] OR "Emergency Treatment"[MeSH] OR "Emergency Medicine"[MeSH] OR "Emergency Medical Technicians"[MeSH]) AND ("Sodium Chloride"[MeSH] OR "Saline Solution, Hypertonic"[MeSH])	Humans<br>English
	**9**	**Is there an appropriate use of vasopressors or inotropic agents in the bleeding trauma patient?**	
	**10**	**Are vasopressors or inotropic agents harmful in the bleeding trauma patient?**	
		("Emergencies"[MAJR] OR "Emergency Treatment"[MAJR] OR "Emergency Medicine"[MAJR] OR "Emergency Medical Technicians"[MAJR] OR "Wounds and Injuries"[MAJR] OR "Craniocerebral Trauma"[MAJR] OR "Cerebrovascular Trauma"[MAJR] OR "Multiple Trauma"[MAJR] OR "Trauma, Nervous System"[MAJR] OR "Head Injuries, Penetrating"[MAJR] OR "Carotid Artery Injuries"[MAJR] OR "Head Injuries, Closed"[MAJR] OR "Spinal Cord Injuries"[MAJR] OR "Traumatology"[MAJR] OR "Brain Injuries"[MAJR]) AND ("Vasoconstrictor Agents"[Mesh] OR "Cardiotonic Agents"[MeSH])	Humans<br>English<br>10 years
	**11**	**What is the indication for the use of RBC in bleeding after trauma?**	
	**12**	**Does administration of RBCs affect clinical outcome in bleeding patients?**	
	**13**	**What are the risks of giving RBCs?**	
	**14**	**How many RBCs are enough?**	
		("Wounds and Injuries"[MeSH] OR "Craniocerebral Trauma"[MeSH] OR "Cerebrovascular Trauma"[MeSH] OR "Multiple Trauma"[MeSH] OR "Trauma, Nervous System"[MeSH] OR "Head Injuries, Penetrating"[MeSH] OR "Carotid Artery Injuries"[MeSH] OR "Head Injuries, Closed"[MeSH] OR "Spinal Cord Injuries"[MeSH] OR "Traumatology"[MeSH] OR "Brain Injuries"[MeSH]) AND ("Erythrocytes"[MeSH] OR "Erythrocyte Transfusion"[MeSH])	Humans<br>English<br>2012-end
	**15**	**What is the effect of RBC transfusion on microcirculation?**	
		("Wounds and Injuries"[MeSH] OR "Craniocerebral Trauma"[MeSH] OR "Cerebrovascular Trauma"[MeSH] OR "Multiple Trauma"[MeSH] OR "Trauma, Nervous System"[MeSH] OR "Head Injuries, Penetrating"[MeSH] OR "Carotid Artery Injuries"[MeSH] OR "Head Injuries, Closed"[MeSH] OR "Spinal Cord Injuries"[MeSH] OR "Traumatology"[MeSH] OR "Brain Injuries"[MeSH]) AND ("Erythrocytes"[MeSH] OR "Erythrocyte Transfusion"[MeSH]) AND "Microcirculation"[Mesh]	Humans<br>English
	**16**	**Does the duration of RBC storage affect clinical outcome?**	
		("Wounds and Injuries"[MeSH] OR "Craniocerebral Trauma"[MeSH] OR "Cerebrovascular Trauma"[MeSH] OR "Multiple Trauma"[MeSH] OR "Trauma, Nervous System"[MeSH] OR "Head Injuries, Penetrating"[MeSH] OR "Carotid Artery Injuries"[MeSH] OR "Head Injuries, Closed"[MeSH] OR "Spinal Cord Injuries"[MeSH] OR "Traumatology"[MeSH] OR "Brain Injuries"[MeSH]) AND ("Erythrocytes"[MeSH] OR "Erythrocyte Transfusion"[MeSH]) AND "Blood Preservation"[Mesh]	Humans<br>English
	**17**	**What is the appropriate use of epoetin in post-traumatic anaemia?**	

		("Wounds and Injuries"[MeSH] OR "Craniocerebral Trauma"[MeSH] OR "Cerebrovascular Trauma"[MeSH] OR "Multiple Trauma"[MeSH] OR "Trauma, Nervous System"[MeSH] OR "Head Injuries, Penetrating"[MeSH] OR "Carotid Artery Injuries"[MeSH] OR "Head Injuries, Closed"[MeSH] OR "Spinal Cord Injuries"[MeSH] OR "Traumatology"[MeSH] OR "Brain Injuries"[MeSH]) AND ("Erythrocytes"[MeSH] OR "Erythrocyte Transfusion"[MeSH] OR "Anemia"[Mesh]) AND "Erythropoietin"[Mesh]	Humans<br>English
	**18**	**Is there a role for iron therapy in the treatment of post-traumatic anaemia?**	
		("Wounds and Injuries"[MeSH] OR "Craniocerebral Trauma"[MeSH] OR "Cerebrovascular Trauma"[MeSH] OR "Multiple Trauma"[MeSH] OR "Trauma, Nervous System"[MeSH] OR "Head Injuries, Penetrating"[MeSH] OR "Carotid Artery Injuries"[MeSH] OR "Head Injuries, Closed"[MeSH] OR "Spinal Cord Injuries"[MeSH] OR "Traumatology"[MeSH] OR "Brain Injuries"[MeSH]) AND ("Erythrocytes"[MeSH] OR "Erythrocyte Transfusion"[MeSH] OR "Anemia"[Mesh]) AND "Iron"[Mesh]	Humans<br>English
	**19**	**Should treatment target cardiac output?**	
		("Oxygen Consumption"[MeSH] OR "Blood Gas Monitoring, Transcutaneous"[MeSH] OR "Blood Gas Analysis"[MeSH] OR "Gases"[MeSH] OR "Cell Respiration"[MeSH] OR "Blood Substitutes"[MeSH] OR "Blood Chemical Analysis"[MeSH] OR "Respiratory Function Tests"[MeSH] OR "Oximetry"[MeSH] OR "Blood Pressure"[MeSH] OR "Venous Pressure"[MeSH] OR "Hypotension"[MeSH]) AND ("Emergencies"[MeSH] OR "Emergency Treatment"[MeSH] OR "Emergency Medicine"[MeSH] OR "Emergency Medical Technicians"[MeSH] OR "Wounds and Injuries"[MeSH] OR "Craniocerebral Trauma"[MeSH] OR "Cerebrovascular Trauma"[MeSH] OR "Multiple Trauma"[MeSH] OR "Trauma, Nervous System"[MeSH] OR "Head Injuries, Penetrating"[MeSH] OR "Carotid Artery Injuries"[MeSH] OR "Head Injuries, Closed"[MeSH] OR "Spinal Cord Injuries"[MeSH] OR "Traumatology"[MeSH] OR "Brain Injuries"[MeSH]) AND "Cardiac Output"[Mesh]	Humans<br>English<br>10 years
	**20**	**What is the effect of different infusion fluids (colloids, crystalloids) on coagulation?**	
		("Wounds and Injuries"[MeSH] OR "Craniocerebral Trauma"[MeSH] OR "Cerebrovascular Trauma"[MeSH] OR "Multiple Trauma"[MeSH] OR "Trauma, Nervous System"[MeSH] OR "Head Injuries, Penetrating"[MeSH] OR "Carotid Artery Injuries"[MeSH] OR "Head Injuries, Closed"[MeSH] OR "Spinal Cord Injuries"[MeSH] OR "Traumatology"[MeSH] OR "Brain Injuries"[MeSH]) AND ("Hemorrhage"[Mesh] OR "Disseminated Intravascular Coagulation"[Mesh]) AND ("Colloids"[MeSH] OR "Sodium Chloride"[MeSH] OR "Saline Solution, Hypertonic"[MeSH])	Humans<br>English<br>10 years
	**21**	**Is there a role for hypertonic solutions in polytrauma patients?**	
	**22**	**Is there a role for hypertonic solutions in patients with traumatic brain injury?**	
		("Wounds and Injuries"[MeSH] OR "Craniocerebral Trauma"[MeSH] OR "Cerebrovascular Trauma"[MeSH] OR "Multiple Trauma"[MeSH] OR "Trauma, Nervous System"[MeSH] OR "Head Injuries, Penetrating"[MeSH] OR "Carotid Artery Injuries"[MeSH] OR "Head Injuries, Closed"[MeSH] OR "Spinal Cord Injuries"[MeSH] OR "Traumatology"[MeSH] OR "Brain Injuries"[MeSH]) AND ("Hemorrhage"[Mesh] OR "Disseminated Intravascular Coagulation"[Mesh]) AND "Hypertonic Solutions"[Mesh]	Humans<br>English<br>10 years
	**23**	**Does the age of the patient affect clinical outcome?**	
		("Wounds and Injuries"[MeSH] OR "Craniocerebral Trauma"[MeSH] OR "Cerebrovascular Trauma"[MeSH] OR "Multiple Trauma"[MeSH] OR "Trauma, Nervous System"[MeSH] OR "Head Injuries, Penetrating"[MeSH] OR "Carotid Artery Injuries"[MeSH] OR "Head Injuries, Closed"[MeSH] OR "Spinal Cord Injuries"[MeSH] OR "Traumatology"[MeSH] OR "Brain Injuries"[MeSH]) AND ("Erythrocytes"[MeSH] OR "Erythrocyte Transfusion"[MeSH]) AND ("Age Factors"[MeSH] OR "Aged"[MeSH] OR "Geriatrics"[Mesh])	Humans<br>English<br>10 years
	**24**	**Does the minimisation of blood sampling volume and frequency improve outcome?**	
		("Hemorrhage"[Mesh] OR "Disseminated Intravascular Coagulation"[Mesh]) AND "Blood Specimen Collection"[Mesh]	Humans<br>English<br>10 years
**IV**		**RAPID CONTROL OF BLEEDING**	

The European guideline on management of major bleeding and coagulopathy following trauma: Fourth edition

Rossaint R, Bouillon B, Cerny V, Coats TJ, Duranteau J, Fernández-Mondéjar E, Filipescu D, Hunt BJ, Komadina R, Nardi G, Neugebauer EAM, Ozier Y, Riddez L, Schultz A, Vincent J-L, Spahn DR

	**1**	**Can the mechanism of injury (e.g. blunt vs. penetrating trauma) be used as a determinant for deciding which patients in haemorrhagic shock are candidates for surgical bleeding control?**	
		"Wounds and Injuries"[MAJR] AND "Hemorrhage"[MeSH] AND "Shock, Hemorrhagic"[MeSH] AND ("Emergencies"[MeSH] OR "Emergency Treatment"[MeSH] OR "Emergency Medicine"[MeSH] OR "Emergency Medical Technicians"[MeSH])	Humans<br>English<br>2012-end
	**2**	**Does angiographic embolisation improve the outcome of patients with haemorrhagic shock and pelvic ring disruption?**	
		"Embolization, Therapeutic"[MeSH] AND ("Shock, Hemorrhagic"[MeSH] OR "Fractures"[MeSH])	Humans<br>English<br>2012-end
	**3**	**What are the characteristics of patients with free intraabdominal fluid according to FAST in whom secondary diagnostics (i.e. CT scan) can be safely performed?**	
		"Wounds and Injuries"[MeSH] AND ("Diagnostic Imaging"[MeSH] OR "Ultrasonography"[MeSH]) AND "Ascitic Fluid"[MeSH]	Humans<br>English<br>2012-end
	**4**	**What characterises the patient in heamorrhagic shock in whom immediate aortic cross-clamping is warranted?**	
	**5**	**Does use of an intra-aortic balloon catheter reduce bleeding or improve clinical outcomes?**	
		"Wounds and Injuries"[MAJR] AND ("Hemorrhage"[MeSH] OR "Shock, Hemorrhagic"[MeSH]) AND ("Emergencies"[MeSH] OR "Emergency Treatment"[MeSH] OR "Emergency Medicine"[MeSH] OR "Emergency Medical Technicians"[MeSH]) AND ("Angioscopy"[MeSH] OR "Heart-Assist Devices"[Mesh] OR "Cardiovascular Surgical Procedures"[MeSH] OR "Minimally Invasive Surgical Procedures"[Mesh] OR "Intra-Aortic Balloon Pumping"[Mesh])	Humans<br>English<br>10 years
	**6**	**Does the elapsed time from admission to OR influence outcome for trauma patients who need emergency surgery?**	
		("Emergency Medical Services"[MeSH] OR "Emergencies"[MeSH] OR "Emergency Treatment"[MeSH] OR "Critical Care"[MeSH]) AND "Hemorrhage"[MeSH] AND "Outcome and Process Assessment (Health Care)"[MeSH] AND ("Wounds and Injuries"[MeSH] OR "injuries"[Subheading] OR "Craniocerebral Trauma"[MeSH] OR "Trauma Severity Indices"[MeSH] OR "Trauma Centers"[MeSH] OR "Cumulative Trauma Disorders"[MeSH] OR "Cerebrovascular Trauma"[MeSH] OR "Multiple Trauma"[MeSH] OR "Trauma, Nervous System"[MeSH] OR "Head Injuries, Penetrating"[MeSH] OR "Abducens Nerve Injury"[MeSH] OR "Optic Nerve Injuries"[MeSH] OR "Coma, Post-Head Injury"[MeSH] OR "Facial Nerve Injuries"[MeSH] OR "Carotid Artery Injuries"[MeSH] OR "Head Injuries, Closed"[MeSH] OR "Spinal Cord Injuries"[MeSH] OR "Traumatology"[MeSH] OR "Brain Injuries"[MeSH])	Humans<br>English<br>2012-end
	**7**	**Does urgent surgery to control haemorrhage improve the outcome of patients with haemorrhagic shock and pelvic ring disruption?**	
		"Hip Fractures"[MeSH] AND "Shock, Hemorrhagic"[MeSH]	Humans<br>English<br>2012-end
	**8**	**What characterises the patient with free intraabdominal fluid according to FAST who requires immediate laparotomy?**	
		"Ascitic Fluid"[MeSH] AND "Laparotomy"[MeSH] AND ("Wounds and Injuries"[MeSH] OR "injuries"[Subheading] OR "Craniocerebral Trauma"[MeSH] OR "Trauma Severity Indices"[MeSH] OR "Trauma Centers"[MeSH] OR "Cumulative Trauma Disorders"[MeSH] OR "Cerebrovascular Trauma"[MeSH] OR "Multiple Trauma"[MeSH] OR "Trauma, Nervous System"[MeSH] OR "Head Injuries, Penetrating"[MeSH] OR "Abducens Nerve Injury"[MeSH] OR "Optic Nerve Injuries"[MeSH] OR "Coma, Post-Head Injury"[MeSH] OR "Facial Nerve Injuries"[MeSH] OR "Carotid Artery Injuries"[MeSH] OR "Head Injuries, Closed"[MeSH] OR "Spinal Cord Injuries"[MeSH] OR "Traumatology"[MeSH] OR "Brain Injuries"[MeSH])	Humans<br>English<br>2012-end
	**9**	**What characterises the patient in haemorrhagic shock in whom packing is warranted?**	

		"Wounds and Injuries"[MeSH] AND ("Ascitic Fluid"[MeSH] OR "Shock, Hemorrhagic"[MeSH] OR "Hemorrhage"[MeSH]) AND "Abdominal Injuries"[Mesh] AND ("Hemostasis, Surgical"[Mesh] OR "Hemostatic Techniques"[Mesh] OR "Surgical Procedures, Minimally Invasive"[Mesh])	Humans<br>English<br>2012-end
	**10**	**What characterises the patient in haemorrhagic shock in whom use of a tourniquet is warranted?**	
		"Wounds and Injuries"[MeSH] AND ("Hemorrhage"[MeSH] OR "Shock, Hemorrhagic"[MeSH]) AND "Tourniquets"[Mesh]	Humans<br>English<br>2012-end
	**11**	**Does the use of local haemostatic agents improve outcome of patients with haemorrhagic shock?**	
		"Wounds and Injuries"[MeSH] AND ("Hemorrhage"[MeSH] OR "Shock, Hemorrhagic"[MeSH]) AND "Hemostatic Techniques"[Mesh] AND ("Fibrin Tissue Adhesive"[Mesh] OR "Fibrin Foam"[Mesh] OR "Gelatin Sponge, Absorbable"[Mesh] OR "Thrombin"[Mesh] OR "Thromboplastin"[Mesh])	Humans<br>English<br>2012-end
	**12**	**Does urgent surgery to prevent further bleeding improve outcome for patients with haemorrhagic shock?**	
		"Wounds and Injuries"[MeSH] AND ("Hemorrhage"[MeSH] OR "Shock, Hemorrhagic"[MeSH]) AND "Hemostasis, Surgical"[Mesh]	Humans<br>English<br>2012-end
**V**		**INITIAL MANAGEMENT OF BLEEDING & COAGULOPATHY**	
		FFP	
	**1**	**What is the indication for giving FFP to patients bleeding after trauma?**	
	**2**	**Does administration of FFP reduce the severity of bleeding in patients with acquired coagulation factor deficits?**	
	**3**	**Does administration of FFP affect clinical outcome in bleeding patients with acquired coagulation factor deficits?**	
	**4**	**What are the risks of administration of FFP?**	
	**5**	**What is the recommended dose of FFP to be given?**	
		("Wounds and Injuries"[MeSH] OR "Craniocerebral Trauma"[MeSH] OR "Cerebrovascular Trauma"[MeSH] OR "Multiple Trauma"[MeSH] OR "Trauma, Nervous System"[MeSH] OR "Head Injuries, Penetrating"[MeSH] OR "Carotid Artery Injuries"[MeSH] OR "Head Injuries, Closed"[MeSH] OR "Spinal Cord Injuries"[MeSH] OR "Traumatology"[MeSH] OR "Brain Injuries"[MeSH]) AND ("Plasma"[MeSH] OR "Plasma Exchange"[MeSH])	Humans<br>English<br>2012-end
	**6**	**What is the recommended dose of FFP relative to the dose of RBCs administered?**	
	**7**	**Does the relative dose of FFP:RBCs have an impact on outcome?**	
	**8**	**Is there any benefit to a 1:1:1 transfusion regimen?**	
	**9**	**Is there any benefit to goal-directed coagulation management using factor replacement?**	
		("Emergencies"[MeSH] OR "Emergency Treatment"[MeSH] OR "Emergency Medicine"[MeSH] OR "Emergency Medical Technicians"[MeSH] OR "Wounds and Injuries"[MeSH] OR "Craniocerebral Trauma"[MeSH] OR "Cerebrovascular Trauma"[MeSH] OR "Multiple Trauma"[MeSH] OR "Trauma, Nervous System"[MeSH] OR "Head Injuries, Penetrating"[MeSH] OR "Carotid Artery Injuries"[MeSH] OR "Head Injuries, Closed"[MeSH] OR "Spinal Cord Injuries"[MeSH] OR "Traumatology"[MeSH] OR "Brain Injuries"[MeSH]) AND ("Erythrocytes"[MeSH] OR "Erythrocyte Transfusion"[MeSH]) AND ("Plasma"[MeSH] OR "Plasma Exchange"[MeSH] OR "Blood Platelets"[MeSH] OR "Thrombocytopenia"[MeSH])	Humans<br>English
		Anti-fibrinolytic agents	

	1	**What are the indications for anti-fibrinolytics in a bleeding patient?**	
	2	**What are the recommended doses of anti-fibrinolytic agents in bleeding patients?**	
	3	**Have anti-fibrinolytics been shown to be efficacious?**	
	4	**What are the risks of using anti-fibrinolytics in bleeding patients?**	
	5	**Which anti-fibrinolytic(s) are preferable based on comparative data and risk benefit analysis?**	
	6	**Which antifibrinolytic agent(s) are appropriate for use in the bleeding trauma patient?**	
	7	**Is the use of antifibrinolytic agents appropriate in all cases of suspected significant bleeding?**	
	8	**Does the timing of antifibrinolytic treatment affect outcome?**	
		"Antifibrinolytic Agents"[MeSH] AND ("Hemorrhage"[MeSH] OR "Brain Stem Hemorrhage, Traumatic"[MeSH] OR "Subarachnoid Hemorrhage"[MeSH] OR "Cerebral Hemorrhage"[MeSH] OR "Cerebral Hemorrhage, Traumatic"[MeSH] OR "Subarachnoid Hemorrhage, Traumatic"[MeSH] OR "Brain Hemorrhage, Traumatic"[MeSH] OR "Intracranial Hemorrhage, Traumatic"[MeSH]) NOT "Aprotinin"[Mesh]	Humans English 2012-end
	9	**Is the use of antifibrinolytic agents cost effective?**	
		("Hemorrhage"[MeSH] OR "Brain Stem Hemorrhage, Traumatic"[MeSH] OR "Subarachnoid Hemorrhage"[MeSH] OR "Cerebral Hemorrhage"[MeSH] OR "Cerebral Hemorrhage, Traumatic"[MeSH] OR "Subarachnoid Hemorrhage, Traumatic"[MeSH] OR "Brain Hemorrhage, Traumatic"[MeSH] OR "Intracranial Hemorrhage, Traumatic"[MeSH]) AND "Antifibrinolytic Agents"[MeSH] NOT "Aprotinin"[Mesh] AND ("Costs and Cost Analysis"[Mesh] OR "Cost-Benefit Analysis"[Mesh] OR "Cost of Illness"[Mesh] OR "Cost Savings"[Mesh] OR "Health Care Costs"[Mesh] OR "Hospital Costs"[Mesh] OR "Drug Costs"[Mesh])	Humans English
		Coagulation factor concentrates	
	1	**What are the indications for the use of coagulation factor concentrates in a bleeding patient?**	
	2	**What are the recommended doses of coagulation factor concentrates in a bleeding patient?**	
	3	**Have coagulation factor concentrates been shown to be efficacious?**	
	4	**What are the risks of using coagulation factor concentrates in bleeding patients?**	
		("Emergencies"[MeSH] OR "Emergency Treatment"[MeSH] OR "Emergency Medicine"[MeSH] OR "Emergency Medical Technicians"[MeSH] OR "Wounds and Injuries"[MeSH] OR "Craniocerebral Trauma"[MeSH] OR "Cerebrovascular Trauma"[MeSH] OR "Multiple Trauma"[MeSH] OR "Trauma, Nervous System"[MeSH] OR "Head Injuries, Penetrating"[MeSH] OR "Carotid Artery Injuries"[MeSH] OR "Head Injuries, Closed"[MeSH] OR "Spinal Cord Injuries"[MeSH] OR "Traumatology"[MeSH] OR "Brain Injuries"[MeSH]) AND ("Factor XIII"[Mesh] OR "recombinant FVIIa" [Supplementary Concept])	Humans English 2012-end
		Antithrombotic management	
	1	**What type of antithrombotic therapy is appropriate following traumatic injury?**	
	2	**How can the effect of antithrombotic agents be antagonised if necessary to support coagulation?**	
		("Emergencies"[MAJR] OR "Emergency Treatment"[MAJR] OR "Emergency Medicine"[MAJR] OR "Emergency Medical Technicians"[MAJR] OR "Wounds and Injuries"[MAJR] OR "Craniocerebral Trauma"[MAJR] OR "Cerebrovascular Trauma"[MAJR] OR "Multiple Trauma"[MAJR] OR "Trauma, Nervous System"[MAJR] OR "Head Injuries, Penetrating"[MAJR] OR "Carotid Artery Injuries"[MAJR] OR "Head Injuries, Closed"[MAJR] OR "Spinal Cord Injuries"[MAJR] OR "Traumatology"[MAJR] OR "Brain Injuries"[MAJR]) AND ("Thrombosis/prevention and control"[Mesh] OR "Venous Thrombosis/prevention and control"[Mesh] OR "Pulmonary Embolism/prevention and control"[Mesh])	Humans English 2012-end
**VI**		**FURTHER RESUSCITATION**	

The European guideline on management of major bleeding and coagulopathy following trauma: Fourth edition

Rossaint R, Bouillon B, Cerny V, Coats TJ, Duranteau J, Fernández-Mondéjar E, Filipescu D, Hunt BJ, Komadina R, Nardi G, Neugebauer EAM, Ozier Y, Riddez L, Schultz A, Vincent J-L, Spahn DR

		Goal directed therapy	
	**1**	**Which coagulation monitoring tools can be used to support resuscitation?**	
		("Wounds and Injuries"[MeSH] OR "Hemorrhage"[Mesh]) AND ("Emergencies"[MeSH] OR "Emergency Treatment"[MeSH] OR "Emergency Medicine"[MeSH] OR "Emergency Medical Technicians"[MeSH]) AND ("Diagnosis"[MeSH] OR "Monitoring, Physiologic"[MeSH] OR "Point-of-Care Systems"[Mesh] OR "Thrombelastography"[Mesh] OR "Predictive Value of Tests"[Mesh]) AND "Resuscitation"[Mesh] AND "Blood Coagulation Tests"[Mesh]	Humans<br>English<br>10 years
		Platelets	
	**1**	**What is the indication for the use of platelets in bleeding after trauma?**	
	**2**	**Does administration of platelets reduce the severity of bleeding in patients with thrombocytopenia?**	
	**3**	**Does administration of platelets affect clinical outcome in bleeding patients with thrombocytopenia?**	
	**4**	**Does administration of platelets affect clinical outcome in bleeding patients without thrombocytopaenia?**	
	**5**	**What are the risks of giving platelets?**	
		("Blood Platelets"[MeSH] OR "Thrombocytopenia"[MeSH]) AND ("Wounds and Injuries"[MeSH] OR "Craniocerebral Trauma"[MeSH] OR "Cerebrovascular Trauma"[MeSH] OR "Multiple Trauma"[MeSH] OR "Trauma, Nervous System"[MeSH] OR "Head Injuries, Penetrating"[MeSH] OR "Carotid Artery Injuries"[MeSH] OR "Head Injuries, Closed"[MeSH] OR "Spinal Cord Injuries"[MeSH] OR "Traumatology"[MeSH] OR "Brain Injuries"[MeSH])	Humans<br>English<br>2012-end
	**6**	**Does the duration of platelet storage affect clinical outcome?**	
		("Wounds and Injuries"[MeSH] OR "Craniocerebral Trauma"[MeSH] OR "Cerebrovascular Trauma"[MeSH] OR "Multiple Trauma"[MeSH] OR "Trauma, Nervous System"[MeSH] OR "Head Injuries, Penetrating"[MeSH] OR "Carotid Artery Injuries"[MeSH] OR "Head Injuries, Closed"[MeSH] OR "Spinal Cord Injuries"[MeSH] OR "Traumatology"[MeSH] OR "Brain Injuries"[MeSH]) AND ("Blood Platelets"[MeSH] OR "Thrombocytopenia"[MeSH]) AND "Blood Preservation"[Mesh]	Humans<br>English
		Anti-platelet agents	
	**1**	**Does pre-medication with anti-platelet agents affect clinical outcome in bleeding patients with thrombocytopenia?**	
	**2**	**Does pre-medication with anti-platelet agents affect clinical outcome in bleeding patients who receive platelet therapy?**	
		("Wounds and Injuries"[MeSH] OR "Craniocerebral Trauma"[MeSH] OR "Cerebrovascular Trauma"[MeSH] OR "Multiple Trauma"[MeSH] OR "Trauma, Nervous System"[MeSH] OR "Head Injuries, Penetrating"[MeSH] OR "Carotid Artery Injuries"[MeSH] OR "Head Injuries, Closed"[MeSH] OR "Spinal Cord Injuries"[MeSH] OR "Traumatology"[MeSH] OR "Brain Injuries"[MeSH]) AND "Platelet Aggregation Inhibitors"[Mesh]	Humans<br>English<br>2012-end
	**3**	**What is the appropriate use of platelets in the treatment of patients pre-treated with anti-platelet agents?**	
		("Emergencies"[MAJR] OR "Emergency Treatment"[MAJR] OR "Emergency Medicine"[MAJR] OR "Emergency Medical Technicians"[MAJR] OR "Wounds and Injuries"[MAJR] OR "Craniocerebral Trauma"[MAJR] OR "Cerebrovascular Trauma"[MAJR] OR "Multiple Trauma"[MAJR] OR "Trauma, Nervous System"[MAJR] OR "Head Injuries, Penetrating"[MAJR] OR "Carotid Artery Injuries"[MAJR] OR "Head Injuries, Closed"[MAJR] OR "Spinal Cord Injuries"[MAJR] OR "Traumatology"[MAJR] OR "Brain Injuries"[MAJR]) AND "Platelet Aggregation Inhibitors"[Mesh] AND ("Therapeutics"[Mesh] OR "Emergency Treatment"[Mesh] OR "Blood Platelets"[MeSH])	Humans<br>English
		Fibrinogen and cryoprecipitate	
	**1**	**What are the indications for fibrinogen or cryoprecipitate?**	

The European guideline on management of major bleeding and coagulopathy following trauma: Fourth edition

Rossaint R, Bouillon B, Cerny V, Coats TJ, Duranteau J, Fernández-Mondéjar E, Filipescu D, Hunt BJ, Komadina R, Nardi G, Neugebauer EAM, Ozier Y, Riddez L, Schultz A, Vincent J-L, Spahn DR

	2	**What is the recommended cryoprecipitate dose in bleeding patients?**	
	3	**Have fibrinogen or cryoprecipitate been shown to be efficacious in bleeding patients?**	
	4	**What are the risks of using fibrinogen or cryoprecipitate in bleeding patients?**	
		("Wounds and Injuries"[MeSH] OR "Craniocerebral Trauma"[MeSH] OR "Cerebrovascular Trauma"[MeSH] OR "Multiple Trauma"[MeSH] OR "Trauma, Nervous System"[MeSH] OR "Head Injuries, Penetrating"[MeSH] OR "Carotid Artery Injuries"[MeSH] OR "Head Injuries, Closed"[MeSH] OR "Spinal Cord Injuries"[MeSH] OR "Traumatology"[MeSH] OR "Brain Injuries"[MeSH]) AND ("Fibrinogen"[MeSH] OR "cryoprecipitate coagulum"[Substance Name] OR "Nour-Eldin fraction"[Substance Name])	Humans<br>English<br>2012-end
	5	**What is the recommended target fibrinogen level?**	
	6	**Which coagulation monitoring tools are most appropriate to detect fibrinogen level?**	
		("Emergencies"[MeSH] OR "Emergency Treatment"[MeSH] OR "Emergency Medicine"[MeSH] OR "Emergency Medical Technicians"[MeSH] OR "Wounds and Injuries"[MeSH] OR "Craniocerebral Trauma"[MeSH] OR "Cerebrovascular Trauma"[MeSH] OR "Multiple Trauma"[MeSH] OR "Trauma, Nervous System"[MeSH] OR "Head Injuries, Penetrating"[MeSH] OR "Carotid Artery Injuries"[MeSH] OR "Head Injuries, Closed"[MeSH] OR "Spinal Cord Injuries"[MeSH] OR "Traumatology"[MeSH] OR "Brain Injuries"[MeSH]) AND ("Fibrinogen"[MeSH] OR "cryoprecipitate coagulum"[Substance Name] OR "Nour-Eldin fraction"[Substance Name]) AND ("administration and dosage "[Subheading] OR "Monitoring, Physiologic"[Mesh] OR "Biomarkers, Pharmacological"[Mesh] OR "Drug Monitoring"[Mesh] OR "Point-of-Care Systems"[Mesh] OR "Thrombelastography"[Mesh])	Humans<br>English<br>2012-end
		Prothrombin complex concentrates	
	1	**What is the indication for PCCs in bleeding patients?**	
	2	**What is the recommended dose of PCCs in bleeding patients?**	
	3	**Have PCCs been shown to be efficacious in bleeding patients?**	
	4	**What are the risks of using PCCs in bleeding patients?**	
	5	**Is there comparative data on the transfusion of PCCs compared with FFP in bleeding patients?**	
		("Wounds and Injuries"[MeSH] OR "Craniocerebral Trauma"[MeSH] OR "Cerebrovascular Trauma"[MeSH] OR "Multiple Trauma"[MeSH] OR "Trauma, Nervous System"[MeSH] OR "Head Injuries, Penetrating"[MeSH] OR "Carotid Artery Injuries"[MeSH] OR "Head Injuries, Closed"[MeSH] OR "Spinal Cord Injuries"[MeSH] OR "Traumatology"[MeSH] OR "Brain Injuries"[MeSH]) AND ("Thromboplastin"[MeSH] OR "Partial Thromboplastin Time"[MeSH] OR "Factor XI"[MeSH] OR "thromboplastin apoprotein, human"[Substance Name] OR "prothrombinase complex"[Substance Name] OR "Factor IXa"[MeSH] OR "Prothrombin Time"[MeSH] OR "Factor VII"[MeSH] OR "Factor IX"[MeSH] OR "prothrombin complex concentrates"[Substance Name])	Humans<br>English<br>2012-end
	6	**Does treatment with PCCs affect clinical outcome in patients who received pre-injury treatment with oral anticoagulants?**	
		("Wounds and Injuries"[MeSH] OR "Craniocerebral Trauma"[MeSH] OR "Cerebrovascular Trauma"[MeSH] OR "Multiple Trauma"[MeSH] OR "Trauma, Nervous System"[MeSH] OR "Head Injuries, Penetrating"[MeSH] OR "Carotid Artery Injuries"[MeSH] OR "Head Injuries, Closed"[MeSH] OR "Spinal Cord Injuries"[MeSH] OR "Traumatology"[MeSH] OR "Brain Injuries"[MeSH]) AND ("Thromboplastin"[MeSH] OR "Partial Thromboplastin Time"[MeSH] OR "Factor XI"[MeSH] OR "thromboplastin apoprotein, human"[Substance Name] OR "prothrombinase complex"[Substance Name] OR "Factor IXa"[MeSH] OR "Prothrombin Time"[MeSH] OR "Factor VII"[MeSH] OR "Factor IX"[MeSH] OR "prothrombin complex concentrates"[Substance Name]) AND "Anticoagulants"[Mesh]	Humans<br>English<br>10 years
		Desmopressin	
	1	**What are the indications for the use of desmopressin in a bleeding patient?**	

The European guideline on management of major bleeding and coagulopathy following trauma: Fourth edition

Rossaint R, Bouillon B, Cerny V, Coats TJ, Duranteau J, Fernández-Mondéjar E, Filipescu D, Hunt BJ, Komadina R, Nardi G, Neugebauer EAM, Ozier Y, Riddez L, Schultz A, Vincent J-L, Spahn DR

	2	**What is the recommended dose of desmopressin in a bleeding patient?**	
	3	**Has desmopressin been shown to be efficacious?**	
	4	**What are the risks of using desmopressin in bleeding patients?**	
		("Emergencies"[MeSH] OR "Emergency Treatment"[MeSH] OR "Emergency Medicine"[MeSH] OR "Emergency Medical Technicians"[MeSH] OR "Wounds and Injuries"[MeSH] OR "Craniocerebral Trauma"[MeSH] OR "Cerebrovascular Trauma"[MeSH] OR "Multiple Trauma"[MeSH] OR "Trauma, Nervous System"[MeSH] OR "Head Injuries, Penetrating"[MeSH] OR "Carotid Artery Injuries"[MeSH] OR "Head Injuries, Closed"[MeSH] OR "Spinal Cord Injuries"[MeSH] OR "Traumatology"[MeSH] OR "Brain Injuries"[MeSH]) AND "Deamino Arginine Vasopressin"[Mesh]	Humans<br>English<br>2012-end
		Calcium	
	1	**Under which circumstances should ionised calcium levels be monitored?**	
		("Emergencies"[MAJR] OR "Emergency Treatment"[MAJR] OR "Emergency Medicine"[MAJR] OR "Emergency Medical Technicians"[MAJR] OR "Wounds and Injuries"[MAJR] OR "Craniocerebral Trauma"[MAJR] OR "Cerebrovascular Trauma"[MAJR] OR "Multiple Trauma"[MAJR] OR "Trauma, Nervous System"[MAJR] OR "Head Injuries, Penetrating"[MAJR] OR "Carotid Artery Injuries"[MAJR] OR "Head Injuries, Closed"[MAJR] OR "Spinal Cord Injuries"[MAJR] OR "Traumatology"[MAJR] OR "Brain Injuries"[MAJR]) AND "Calcium"[MAJR]	Humans<br>English<br>2012-end
	2	**What is the appropriate use of calcium chloride in the bleeding trauma patient?**	
		("Emergencies"[MAJR] OR "Emergency Treatment"[MAJR] OR "Emergency Medicine"[MAJR] OR "Emergency Medical Technicians"[MAJR] OR "Wounds and Injuries"[MAJR] OR "Craniocerebral Trauma"[MAJR] OR "Cerebrovascular Trauma"[MAJR] OR "Multiple Trauma"[MAJR] OR "Trauma, Nervous System"[MAJR] OR "Head Injuries, Penetrating"[MAJR] OR "Carotid Artery Injuries"[MAJR] OR "Head Injuries, Closed"[MAJR] OR "Spinal Cord Injuries"[MAJR] OR "Traumatology"[MAJR] OR "Brain Injuries"[MAJR]) AND "Calcium/therapeutic use"[MAJR]	Humans<br>English<br>2012-end
		Novel anticoagulants	
	1	**Does pre-injury anticoagulant medication influence outcome?**	
		("Emergencies"[MAJR] OR "Emergency Treatment"[MAJR] OR "Emergency Medicine"[MAJR] OR "Emergency Medical Technicians"[MAJR] OR "Wounds and Injuries"[MAJR] OR "Craniocerebral Trauma"[MAJR] OR "Cerebrovascular Trauma"[MAJR] OR "Multiple Trauma"[MAJR] OR "Trauma, Nervous System"[MAJR] OR "Head Injuries, Penetrating"[MAJR] OR "Carotid Artery Injuries"[MAJR] OR "Head Injuries, Closed"[MAJR] OR "Spinal Cord Injuries"[MAJR] OR "Traumatology"[MAJR] OR "Brain Injuries"[MAJR]) AND ("Outcome Assessment (Health Care)"[Mesh] OR "Treatment Outcome"[Mesh] OR "Outcome and Process Assessment (Health Care)"[Mesh]) AND "Anticoagulants"[Mesh]	Humans<br>English
	2	**How can drug-induced coagulation disorders be detected and diagnosed?**	
		("Emergencies"[MAJR] OR "Emergency Treatment"[MAJR] OR "Emergency Medicine"[MAJR] OR "Emergency Medical Technicians"[MAJR] OR "Wounds and Injuries"[MAJR] OR "Craniocerebral Trauma"[MAJR] OR "Cerebrovascular Trauma"[MAJR] OR "Multiple Trauma"[MAJR] OR "Trauma, Nervous System"[MAJR] OR "Head Injuries, Penetrating"[MAJR] OR "Carotid Artery Injuries"[MAJR] OR "Head Injuries, Closed"[MAJR] OR "Spinal Cord Injuries"[MAJR] OR "Traumatology"[MAJR] OR "Brain Injuries"[MAJR]) AND ("Diagnosis"[Mesh] OR "Early Diagnosis"[Mesh]) AND "Blood Coagulation Disorders"[Mesh] AND "Anticoagulants"[Mesh]	Humans<br>English
	3	**How should drug-induced coagulopathies be managed?**	
	4	**Which antidotes can reverse the effects of pre-injury treatment with oral anticoagulants such as rivaroxaban, apixaban, endoxaban or dabigatran?**	

		("Emergencies"[MeSH] OR "Emergency Treatment"[MeSH] OR "Emergency Medicine"[MeSH] OR "Emergency Medical Technicians"[MeSH] OR "Wounds and Injuries"[MeSH] OR "Craniocerebral Trauma"[MeSH] OR "Cerebrovascular Trauma"[MeSH] OR "Multiple Trauma"[MeSH] OR "Trauma, Nervous System"[MeSH] OR "Head Injuries, Penetrating"[MeSH] OR "Carotid Artery Injuries"[MeSH] OR "Head Injuries, Closed"[MeSH] OR "Spinal Cord Injuries"[MeSH] OR "Traumatology"[MeSH] OR "Brain Injuries"[MeSH]) AND ("Anticoagulants"[Mesh] OR "rivaroxaban"[Supplementary Concept] OR "apixaban"[All Fields] OR "dabigatran"[Supplementary Concept] OR endoxaban[All Fields]) AND ("Therapeutics"[Mesh] OR "Emergency Treatment"[Mesh] OR "Antidotes" [Pharmacological Action])	Humans<br>English<br>2012-end
	**VII**	**GUIDELINE IMPLEMENTATION AND QUALITY CONTROL**	
	**1**	**Which massive transfusion guidelines have been published?**	
	**2**	**Which massive transfusion guidelines are used in clinical practice: institutional, national, international?**	
	**3**	**How uniform are massive transfusion guidelines?**	
	**4**	**Are massive transfusion guidelines adhered to in clinical practice?**	
		("Wounds and Injuries"[MeSH] OR "Craniocerebral Trauma"[MeSH] OR "Cerebrovascular Trauma"[MeSH] OR "Multiple Trauma"[MeSH] OR "Trauma, Nervous System"[MeSH] OR "Head Injuries, Penetrating"[MeSH] OR "Carotid Artery Injuries"[MeSH] OR "Head Injuries, Closed"[MeSH] OR "Spinal Cord Injuries"[MeSH] OR "Traumatology"[MeSH] OR "Brain Injuries"[MeSH]) AND ("Blood Transfusion"[Mesh] OR "Transfusion Medicine"[Mesh] OR "Platelet Transfusion"[Mesh] OR "Erythrocyte Transfusion"[Mesh] OR "Blood Component Transfusion"[Mesh]) AND ("Guideline" [Publication Type] OR "Evidence-Based Emergency Medicine"[Mesh] OR "Evidence-Based Practice"[Mesh] OR "Guideline Adherence"[Mesh] OR "Evidence-Based Medicine"[Mesh])	Humans<br>English<br>10 years
	**5**	**Has clinical practice in the management of polytrauma patients changed?**	
		("Emergencies"[MeSH] OR "Emergency Treatment"[MeSH] OR "Emergency Medicine"[MeSH] OR "Emergency Medical Technicians"[MeSH] OR "Wounds and Injuries"[MeSH] OR "Craniocerebral Trauma"[MeSH] OR "Cerebrovascular Trauma"[MeSH] OR "Multiple Trauma"[MeSH] OR "Trauma, Nervous System"[MeSH] OR "Head Injuries, Penetrating"[MeSH] OR "Carotid Artery Injuries"[MeSH] OR "Head Injuries, Closed"[MeSH] OR "Spinal Cord Injuries"[MeSH] OR "Traumatology"[MeSH] OR "Brain Injuries"[MeSH]) AND "Physician's Practice Patterns"[Mesh]	Humans<br>English<br>2012-end
	**6**	**Does the implementation and use of multi-disciplinary trauma treatment simulation improve outcomes?**	
	**7**	**Which types of educational measure are effective in improving outcomes in trauma patients?**	
		("Multiple Trauma"[MeSH] OR "Traumatology"[MeSH]) AND ("Education"[Mesh] OR "Patient Simulation"[Mesh])	Humans<br>English<br>10 years
	**8**	**Does the implementation and use of treatment 'bundles' improve outcomes?**	
		("Emergencies"[MeSH] OR "Emergency Treatment"[MeSH] OR "Emergency Medicine"[MeSH] OR "Emergency Medical Technicians"[MeSH] OR "Wounds and Injuries"[MeSH] OR "Craniocerebral Trauma"[MeSH] OR "Cerebrovascular Trauma"[MeSH] OR "Multiple Trauma"[MeSH] OR "Trauma, Nervous System"[MeSH] OR "Head Injuries, Penetrating"[MeSH] OR "Carotid Artery Injuries"[MeSH] OR "Head Injuries, Closed"[MeSH] OR "Spinal Cord Injuries"[MeSH] OR "Traumatology"[MeSH] OR "Brain Injuries"[MeSH]) AND "Patient Care Bundles"[Mesh]	Humans<br>English<br>10 years
